# Structural Design, Synthesis, and Preliminary Biological Evaluation of Novel Dihomooxacalix[4]arene-Based Anti-tumor Agents

**DOI:** 10.3389/fchem.2019.00856

**Published:** 2019-12-13

**Authors:** Lin An, Chan Wang, Lili Han, Jiadong Liu, Tonghui Huang, Youguang Zheng, Chaoguo Yan, Jing Sun

**Affiliations:** ^1^College of Pharmacy, Xuzhou Medical University, Xuzhou, China; ^2^Jiangsu Key Laboratory of New Drug Research and Clinical Pharmacy, Xuzhou Medical University, Xuzhou, China; ^3^Children's Hospital Affiliated to Zhengzhou University, Zhengzhou, China; ^4^College of Chemistry and Chemical Engineering, Yangzhou University, Yangzhou, China

**Keywords:** supramolecular chemotherapy, calixarene, structural optimization, dihomooxacalix[4]arene, X-ray diffraction, biological evaluation

## Abstract

Calixarene and its derivatives have extensively served as promising anti-tumor agents. Previously, we have synthesized a series of calix[n]arene polyhydroxyamine derivatives (*n* = 4, 6, 8) and found that 5,11,17,23-*tetra-tert*-butyl-25,27-bis [N-(2-hydroxyethyl)aminocarbonylmethoxyl] calix[4]arene **(CLX-4)** displayed significant effect toward SKOV3, A549, SW1990, HeLa, Raji, and MDA-MB-231 cancer cells. In the present work, we find a replacement of calix[4]arene bone and synthesized 19 novel structurally related dihomooxacalix[4]arene amide derivatives **4A−4S** to optimize its efficacy. Their abilities to induce cytotoxicity in human lung carcinoma (A549) cells, breast cancer (MCF-7) cells, cervical cancer (HeLa) cells, hepatocellular carcinoma (HepG2) cells, as well as human umbilical vein endothelial (HUVEC) cells are evaluated *in vitro*. Encouraging results show that the majority of dihomooxacalix[4]arene amide derivatives are effective at inhibiting A549 cell proliferation with the corresponding IC_50_ ranging from 0.6 to 20.1 μM. In particular, compounds **4A**, **4D**, and **4L** explore markedly increased potency (IC_50_ value is 2.0 ± 0.5 μM, 0.7 ± 0.1 μM, and 1.7 ± 0.4 μM) over the cytotoxicity profiles of control **CLX-4**, whose IC_50_ value is 2.8 ± 0.3 μM. More interestingly, **4A** also demonstrates the perfect cytotoxic effect against MCF-7, HeLa, and HepG2 cells with IC_50_ values of 1.0 ± 0.1 μM, 0.8 ± 0.2 μM, and 2.7 ± 0.4 μM. In addition, the results proved that our synthesized **4A** has much lower toxicity (41%) to normal cells at a concentration of 10 μM than that of **4D** (90%). To reveal the mechanisms, the key indicators including the cell cycle and apoptosis are observed by the flow cytometry analysis in MCF-7 cells. The results demonstrate that both **4A** and **4D** can induce the MCF-7 cell cycle arrest in G0/G1 phase and cell apoptosis. Therefore, our finding proves that the dihomooxacalix[4]arene amide derivatives are convenient platforms for potential supramolecular anticancer agents.

## Introduction

Supramolecules generally come from the aggregation of two or more molecules based on non-covalent bond forces (Lehn, 1988; Guo et al., 2018). They usually have hydrophobic cavities in which the guests can be embedded, such as crown ethers (Flink and Reinhoudt, 1999; Kralj et al., 2008; Yokoyama and Mizuguchi, 2019), cyclodextrins (Valle, 2004; Stella and He, 2008; Zhang Y. M. et al., 2019), calixarenes (Sun et al., 2009a,b; Böhmer, 2010), cucurbiturils (Lee et al., 2003; Kim et al., 2007; Bauer et al., 2019) and pillararenes (Hu et al., 2012; Sun et al., 2018, 2019; Chen et al., 2019; Zhang R. et al., 2019).

**Figure 1:**
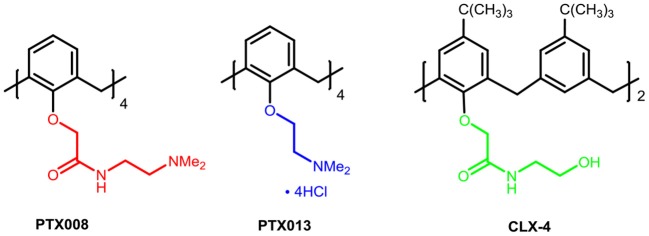
The chemical structures of calix[4]arene-based anti-tumor agents.

Among those supramolecules, calixarenes are cyclic oligomers composed of phenolic units linked by methylene in the ortho positions, which are considered to be the important class representing the third generation of host–guest supramolecular chemistry. A majority of studies on calixarenes have focused on the fields of molecular recognition due to their flexible nature of the basic moiety preferable for binding and transporting ions and neutral molecules (Ludwig and Dzung, 2002; Mutihac et al., 2011; Gómez-Machuca et al., 2014; Zadmard and Alavijeh, 2014; Patra et al., 2019). In addition, calixarenes have many other structural characteristics including the easy modification of their basic core and rim via covalent attachment to various chemical scaffolds, the limited toxicity and immune responses (Geraci et al., 2008). The above advantages make these cyclic oligomers ideal for the design of new drugs and building blocks for drug carriers in biomedical fields (Da Silva et al., 2004; Hussain et al., 2017). In this regard, calixarenes and their derivatives are currently being studied and used in a variety of medicinal applications beyond their traditional place in chemistry. They could be served as chemotherapy agent (Yousaf et al., 2015; Naseer et al., 2017; Zhou et al., 2017), including antibacterials (Soares et al., 2014; Muneer et al., 2017; Ali et al., 2018; Consoli et al., 2018), antivirals (Motornaya et al., 2006; Mourer et al., 2010), antimalarials (Shah et al., 2016), and anti-inflammatory (Granata et al., 2017) enzymatic inhibitors (Läppchen et al., 2015). Moreover, they especially can be used as anti-tumor drugs (Consoli et al., 2004; Hulíková et al., 2010; Neagu et al., 2010; Nasuhi Pur and Dilmaghani, 2014); which have gained considerable attention from us. Among these works, ureido-glycocalix[8]arene carrying N-acetyl-D-glucosamine residue groups has been reported, which inhibited C6 glioma cell migration and proliferation with independence of the N-acetyl-D-glucosamine residues (Sansone et al., 2008; Viola et al., 2010a,b). Dings et al. worked continuously on modifications and mechanism of calix[4]arene-based anti-tumor agents, identified galectin-1 (gal-1) as the molecular target, and explored compounds PTX008, PTX013 as potent anti-tumor agents (Figure 1) (Dings et al., 2012a,b, 2013; Koonce et al., 2017). Excitingly, PTX008 has been in a human Phase I clinical trial. The latest study further illustrated that PTX008 was an allosteric inhibitor that inhibits Galectin-1 due to BP-ALL survival (Paz et al., 2018).

**Figure 2:**
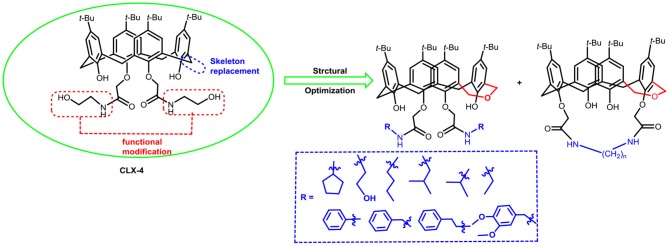
Structural optimization design of dihomooxacalix[4]arene amide derivatives.

In our previous work, we synthesized calix[4]arene polyhydroxyamine derivative **CLX-4** (Figure 1), which has been tested as a candidate anti-tumor drug with IC_50_ value against A549, SKOV3, SW1990, Hela, Raji, and MDA-MB-231 cell lines ranging from 2.8 to 5.3 μM (An et al., 2016). **CLX-4** obviously exhibited equal cytotoxic effects to those of PTX008. This inspired us to have some structural optimizations, as shown in Figure 2.

In our design (Figure 2), one possible strategy is to find alternatives to replace the material structure of **CLX-4**. Taking into account the high structural similarity, dihomooxacalix[4]arene is the closest structurally calix[4]arene analog with only one CH_2_OCH_2_ unit taking the place of CH_2_ bridge, which results in improved conformational flexibility and the superior geometric shapes. Dihomooxacalix[4]arene was initially reported by Gutsche et al. (1981). However, over the past several years, dihomooxacalix[4]arene macrocycles and their functional derivatives were used for ion-binding application (Marcos et al., 2006, 2014; Gaeta et al., 2012; Talotta et al., 2016; Liu et al., 2017, 2018; An et al., 2018). It would seem surprising that as yet few works are so far known about the bioactivity-based approach (Harris, 1995). Therefore, dihomooxacalix[4]arenes are a particularly promising alternative to replace the calix[4]arene as the new drug bone. The other way is to chemically modify the important functional OH-CH_2_-CH_2_-NH-C = O group at the lower rim of **CLX-4**, which can be expected to introduce special groups with different polarity, hydrophilicity, and compatibility, leading to screening out the optimization. Appropriate R groups can be introduced to replace the 2-hydroxyethyl group linked on the aminocarbonyl dihomooxacalix[4] arene, which probably affects the anti-tumor activity by means of rigidity, electron density, flexibility, polarity and stability. To compare the influence of activity and explore the structure–activity relationship, R position attached to the acylamino unit is varied from methyl, ethyl, propyl, isopropyl, butyl, pentyl, to cyclopentyl groups, In addition, we also intend to build the spacer chains involved in the link of amino moieties on the dihomooxacalix[4]arene scaffold. As to verify our hypothesis and extend our previous work, herein we report on the synthesis, X-ray structures of a series of novel structural dihomooxacalix[4]arene amide derivatives, as well as the cell-based studies related to structure–activity relationship.

## Materials and Methods

### Synthesis and Characterization

#### Synthesis of Dihomooxacalix[4]arene Amide Derivatives 4A−4S

*p-tert*-butyl dihomooxacalix[4]arenes 1 was synthesized according to Gutsche's method (Gutsche et al., 1981). All other reagents and solvents were commercially available reagents with analytical grade and used without further purification. The products were purified by recrystallization or using preparative separations in flash column chromatography. Reactions were monitored by thin-layer chromatography (TLC) on 2.5 mm Merck silica gel F254 strips. Melting points were determined with capillaries on an YRT-3 microscope apparatus and were uncorrected. All ^1^H NMR and ^13^C NMR spectra were recorded at 400 MHz on a Bruker AVANCE II 400 spectrometer. IR spectra were obtained on a Nicolet FT-IR 8400 spectrometer (KBr disc). High Resolution Mass Spectrometry were carried out on (UHR-TOF) maXis 4G mass spectrometer.

### General Procedure for the Synthesis of Dihomooxacalix[4]arene Ester 2A

Under ultrasound irradiation, a mixture of *p-tert*-butyl dihomooxacalix[4]arene 1 (6.78 g, 10 mmol), bromoethylacetate (6.68 ml, 60 mmol), and acetone (100 ml) was stirred at 55°C. The reaction was monitored by TLC until the start material 1 disappeared. The mixture was poured into a large amount of water and extracted with chloroform. The organic layers were collected, dried over anhydrous sodium sulfate, concentrated *in vacuo*, and further purified by silica gel column chromatography (1:4 ethyl acetate-petroleum ether) to provide the product 2A as the white solid, which was used in the next step.

### General Procedure for the Synthesis of Dihomooxacalix[4]arene Amide 3A

Ester 2A (0.230 g, 0.3 mmol) was dissolved in dichloromethane (2 ml), and ethanolamine (0.037 g, 0.6 mmol) was added to the solution. Then, the resulting mixture was stirred at room temperature for 24 h. It was concentrated *in vacuo*, washing with 95% ethanol twice to give the solid precipitate, followed by filtration, purified by silica gel column chromatography (15:1 v/v, dichloromethane/methanol) to afford the desired 3A in quantitative yield.

### General Procedure for the Synthesis of Dihomooxacalix[4]arene Amides 4A−4B

A solution of ester 2A (0.425 g, 0.5 mmol) and hydroxylamine (0.244 g, 4 mol) or dihydroxylamine (0.421 g, 4 mol) in refluxing ethanol/toluene (50 ml, v/v = 1:1) for 72 h. After the completion of the reaction (TLC), the solution was concentrated *in vacuo*, leaving the pale oil residue, which was washed with 95% ethanol to give the solid precipitate, followed by filtration, purified by silica gel column chromatography to yield 4A or 4B as a white solid.

### General Procedure for the Synthesis of Dihomooxacalix[4]arene Amides 4C−4D

A solution of 1,4-diaminobutane or 1,6-diaminohexane (2 ml) in methanol (2 ml) kept at 0°C was added dropwise solution of ester 2A (0.425 g, 0.5 mmol) in methanol (1 ml) for 30 min. The ice bath was removed and the reaction mixture was warmed to room temperature and stirred for 24 h. The solution was concentrated *in vacuo*, and the residue was treated with cool water to afford the pale yellow solid precipitate, further purified by silica gel flash column chromatography to yield 4C−4D.

### General Procedure for the Synthesis of Bridged Dihomooxacalix[4]arene Amides 4E−4G

A mixture of ester 2A (0.425 g, 0.5 mmol) and 2 ml of ethylenediamine (or 1,4-diaminobutane, 1,6-diaminohexane) was dissolved in ethanol (5 ml) and refluxed for 24 h. When the reaction was complete, the solution was concentrated *in vacuo*, followed by dropwise distilled water was added. The crude product was extracted and filtered to give the pale yellow solid precipitate, further purified by silica gel flash column chromatography to yield compound 4E−4G.

### General Procedure for the Synthesis of Dihomooxacalix[4]arene Amides 4H−4P

A solution of ethylamine hydrochloride (2 ml) in ethanol (2 ml), kept at 0°C, was added dropwise to a solution of ester 2A (0.425 g, 0.5 mmol) in ethanol (1 ml) for 30 min. Then, the mixture was stirred at room temperature overnight. After the completion of the reaction, the solvent was removed *in vacuo*, and then the residue was treated with cool water to give the solid precipitate, further purified by silica gel flash column chromatography to yield compound 4H. Compound 4I−4P was prepared by a similar procedure.

### General Procedure for the Synthesis of 4Q−4S

A solution of 3A with cyclopentylamine (2 ml) was stirred for 4 h at room temperature. Then, the solution was concentrated *in vacuo* to give the crude product, followed by the purification with silica gel flash chromatography to give the compound 4Q, as white powder.

Compound **4R−4S** was prepared by a similar procedure.

**7,13,19,25-tetra-*tert*-butyl-28,30-di-hydroxy-27,29-di-(ethoxycarbonylmethoxyl)-2,3-dihomo-3-oxacalix[4]arene (2A):** White solid, m.p. 168.7–171.4°C, yield: 95.6% (Figure S1); ^1^H NMR (400 MHz, CDCl_3_) *δ* (ppm): 1.11, 1.19, 1.23, 1.26 (4s, 36H), 1.30 (t, 3H, *J* = 7.2 Hz), 1.38 (t, 3H, *J* = 7.2 Hz), 3.28 (d, 1H, *J* = 12.8 Hz), 3.37 (d, 1H, *J* = 13.6 Hz), 3.46 (d, 1H, *J* = 13.2 Hz), 4.21 (d, 1H, *J* = 9.2 Hz), 4.24 (dd, 1H, *J*_1_ = 7.2 Hz, *J*_2_ = 1.2 Hz), 4.28 (dd, 1H, *J*_1_ = 7.2 Hz, *J*_2_ = 1.2 Hz), 4.31 (d, 1H, *J* = 13.2 Hz), 4.34–4.41 (m, 3H), 4.46 (d, 1H, *J* = 12.8 Hz), 4.52 (d, 1H, *J* = 15.6 Hz), 4.67 (d, 1H, *J* = 10.4 Hz), 4.71 (d, 1H, *J* = 13.6 Hz), 4.81 (d, 1H, *J* = 15.6 Hz), 4.98 (d, 1H, *J* = 10.0 Hz), 5.35 (d, 1H, *J* = 9.2 Hz), 5.53 (d, 1H, *J* = 16.0 Hz), 6.89 (d, 1H, *J* = 2.4 Hz), 6.96 (d, 1H, *J* = 2.4 Hz), 6.99 (d, 1H, *J* = 2.4 Hz), 7.03 (q, 2H, *J* = 2.4 Hz), 7.07 (d, 1H, *J* = 2.8 Hz), 7.30 (s, 1H), 7.40 (d, 1H, *J* = 2.4 Hz), 7.77 (s, 1H); ^13^C NMR (100 MHz, CDCl_3_) *δ* (ppm): 14.3, 31.1, 31.4, 31.5, 31.6, 33.8, 34.1, 60.9, 61.4, 71.6, 72.1, 72.3, 122.6, 124.1, 125.0, 125.3, 125.4, 125.8, 126.0, 126.9, 127.6, 127.7, 128.2, 129.5, 129.8, 132.3, 132.7, 133.7, 141.1, 142.0, 146.5, 147.5, 149.4, 150.1, 152.9, 155.0, 168.8, 170.5; IR (KBr, cm^−1^) *v* 3313, 2959, 2868, 1665, 1483, 1364, 1298, 876.

**7,13,19,25-tetra-*tert*-butyl-28,30-di-hydroxy-27-(ethoxycarbonylmethoxyl)-29-(N-(2-hydroxyethyl) amino carbonyl methoxyl)-2,3-dihomo-3-oxacalix[4]arene (3A):** White solid, yield: 85.1%, m.p. 188.6–191.8°C (Figure S2); ^1^H NMR (400 MHz, CDCl_3_) *δ* (ppm): 1.14, 1.22, 1.26 (3s, 36H), 1.33 (t, 3H, *J* = 7.2 Hz), 3.36 (d, 1H, *J* = 13.6 Hz), 3.42 (d, 1H, *J* = 13.2 Hz), 3.49 (d, 1H, *J* = 13.2 Hz), 3.67–3.87 (m, 4H), 4.14 (d, 1H, *J* = 13.2 Hz), 4.20 (d, 1H, *J* = 9.2 Hz), 4.24–4.35 (m, 4H), 4.39 (d, 1H, *J* = 15.6 Hz), 4.47 (d, 2H, *J* = 13.6 Hz), 4.64 (d, 1H, *J* = 16.0 Hz), 4.76 (d, 1H, *J* = 15.2 Hz), 4.94 (d, 1H, *J* = 9.6 Hz), 5.08–5.13 (m, 2H), 6.91 (d, 1H, *J* = 2.4 Hz), 6.98 (d, 1H, *J* = 2.4 Hz), 7.00 (d, 1H, *J* = 2.4 Hz), 7.10–7.12 (q, 3H, J = 2.4 Hz), 7.23 (d, 1H, *J* = 2.0 Hz), 7.43(d, 1H, *J* = 2.4 Hz), 7.77 (s, 1H), 8.85 (t, 1H, *J* = 5.2 Hz; ^13^C NMR (100 MHz, CDCl_3_) *δ* (ppm): 14.2, 30.8, 30.9, 31.0, 31.1, 31.3, 31.5, 31.6, 31.7, 32.1, 33.8, 33.9, 34.2, 42.8, 61.4, 62.8, 71.3, 71.9, 72.3, 74.1, 122.8, 124.5, 125.3, 125.4, 125.8, 126.2, 126.4, 127.1, 128.0, 128.1, 129.1, 129.6, 131.7, 132.7, 133.9, 142.4, 143.0, 147.5, 147.8, 148.9, 149.7, 152.0, 154.4, 170.1, 170.3; IR (KBr, cm^−1^) ν 3433, 2961, 2868, 1757, 1670, 1485, 1209, 1065, 874; MS (m/z): HRMS (ESI) Calcd for C_53_H_71_NNaO_9_ ([M+Na]^+^): 888.5021, found: 888.5029.

**7,13,19,25-tetra*-tert*-butyl-28,30-di-hydroxy-27,29-di-(N-(2-hydroxyethyl)-aminocarbonylmethoxyl)-2,3-dihomo-3-oxacalix[4]arene (4A):** White solid, m.p. 139.7–142.3°C, yield: 87.2% (Figure S3); ^1^H NMR (400 MHz, CDCl_3_) *δ* (ppm) 1.03, 1.20, 1.26, 1.29 (4s, 36H), 3.44–3.53 (m, 5H), 3.59–3.64 (m, 2H), 3.79 (s, 4H), 4.17–4.29 (m, 4H), 4.38 (d, 1H, *J* = 10.0 Hz), 4.54 (dd, 1H, *J*_1_ = 15.2 Hz, *J*_2_ = 5.2 Hz), 4.60–4.68 (m, 4H), 4.84 (d, 1H, *J* = 10.0 Hz), 6.85 (d, 1H, *J* = 1.6 Hz), 6.92 (d, 1H, *J* = 2.4 Hz), 7.02 (d, 2H, *J* = 2.0 Hz), 7.16 (d, 1H, *J* = 2.0 Hz), 7.22 (d, 1H, *J* = 2.0 Hz), 7.24 (d, 1H, *J* = 2.0 Hz), 7.31 (s, 1H), 7.38 (d, 2H, *J* = 2.4 Hz), 8.78 (t, 1H, *J* = 5.2 Hz), 8.84 (t, 1H, *J* = 5.2 Hz); ^13^C NMR (100 MHz, CDCl_3_) *δ* (ppm): 31.0, 31.2, 31.4, 31.5, 31.9, 34.0, 34.1, 34.2, 42.5, 62.0, 62.2, 70.8, 72.1, 73.9, 74.4, 76.7, 77.0, 77.2, 77.3, 122.7, 124.7, 125.9, 126.0, 126.3, 127.1, 127.4, 127.7, 127.8, 128.0, 128.8, 129.3, 131.9, 132.1, 133.6, 143.1, 143.8, 148.1, 148.4, 148.8, 149.9, 151.2, 152.6, 169.4, 169.7; IR (KBr, cm^−1^) *v* 3389, 3366, 2959, 2870, 1666, 1545, 1485, 1447, 1362, 874; MS (m/z): HRMS (ESI) Calcd for C_53_H_72_N_2_NaO_9_ ([M+Na]^+^): 903.5130, found: 903.5145.

**7,13,19,25-tetra*-tert*-butyl-28,30-di-hydroxy-27,29-di-(N,N-bis(2-hydroxyethyl)-aminocarbonylmethoxyl)-2,3-dihomo−3-oxacalix[4]arene (4B):** White solid, m.p. 210.7–212.5°C, yield: 86.7% (Figure S4); ^1^H NMR (400 MHz, DMSO-*d*_6_) *δ* (ppm): 1.11, 1.18, 1.20, 1.24 (4s, 36H), 3.42–3.49 (m, 6H), 3.54–3.62 (m, 10H), 3.67 (s, 2H), 3.87–3.91 (m, 1H), 4.19 (d, 1H, *J* = 9.2 Hz), 4.26 (d, 1H, *J* = 10.0 Hz), 4.36 (dd, 2H, *J*_1_ = 12.8 Hz, *J*_2_ = 6.0 Hz), 4.56 (d, 1H, *J* = 12.8 Hz), 4.66 (d, 2H, *J* = 13.2 Hz), 4.74 (d, 2H, *J* = 9.2 Hz), 4.78 (s, 1H), 4.90 (s, 1H), 4.97 (s, 1H), 5.03 (s, 1H), 5.06 (d, 1H, *J* = 4.8 Hz), 5.32 (d, 1H, *J* = 13.2 Hz), 6.89 (d, 1H, *J* = 2.4 Hz), 7.00 (d, 1H, *J* = 2.0 Hz), 7.02 (d, 1H, *J* = 2.0 Hz), 7.13 (s, 1H), 7.16 (s, 1H), 7.22 (d, 1H, *J* = 2.0 Hz), 7.36 (d, 1H, *J* = 2.0 Hz), 7.48 (d, 1H, *J* = 2.0 Hz), 7.71 (s, 1H), 8.61 (s, 1H); ^13^C NMR (100 MHz, DMSO-*d*_6_) *δ* (ppm): 31.4, 31.6, 31.8, 31.9, 34.0, 34.3, 59.1, 122.8, 125.5, 125.7, 126.6, 126.7, 127.2, 127.9, 129.8, 132.9, 133.4, 134.5, 140.8, 141.0, 146.0, 146.7, 150.0, 152.1, 152.4, 155.3, 169.3, 169.6; IR (KBr, cm^−1^) *v* 3389, 3045, 2957, 2870, 1643, 1485, 1443, 1364, 876; MS (m/z): HRMS (ESI) Calcd for C_57_H_80_N_2_NaO_11_ ([M+Na]^+^): 991.5654, found: 991.5674.

**7,13,19,25-tetra*-tert*-butyl-28,30-di-hydroxy-27,29-di-(N-(4-aminobutyl)-aminocarbonylmethoxyl)-2,3-dihomo-3-oxacalix[4] arene (4C):** White solid, m.p. 129.4–131.2°C, yield: 96.2% (Figure S5); ^1^H NMR (400 MHz, CDCl_3_) *δ* (ppm): 1.16, 1.24, 1.25, 1.27 (4s, 36H), 1.32 (d, 2H, *J* = 11.2 Hz), 1.40–1.43 (m, 2H), 1.45–1.53 (m, 3H), 1.57–1.73 (m, 4H), 2.57 (t, 2H, *J* = 7.2 Hz), 2.68 (t, 2H, *J* = 6.8 Hz), 3.18–3.26 (m, 2H), 3.43 (t, 2H, *J* = 12.0 Hz), 3.52–3.66 (m, 3H), 4.10 (d, 1H, *J* = 13.6 Hz), 4.16–4.22 (m, 2H), 4.35 (s, 1H), 4.38–4.40 (m, 2H), 4.44 (d, 1H, *J* = 9.2 Hz), 4.50 (d, 1H, *J* = 11.2 Hz), 4.58 (d, 1H, *J* = 10.0 Hz), 4.80 (dd, 2H, *J*_1_ = 19.2 Hz, *J*_2_ = 15.2 Hz), 4.96 (d, 1H, *J* = 10.4 Hz), 6.87 (d, 1H, *J* = 2.4 Hz), 7.01 (d, 1H, *J* = 2.4 Hz), 7.04 (d, 1H, *J* = 2.0 Hz), 7.14 (d, 1H, *J* = 2.4 Hz), 7.16 (d, 1H, *J* = 2.4 Hz), 7.23 (t, 2H, *J* = 2.4 Hz), 7.46 (d, 1H, *J* = 2.4 Hz), 8.24 (s, 1H), 8.74 (t, 1H, *J* = 4.2 Hz), 9.00 (t, 1H, *J* = 4.2 Hz); ^13^C NMR (100 MHz, CDCl_3_) *δ* (ppm): 26.4, 26.9, 31.0, 31.1, 31.3, 31.5, 32.4, 33.9, 34.0, 34.3, 39.2, 39.5, 41.9, 42.0, 71.2, 71.9, 73.8, 74.5, 122.2, 124.4, 125.7, 126.0, 126.9, 127.0, 127.2, 127.3, 127.4, 127.6, 128.6, 129.6, 131.5, 132.4, 133.8, 143.0, 143.7, 148.0, 148.6, 148.9, 149.5, 151.4, 153.2, 168.0, 168.5; IR (KBr, cm^−1^) *v* 3369, 3194, 2959, 2866, 1674, 1483, 1296, 874; MS (m/z): HRMS (ESI) Calcd for C_57_H_83_N_4_O_7_ ([M+H]^+^): 935.6256, found: 935.6284.

**7,13,19,25-tetra-*tert*-butyl-28,30-di-hydroxy-27,29-di-(N-(6-aminohexyl)-aminocarbonylmethoxyl)-2,3-dihomo-3-oxa calix[4] arene (4D):** White solid, m.p. 123.3–125.9°C, yield: 63.3% (Figure S6); ^1^H NMR (400 MHz, CDCl_3_) *δ* (ppm): 1.18 (s, 9H), 1.27 (brs, 18H), 1.29 (s, 9H), 1.33 (d, 10H, *J* = 12.0 Hz), 1.57–1.69 (m, 5H), 1.99 (d, 2H, *J* = 3.6 Hz), 2.52–2.55 (m, 2H), 2.60–2.63 (m, 2H), 2.70 (t, 1H, *J* = 7.2 Hz), 3.17–3.28 (m, 3H), 3.42–3.68 (m, 6H), 4.11 (d, 1H, *J* = 13.6 Hz), 4.16–4.23 (m, 2H), 4.37 (s, 1H), 4.40–4.44 (m, 2H), 4.49 (d, 1H, *J* = 14.8 Hz), 4.62 (d, 1H, *J* = 9.6 Hz), 4.80 (t, 2H, *J* = 15.2 Hz), 4.96 (d, 1H, *J* = 10.4 Hz), 6.89 (d, 1H, *J* = 2.0 Hz), 7.04 (d, 1H, *J* = 2.0 Hz), 7.05 (d, 1H, *J* = 2.4 Hz), 7.16 (d, 1H, *J* = 2.0 Hz), 7.18 (d, 1H, *J* = 2.0 Hz), 7.22–7.24 (m, 2H), 7.48 (d, 1H, *J* = 2.4 Hz), 8.24 (s, 1H), 8.70 (t, 1H, *J* = 5.6 Hz), 8.96 (t, 1H, *J* = 6.0 Hz); ^13^C NMR (100 MHz, CDCl_3_) *δ* (ppm): 26.5, 26.9, 27.0, 29.5, 31.1, 31.3, 31.5, 33.6, 33.9, 34.3, 42.0, 42.1, 122.3, 124.4, 127.2, 127.4, 128.6, 131.5, 132.3, 133.7, 142.9, 148.6, 148.9, 151.5, 153.2, 167.9, 168.4; IR (KBr, cm^−1^) *v* 3350, 3215, 2957, 2862, 1674, 1483, 1298, 818; MS (m/z): HRMS (ESI) Calcd for C_61_H_91_N_4_O_7_ ([M+H]^+^): 991.6882, found: 991.6883.

**7,13,19,25-tetra-*tert*-butyl-28,30-di-hydroxy-27,29-N,N-(ethane-1,2-diyl)-aminocarbonylmethoxyl)-2,3-dihomo-3-oxa calix[4] arene (4E):** White solid, m.p. 204.8–206.3°C, yield: 86.6% (Figure S7); ^1^H NMR (400 MHz, CDCl_3_) *δ* (ppm): 1.16, 1.24, 1.25, 1.26 (4s, 36H), 3.10–3.23 (m, 2H), 3.40 (dd, 2H, *J*_1_ = 13.2 Hz, *J*_2_ = 2.8 Hz), 3.58 (d, 1H, *J* = 13.6 Hz), 4.01 (d, 1H, *J* = 13.6 Hz), 4.14–4.22 (m, 3H), 4.24 (d, 1H, *J* = 9.6 Hz), 4.28 (d, 1H, *J* = 14.0 Hz), 4.38–4.46 (m, 3H), 4.67 (d, 1H, *J* = 14.4 Hz), 4.74–4.80 (m, 2H), 4.89 (d, 1H, *J* = 9.6 Hz), 6.89 (d, 1H, *J* = 2.4 Hz), 7.02 (dd, 2H, *J*_1_ = 4.0 Hz, *J*_2_ = 2.4 Hz), 7.08 (d, 1H, *J* = 2.4 Hz), 7.13 (d, 2H, *J* = 3.2 Hz), 7.25 (s, 1H), 7.28 (d, 1H, *J* = 2.4 Hz), 7.53, 8.11 (2s, 2H), 8.30–8.33 (m, 1H), 8.60–8.63 (m, 1H); ^13^C NMR (100 MHz, CDCl_3_) *δ* (ppm): 29.5, 30.5, 31.2, 31.3, 31.5, 32.6, 33.9, 34.3, 39.2, 39.6, 71.3, 72.8, 73.7, 74.0, 122.6, 124.4, 125.9, 126.1, 126.2, 126.4, 126.8, 127.1, 127.8, 128.0, 128.9, 129.6, 131.4, 131.9, 143.0, 143.1, 147.5, 147.8, 148.7, 149.1, 151.9, 152.2, 167.8, 168.7; IR (KBr, cm^−1^) *v* 3373, 3180, 3049, 2961, 2868, 1692, 1530, 1483, 1364, 1296, 874; MS (m/z): HRMS (ESI) Calcd for C_51_H_66_N_2_NaO_7_ ([M+Na]^+^): 841.4762, found: 841.4771.

**7,13,19,25-tetra-*tert*-butyl-28,30-di-hydroxy-27,29-N,N-(propane-1,3-diyl)-aminocarbonylmethoxyl)-2,3-dihomo-3-oxa calix[4] arene (4F):** White solid, m.p. 189.5–191.4°C, yield: 92.0%; (Figure S8) ^1^H NMR (400 MHz, CDCl_3_) *δ* (ppm): 1.16, 1.24, 1.25, 1.26 (4s, 36H), 2.29–2.38 (m, 2H), 3.26 (s, 2H), 3.42 (dd, 2H, *J*_1_ = 12.4 Hz, *J*_2_ = 1.2 Hz), 3.57 (d, 1H, *J* = 13.6 Hz), 3.66 (d, 1H, *J* = 14.8 Hz), 3.76 (d, 1H, *J* = 16.8 Hz), 4.05 (d, 1H, *J* = 14.0 Hz), 4.15 (d, 1H, *J* = 13.2 Hz), 4.21–4.26 (m, 2H), 4.31 (d, 1H, *J* = 12.8 Hz), 4.36–4.22 (m, 2H), 4.70–4.74 (m, 2H), 4.80–4.87 (m, 2H), 6.90 (s, 1H), 7.03 (s, 2H), 7.13 (d, 2H, *J* = 13.2 Hz), 7.32 (s, 1H), 7.50 (s, 1H), 8.16 (s, 1H), 8.50 (s, 1H), 8.69 (s, 1H); ^13^C NMR (100 MHz, CDCl_3_) *δ* (ppm): 23.2, 30.0, 31.1, 31.3, 31.5, 32.3, 33.9, 34.3, 36.1, 71.0, 72.4, 73.8, 74.0, 122.7, 124.7, 125.9, 126.2, 126.3, 126.5, 126.8, 126.9, 127.5, 128.0, 129.1, 129.6, 131.2, 131.9, 133.7, 143.1, 143.3, 148.0, 148.2, 148.7, 149.1, 151.8, 152.6, 168.2, 169.3; IR (KBr, cm^−1^) *v* 3377, 3049, 2961, 2868, 1690, 1599, 1483, 1443, 1298, 874; MS (m/z): HRMS (ESI) Calcd for C_52_H_68_N_2_NaO_7_ ([M+Na]^+^): 855.4919, found: 855.4922.

**7,13,19,25-tetra-*tert-*butyl-28,30-di-hydroxy-27,29-27,29-N,N-(butane-1,4-diyl)–aminocarbonylmethoxyl)-2,3-dihomo-3-oxa calix[4] arene (4G):** White solid, m.p. 157.6–158.7°C, yield: 91.9% (Figure S9); ^1^H NMR (400 MHz, DMSO-*d*_6_) *δ* (ppm): 1.09, 1.19, 1.22, 1.23 (4s, 36H), 1.63 (d, 4H, *J* = 4.4 Hz), 3.42–3.52 (m, 6H), 4.21 (d, 1H, *J* = 9.6 Hz), 4.27–4.34 (m, 4H), 4.36–4.42 (m, 4H), 4.54 (d, 1H, *J* = 14.0 Hz), 4.73 (d, 1H, *J* = 9.6 Hz), 4.79 (d, 1H, *J* = 10.0 Hz), 6.92 (d, 1H, *J* = 2.0 Hz), 7.04 (d, 2H, *J* = 2.4 Hz), 7.22 (d, 1H, *J* = 2.0 Hz), 7.26 (s, 1H), 7.28 (s, 1H), 7.32 (d, 1H, *J* = 2.4 Hz), 7.41 (d, 1H, *J* = 2.0 Hz), 7.58 (d, 1H, *J* = 2.0 Hz), 7.98 (s, 1H), 8.20 (t, 1H, *J* = 6.4 Hz), 8.35 (t, 1H, *J* = 6.0 Hz); ^13^C NMR (100 MHz, CDCl_3_) *δ* (ppm): 25.0, 25.4, 30.3, 31.0, 31.1, 31.3, 31.5, 31.6, 31.9, 33.9, 34.3, 38.4, 70.9, 72.5, 74.1, 74.5, 122.9, 124.6, 125.8, 126.1, 126.5, 126.6, 126.7, 127.0, 128.0, 128.7, 129.6, 131.2, 131.6, 133.8, 143.0, 143.2, 147.8, 148.4, 149.0, 149.4, 151.8, 153.0, 168.1, 169.0; IR (KBr, cm^−1^) *v* 3369, 3389, 3233, 3194, 3049, 2961, 2868, 1686, 1535, 1485, 1298, 874; MS (m/z): HRMS (ESI) Calcd for C_53_H_70_N_2_NaO_7_ ([M+Na]^+^): 869.5075, found: 869.5082.

**7,13,19,25-tetra*-tert*-butyl-28,30-di-hydroxy-27,29-di-(N-ethyl-aminocarbonylmethoxyl)-2,3-dihomo-3-oxacalix [4]arene (4H):** White solid, m.p. 138.9–141.2°C, yield: 87.2% (Figure S10); ^1^H NMR (400 MHz, CDCl_3_) *δ* (ppm): 1.16 (s, 9H), 1.24 (t, 6H, *J* = 14.6 Hz), 1.25 (brs, 18H), 1.27 (s, 9H), 3.32–3.39 (m, 2H), 3.45 (t, 2H, *J* = 12.8 Hz), 3.53 (q, 2H, *J* = 6.8 Hz), 3.61 (q, 1H, *J* = 6.4 Hz), 4.14 (dd, 2H, *J*_1_ = 18.8 Hz, *J*_2_ = 13.6 Hz), 4.22 (d, 1H, *J* = 10.4 Hz), 4.33 (d, 1H, *J* = 13.2 Hz), 4.38–4.45 (m, 2H), 4.52 (d, 1H, *J* = 15.2 Hz), 4.64 (d, 1H, *J* = 10.0 Hz), 4.72 (dd, 2H, *J*_1_ = 18.0 Hz, *J*_2_ = 2.0 Hz), 4.89 (d, 1H, *J* = 10.0 Hz), 6.89 (d, 1H, *J* = 2.0 Hz), 7.02 (d, 1H, *J* = 1.6 Hz), 7.05 (d, 1H, *J* = 2.0 Hz), 7.17 (dd, 2H, *J*_1_ = 7.6 Hz, *J*_2_ = 2.0 Hz), 7.22 (dd, 2H, *J*_1_ = 10.4 Hz, *J*_2_ = 2.0 Hz), 7.45 (d, 1H, *J* = 2.0 Hz), 7.59 (s, 1H), 8.17 (s, 1H), 8.75 (t, 1H, *J* = 5.2 Hz), 8.93 (t, 1H, *J* = 5.6 Hz); ^13^C NMR (100 MHz, CDCl_3_) *δ* (ppm): 14.1, 14.8, 31.1, 31.2, 31.3, 31.5, 32.2, 33.9, 34.0, 34.2, 34.3, 34.4, 71.0, 72.0, 74.6, 122.3, 124.7, 125.7, 125.9, 126.2, 126.8, 127.2, 127.3, 127.4, 127.7, 128.8, 129.6, 131.5, 132.4, 133.7, 142.9, 143.7, 148.1, 148.6, 148.9, 149.5, 151.5, 153.1, 167.9, 168.3; IR (KBr, cm^−1^) *v* 3350, 3244, 2961, 2870, 1682, 1485, 874; MS (m/z): HRMS (ESI) Calcd for C_53_H_72_N_2_NaO_7_ ([M+Na]^+^): 871.5232, found: 871.5249.

**7,13,19,25-tetra-*tert*-butyl-28,30-di-hydroxy-27,29-di-(N-isopropyl-aminocarbonylmethoxyl)-2,3-dihomo-3-oxa calix[4]arene (4I):** White solid, m.p. 133.3–135.7°C, yield: 76.3% (Figure S11); ^1^H NMR (400 MHz, CDCl_3_) *δ* (ppm): 1.16 (s, 9H), 1.20 (d, 4H, *J* = 6.4 Hz), 1.25 (brs, 18H), 1.29 (s, 9H), 1.31 (d, 8H, *J* = 4.8 Hz), 3.41 (t, 2H, *J* = 14.8 Hz), 3.57 (d, 1H, *J* = 13.6 Hz), 4.11 (d, 1H, *J* = 13.6 Hz), 4.18–4.47 (m, 8H), 4.55 (d, 1H, *J* = 10.4 Hz), 4.77 (dd, 2H, *J*_1_ = 18.8 Hz, *J*_2_ = 15.6 Hz), 4.99 (d, 1H, *J* = 10.4 Hz), 6.85 (s, 1H), 7.03 (d, 2H, *J* = 14.4 Hz), 7.15 (s, 2H), 7.23 (d, 2H, *J* = 4.0 Hz), 7.45 (s, 1H), 7.61 (s, 1H), 7.90 (s, 1H), 8.79 (dd, 2H, *J*_1_ = 19.6 Hz, *J*_2_ = 7.6 Hz); ^13^C NMR (100 MHz, CDCl_3_) *δ* (ppm): 22.0, 22.3, 22.6, 31.1, 31.3, 31.5, 31.6, 33.9, 34.2, 41.4, 41.5, 71.0, 71.8, 74.2, 74.6, 121.9, 123.9, 125.6, 125.8, 126.1, 127.0, 127.4, 127.5, 127.6, 128.5, 129.7, 131.8, 132.4, 133.8, 142.8, 143.5, 147.7, 148.4, 149.0, 150.3, 151.3, 153.8, 167.4, 167.9; IR (KBr, cm^−1^) *v* 3340, 2962, 2870, 1682, 1485, 1460, 874; MS (m/z): HRMS (ESI) Calcd for C_55_H_76_N_2_NaO_7_ ([M+Na]^+^): 899.5545, found: 899.5562.

**7,13,19,25-tetra-*tert*-butyl-28,30-di-hydroxy-27,29-di-(N-isobutyl-aminocarbonylmethoxyl)-2,3-dihomo-3-oxacalix [4]arene (4J)**: White solid, m.p. 128.1–130.8°C, yield: 86.5% (Figure S12); ^1^H NMR (400 MHz, CDCl_3_) *δ* (ppm): 0.90–0.98 (m, 12H), 1.16 (s, 9H), 1.24 (brs, 18H), 1.26 (s, 9H) 1.85 (s, 1H), 1.99 (s, 1H), 2.94–3.04 (m, 2H), 3.38–3.46 (m, 3H), 3.56 (d, 2H, *J* = 13.6 Hz), 4.08 (d, 1H, *J* = 15.2 Hz), 4.21 (d, 2H, *J* = 12.4 Hz), 4.35–4.48 (m, 4H), 4.59 (d, 1H, *J* = 10.4 Hz), 4.75–4.84 (m, 2H), 4.96 (d, 1H, *J* = 10.4 Hz), 6.85 (s, 1H), 7.02 (d, 2H, *J* = 6.0 Hz), 7.14 (d, 2H, *J* = 11.2 Hz), 7.21 (s, 2H), 7.47 (d, 2H, *J* = 14.4 Hz), 8.09 (s, 1H), 8.62 (s, 1H), 8.94 (s, 1H); ^13^C NMR (100 MHz, CDCl_3_) *δ* (ppm): 20.4, 20.5, 28.5, 31.1, 31.3, 31.5, 33.9, 34.2, 34.3, 46.8, 47.0, 71.2, 72.0, 73.9, 74.5, 122.1, 124.2, 125.5, 125.9, 126.0, 126.9, 127.0, 127.2, 127.3, 127.4, 127.6, 128.6, 129.6, 131.7, 132.3, 133.8, 142.8, 143.4, 147.8, 148.5, 148.8, 149.5, 151.5, 153.2, 168.0, 168.6; IR (KBr, cm^−1^) *v* 3350, 3194, 2961, 2870, 1680, 1483, 1364; MS (m/z): HRMS (ESI) Calcd for C_57_H_80_N_2_NaO_7_ ([M+Na]^+^): 927.5858, found: 927.5873.

**7,13,19,25-tetra-*tert*-butyl-28,30-di-hydroxy-27,29-di-(N-butyl-aminocarbonylmethoxyl)-2,3-dihomo-3-oxacalix[4] arene (4K):** White solid, m.p. 119.5–120.8°C, yield: 91.9% (Figure S13); ^1^H NMR (400 MHz, CDCl_3_) *δ* (ppm): 0.84 (t, 3H, *J* = 7.2 Hz), 0.92 (t, 3H, *J* = 7.2 Hz), 1.17 (s, 9H), 1.25 (brs, 18H), 1.28 (s, 9H) 1.32–1.43 (m, 4H), 1.56–1.71 (m, 4H), 3.19–3.31 (m, 2H), 3.44 (t, 2H, *J* = 14.0 Hz), 3.50–3,57 (m, 2H), 3.61–3.66 (m, 1H), 4.11 (d, 1H, *J* = 13.2 Hz), 4.17–4.24 (m, 2H), 4.35–4.42 (m, 3H), 4.49 (d, 1H, *J* = 15.2 Hz), 4.63 (d, 1H, *J* = 10.0 Hz), 4.76 (dd, 2H, *J*_1_ = 18.8 Hz, *J*_2_ = 15.2 Hz), 4.93 (d, 1H, *J* = 10.4 Hz), 6.88 (d, 1H, *J* = 2.0 Hz), 7.04 (dd, 2H, *J*_1_ = 11.2 Hz, *J*_2_ = 1.6 Hz), 7.16 (dd, 2H, *J*_1_ = 8.4 Hz, *J*_2_ = 1.6 Hz), 7.23 (dd, 2H, *J*_1_ = 6.4 Hz, *J*_2_ = 1.6 Hz), 7.46 (d, 1H, *J* = 2.0 Hz), 7.55 (s, 1H), 8.14 (s, 1H), 8.67 (t, 1H, *J* = 5.2 Hz), 8.91 (t, 1H, *J* = 5.2 Hz); ^13^C NMR (100 MHz, CDCl_3_) *δ* (ppm): 13.7, 13.8, 20.2, 31.1, 31.3, 31.5, 34.3, 39.1, 39.3, 71.1, 72.1, 74.0, 74.5, 122.2, 124.4, 125.7, 125.9, 126.0, 126.9, 127.1, 127.2, 127.3, 127.4, 127.6, 128.7, 129.6, 131.6, 132.3, 133.8, 142.8, 143.5, 148.0, 148.5, 148.9, 149.6, 151.5, 153.2, 167.9, 168.3; IR (KBr, cm^−1^) *v* 3350, 3049, 2959, 2868, 1682, 1537, 1298, 874; MS (m/z): HRMS (ESI) Calcd for C_57_H_80_N_2_NaO_7_ ([M+Na]^+^): 927.5858, found: 927.5874.

**7,13,19,25-tetra-*tert*-butyl-28,30-di-hydroxy-27,29-di-(N-pentyl-aminocarbonylmethoxyl)-2,3-dihomo-3-oxacalix[4] arene (4L):** Light yellow solid, m.p. 117.7–119.3°C, yield: 72.4% (Figure S14); ^1^H NMR (400 MHz, CDCl_3_) *δ* (ppm): 0.67 (t, 3H, *J* = 7.2 Hz), 0.82 (t, 3H, *J* = 7.2 Hz), 1.16, 1.24, 1.26 (4s, 36H), 1.29–1.42 (m, 7H), 1.53–1.67 (m, 5H), 3.15–3.25 (m, 2H), 3.43 (t, 2H, *J* = 14.0 Hz), 3.50–3.65 (m, 3H), 4.09 (d, 1H, *J* = 13.6 Hz), 4.17–4.24 (m, 2H), 4.37 (d, 2H, *J* = 14.4 Hz), 4.41 (d, 1H, *J* = 10.4 Hz), 4.47 (d, 1H, *J* = 14.8 Hz), 4.61 (d, 1H, *J* = 10.0 Hz), 4.73–4.81 (m, 2H), 4.93 (d, 1H, *J* = 10.4 Hz), 6.87 (d, 1H, *J* = 2.0 Hz), 7.02 (dd, 2H, *J*_1_ = 11.2 Hz, *J*_2_ = 2.4 Hz), 7.15 (dd, 2H, *J*_1_ = 9.6 Hz, *J*_2_ = 2.0 Hz), 7.22 (q, 2H, *J* = 2.4 Hz), 7.46 (d, 1H, *J* = 2.4 Hz), 7.54 (s, 1H), 8.19 (s, 1H), 8.68 (s, 1H), 8.95 (s, 1H); ^13^C NMR (100 MHz, CDCl_3_) *δ* (ppm): 13.8, 14.0, 22.4, 22.5, 29.3, 31.1, 31.3, 31.5, 33.9, 39.4, 39.6, 71.2, 72.1, 73.9, 74.5, 122.2, 124.4, 125.6, 126.0, 126.9, 127.0, 127.2, 127.3, 127.4, 127.7, 128.7, 129.6, 131.6, 132.3, 133.8, 142.9, 143.5, 147.9, 148.5, 148.9, 149.5, 151.5, 152.2, 167.8, 168.3; IR (KBr, cm^−1^) *v* 3348, 3049, 2959, 2868, 1682, 1599, 1537, 1485, 1445, 874; MS (m/z): HRMS (ESI) Calcd for C_59_H_84_N_2_NaO_7_ ([M+Na]^+^): 955.6176, found: 955.6174.

**7,13,19,25-tetra-*tert*-butyl-28,30-di-hydroxy-27,29-di-(N-clopentyl-aminocarbonylmethoxyl)-2,3-dihomo-3-oxacalix[4]arene (4M):** White solid, m.p. 145.9–148.2°C, yield: 83.61% (Figure S15); ^1^H NMR (400 MHz, DMSO-*d*_6_) *δ* (ppm): 1.12, 1.22, 1.25, 1.26 (4s, 36H), 1.51–1.72 (m, 12H), 1.87–1.96 (m, 4H), 3.49 (q, 2H, *J* = 6.4 Hz), 3.58 (d, 1H, *J* = 13.2 Hz), 4.19–4.26 (m, 5H), 4.34–4.42 (m, 3H), 4.47 (d, 1H, *J* = 14.8 Hz), 4.57 (d, 1H, *J* = 14.8 Hz), 4.65–4.72 (m, 2H), 4.93 (d, 1H, *J* = 10.0 Hz), 6.95, 7.05, 7.10 (3s, 3H), 7.29 (d, 2H, *J* = 8.4 Hz), 7.35 (s, 1H), 7.46 (s, 1H), 7.60 (s, 2H), 8.14 (s, 1H), 8.32 (d, 1H, *J* = 7.6 Hz), 8.47 (d, 1H, *J* = 7.2 Hz); ^13^C NMR (100 MHz, DMSO-*d*_6_) *δ* (ppm): 24.1, 31.3, 31.5, 31.8,32.4, 32.8, 33.0, 34.1, 34.4, 50.7, 50.8, 71.5, 74.1, 74.5, 122.3, 124.1, 126.0, 126.1, 126.3, 127.1, 127.3, 128.2, 129.4, 132.3, 132.9, 134.1, 142.1, 142.5, 147.2, 147.4, 149.5, 151.8, 153.9, 167.6, 168.1; IR (KBr, cm^−1^) *v* 3342, 3049, 2961, 2870, 1682, 1529, 1483; MS (m/z): HRMS (ESI) Calcd for C_59_H_80_N_2_NaO_7_ ([M+Na]^+^): 951.5858, found: 951.5862.

**7,13,19,25-tetra-*tert*-butyl-28,30-di-hydroxy-27,29-di-(N-benzyl-aminocarbonylmethoxyl)-2,3-dihomo-3-oxacalix[4] arene (4N):** m.p. 183.7–186.4°C, yield: 72.0% (Figure S16); ^1^H NMR (400 MHz, CDCl_3_) *δ* (ppm): 1.12, 1.20, 1.25 (4s, 36H), 3.32 (t, 2H, *J* = 12.8 Hz), 3.42 (d, 1H, *J* = 13.6 Hz), 3.90 (d, 1H, *J* = 13.6 Hz), 4.03 (dd, 2H, *J*_1_ = 13.2 Hz, *J*_2_ = 4.4 Hz), 4.15 (dd, 2H, *J*_1_ = 15.6 Hz, *J*_2_ = 10.0 Hz), 4.26–4.32 (m, 2H), 4.41–4.57 (m, 4H), 4.64 (s, 1H), 4.67 (d, 1H, *J* = 4.0 Hz), 4.74 (dd, 1H, *J*_1_ = 14.4 Hz, *J*_2_ = 6.4 Hz), 4.83 (dd, 1H, *J*_1_ = 14.4 Hz, *J*_2_ = 6.4 Hz), 6.79 (d, 1H, *J* = 2.4 Hz), 6.92 (d, 1H, *J* = 2.4 Hz), 6.98 (d, 1H, *J* = 2.8 Hz), 7.04–7.11 (m, 6H), 7.13 (s, 1H), 7.15 (d, 1H, *J* = 2.4 Hz), 7.19–7.24 (m, 3H), 7.27 (d, 1H, *J* = 1.6 Hz), 7.28 (s, 1H), 7.38 (dd, 3H, *J*_1_ = 5.8 Hz, *J*_2_ = 2.0 Hz), 7.74 (s, 1H), 8.93 (t, 1H, *J* = 5.6 Hz), 9.25 (t, 1H, *J* = 5.6 Hz); ^13^C NMR (100 MHz, CDCl_3_) *δ* (ppm): 31.1, 31.2, 31.5, 31.6, 33.9, 34.2, 43.6, 43.8, 72.2, 73.8, 122.3, 124.5, 125.5, 125.7, 126.1, 126.6, 126.7, 126.9, 127.0, 127.2, 127.3, 127.5, 128.4, 128.5, 128.7, 129.4, 131.4, 132.1, 133.7, 137.4, 138.0, 142.6, 143.2, 148.0, 148.3, 148.7, 149.6, 151.3, 153.0, 168.1, 168.5; IR (KBr, cm^−1^) *v* 3448, 3389, 2955, 2870, 1670, 1485, 1439; MS (m/z): HRMS (ESI) Calcd for C_63_H_77_N_2_O_7_ ([M+H]^+^): 973.5725, found: 973.5716.

**7,13,19,25-tetra-*tert*-butyl-28,30-di-hydroxy-27,29-di-(N-phenethyl-aminocarbonylmethoxyl)-2,3-dihomo-3-oxacalix [4]arene (4O):** White solid, m.p. 114.6–115.9°C, yield: 67.1% (Figure S17); ^1^H NMR (400 MHz, CDCl_3_) *δ* (ppm): 1.15, 1.24, 1.27, 1.28 (4s, 36H), 2.88–3.04 (m, 4H), 3.27 (d, 1H, *J* = 13.2 Hz), 3.32 (d, 1H, *J* = 12.8 Hz), 3.46–3.58 (m, 3H), 3.84–3.98 (m, 4H), 4.14 (d, 1H, *J* = 13.2 Hz), 4.18 (d, 1H, *J* = 10.4 Hz), 4.32 (d, 1H, *J* = 10.0 Hz), 4.37 (d, 1H, *J* = 14.8 Hz), 4.43 (d, 1H, *J* = 8.8 Hz), 4.46 (d, 1H, *J* = 3.6 Hz), 4.73 (d, 1H, *J* = 7.6 Hz), 4.76 (d, 1H, *J* = 8.0 Hz), 4.88 (d, 1H, *J* = 10.4 Hz), 6.85 (d, 1H, *J* = 1.6 Hz), 6.94–6.99 (m, 2H), 7.03–7.22 (m, 14H), 7.43 (d, 1H, *J* = 2.0 Hz), 7.48 (s, 1H), 8.05 (s, 1H), 8.79 (t, 1H, *J* = 5.2 Hz), 9.01 (t, 1H, *J* = 5.6 Hz); ^13^C NMR (100 MHz, CDCl_3_) *δ* (ppm): 31.1, 31.3, 31.6, 32.4, 33.9, 34.2, 35.1, 35.5, 40.6, 40.6, 71.1, 72.0, 73.9, 74.4, 122.2, 124.2, 125.5, 125.9, 126.0, 126.1, 126.2, 126.8, 127.0, 127.2, 127.5, 127.7, 128.2, 128.4, 128.5, 128.7, 129.6, 131.6, 132.4, 133.8, 138.6, 138.9, 142.8, 143.5, 147.9, 148.5, 148.8, 149.3, 151.4, 153.0, 168.0, 168.5; IR (KBr, cm^−1^) *v* 3346, 2961, 2905, 2866, 1682, 1537, 1483, 1445, 1364; MS (m/z): HRMS (ESI) Calcd for C_65_H_80_N_2_NaO_7_ ([M+Na]^+^): 1023.5860, found: 1023.5870.

**7,13,19,25-tetra-*tert*-butyl-28,30-di-hydroxy-27,29-di-(N-(3,4-dimethoxyphenethyl)-aminocarbonylmethoxyl)-2,3-dihomo-3-oxacalix [4]arene (4P):** White solid, m.p. 117.4–120.1°C, yield: 76.4% (Figure S18); ^1^H NMR (400 MHz, CDCl_3_) *δ* (ppm): 1.14, 1.23, 1.24, 1.25 (4s, 36H), 2.86–2.95 (m, 4H), 3.34 (d, 2H, *J* = 12.0 Hz), 3.46 (d, 1H, *J* = 13.6 Hz), 3.50–3.60 (m, 2H), 3.72–3.82 (m, 12H), 3.90–4.07 (m, 5H), 4.15 (d, 1H, *J* = 10.4 Hz), 4.28 (d, 1H, *J* = 9.2 Hz), 4.35–4.46 (m, 3H), 4.68 (t, 2H, *J* = 14.4 Hz), 4.81 (d, 1H, *J* = 10.0 Hz), 6.49 (d, 1H, *J* = 8.0 Hz), 6.58 (d, 1H, *J* = 7.2 Hz), 6.64 (d, 1H, *J* = 7.6 Hz), 6.73 (s, 3H), 6,84 (s, 1H), 7.00 (d, 2H, *J* = 12.8 Hz), 7.15 (t, 4H, *J* = 17.6 Hz), 7.41 (s, 1H), 7.51 (s, 1H), 7.94 (s, 1H), 8.76 (s, 1H), 8.90 (s, 1H); ^13^C NMR (100 MHz, CDCl_3_) *δ* (ppm): 30.9, 31.1, 31.2, 31.5, 33.9, 34.2, 34.3, 34.8, 35.1, 40.7, 55.8, 55.9, 70.9, 71.8, 73.9, 111.2, 111.3, 111.8, 112.0, 120.6, 120.7, 122.3, 127.1, 127.2, 127.4, 128.6, 131.3, 131.6, 133.8, 142.8, 143.3, 147.4, 148.0, 148.6, 148.9, 149.1, 151.4, 152.8, 168.0, 168.5; IR (KBr, cm^−1^) *v* 3348, 3049, 2957, 2868, 1682, 1516, 1485, 1362, 874.

**7,13,19,25-tetra-*tert*-butyl-28,30-di-hydroxy-27-(N-clopentyl-aminocarbonylmethoxyl)-29-(N-(2-hydroxyethyl)-aminocarbonylmethoxyl)-2,3-dihomo-3-oxacalix[4]arene (4Q)**: White solid, yield: 82.5%, m.p. 138.3–140.5°C (Figure S19); ^1^H NMR (400 MHz, CDCl_3_) *δ* (ppm): 1.14, 1.19, 1.24, 1.26 (4s, 36H), 1.59–1.80 (m, 8H), 3.32–3.52 (m, 4H), 3.56 (d, 1H, *J* = 13.8 Hz), 3.67–3.79 (m, 4H), 4.05–4.22 (m. 4H), 4.31–4.45 (m, 5H), 4.52–4.60 (m, 2H), 4.69–4.91 (m, 3H), 4.99 (d, 1H, *J* = 10.8 Hz), 6.86–7.13 (m, 6H), 7.22 (d, 2H, *J* = 8.0 Hz), 7.37 (d, 1H, *J* = 5.6 Hz), 7.86 (d, 1H, *J* = 14.0 Hz), 8.70–9.01 (m, 2H); ^13^C NMR (100 MHz, CDCl_3_) *δ* (ppm): 23.9, 24.1, 31.0, 31.2, 31.4, 31.6, 32.0, 32.4, 32.9, 33.3, 33.9, 42.6, 51.2, 62.0, 71.5, 72.0, 122.3, 124.2, 125.5, 125.8, 126.1, 126.9, 127.1, 127.3, 127.5, 127.7, 128.4, 129.4, 131.7, 132.2, 133.6, 143.0, 143.5, 147.7, 148.0, 148.5, 148.9, 149.6, 150.0, 151.3, 153.6, 168.8, 169.0; IR (KBr, cm^−1^) ν 3350, 2961, 2870, 1674, 1485, 1298, 1196, 1070; MS (m/z): HRMS (ESI) Calcd for C_56_H_76_N_2_NaO_8_ ([M+Na]^+^): 927.5499, found: 927.5486.

**7,13,19,25-tetra-tert-butyl-28,30-di-hydroxy-27-(N-phenethyl-aminocarbonylmethoxyl)-29-(N-(2-hydroxyethyl)-aminocarbonylmethoxyl)-2,3-dihomo-3-oxacalix[4]arene (4R):** White solid, m.p. 120.7–123.3°C, yield: 81.6% (Figure S20); ^1^H NMR (400 MHz, CDCl_3_) *δ* (ppm): 1.00 (s, 3H), 1.14 (s, 6H), 1.18 (s, 6H), 1.21 (s, 3H), 1.24 (s, 6H), 1.26 (s, 9H), 1.28 (s, 3H), 2.87–3.04 (m, 3H), 3.26–3.38 (m, 1H), 3.42–3.53 (m, 3H), 3.54–3.64 (m, 2H), 3.70–3.78 (m, 2H), 4.00–4.11 (m, 2H), 4.23 (dd, 2H, *J*_1_ = 12.0 Hz, *J*_2_ = 7.8 Hz), 4.30 (d, 1H, *J* = 10.4 Hz), 4.38–4.43 (m, 2H), 4.50 (dd, 2H, *J*_1_ = 14.8 Hz, *J*_2_ = 9.6 Hz), 4.61 (dd, 1H, *J* = 16.4, 6.6 Hz), 4.71 (dd, 1H, *J*_1_ = 15.4 Hz, *J*_2_ = 5.8 Hz), 4.83 (dd, 1H, *J*_1_ = 10.2 Hz, *J*_2_ = 5.6 Hz), 6.79–6.89 (m, 1H), 6.94–7.03 (m, 3H), 7.03–7.11 (m, 2H), 7.11–7.15 (m, 3H), 7.16 (d, 3H, *J* = 4.4 Hz), 7.22 (s, 1H), 7.34–7.41 (m, 2H), 7.94 (s, 1H), 8.69–8.80 (m, 1H), 8.90 (t, 1H, *J* = 5.6 Hz); ^13^C NMR (100 MHz, CDCl_3_) *δ* (ppm): 30.9, 31.1, 31.2, 31.5, 31.6, 32.2, 33.9, 34.2, 35.3, 35.5, 40.6, 40.7, 42.5, 62.0, 62.4, 70.9, 71.8, 73.9, 74.3, 122.3, 124.5, 125.6, 125.9, 126.1, 126.2, 126.6, 127.2, 127.3, 127.7, 128.3, 128.4, 128.5, 128.6, 128.7, 129.4, 131.6, 132.2, 133.5, 133.9, 138.7, 138.8, 143.0, 143.6, 148.0, 148.5, 148.7, 148.8, 149.4, 151.3, 152.7, 168.7, 168.9, 169.3; IR (KBr, cm^−1^) ν 3366, 2961, 2868, 1670, 1541, 1485, 1200, 1072, 874, 579; MS (m/z): HRMS (ESI) Calcd for C_59_H_76_N_2_NaO_8_ ([M+Na]^+^): 963.5494, found: 963.5491.

**7,13,19,25-tetra-tert-butyl-28,30-di-hydroxy-27-(N,N-bis(2-hydroxyethyl)-aminocarbonylmethoxyl)-29-(N-(2-hydroxyethyl)-aminocarbonylmethoxyl)-2,3-dihomo-3-oxacalix[4]arene (4S):** White solid, m.p. 144.4–146.2°C, yield: 85.9% (Figure S21); ^1^H NMR (400 MHz, CDCl_3_) *δ* (ppm): 1.07, 1.23, 1.25, 1.26 (4s, 36H), 3.31–3.58 (m, 8H), 3.68–3.89 (m, 8H), 4.00 (s, 2H), 4.22 (d, 1H, *J* = 13.2 Hz), 4.36 (d, 1H, *J* = 13.2 Hz), 4.58 (d, 1H, *J* = 14.8 Hz), 4.91–5.02 (m, 4H), 6.81 (s, 1H), 6.89 (s, 1H),7.02 (s, 2H), 7.10 (s, 1H), 7.16 (d, 2H, *J* = 6.8 Hz), 7.43 (s, 1H), 7.83 (s, 1H), 8.99 (s, 1H), ^13^C NMR (100 MHz, CDCl_3_) *δ* (ppm): 30.6, 31.0, 31.1, 31.6, 31.7, 33.9, 34.1, 34.3, 42.4, 49.9, 51.3, 59.9, 60.0, 62.0, 71.2, 72.2, 72.7, 74.0, 122.8, 124.2, 125.1, 125.5, 15.8, 125.9, 126.6, 128.2, 129.0, 129.2, 129.4, 132.0, 132.2, 134.4, 142.4, 143.0, 146.8, 148.0, 148.9, 150.9, 151.7, 153.7, 170.3, 170.4; IR (KBr, cm^−1^) ν 3398, 2961, 2870, 1485, 1364, 1202, 1068, 874; MS (m/z): HRMS (ESI) Calcd for C_55_H_76_N_2_NaO_10_ ([M+Na]^+^): 947.5398, found: 947.5408.

### Crystallography

The single crystal of dihomooxacalix[4]arene amide 4L and 4N were obtained in ethanol and their single crystal structures were determined on a Bruker Smart Apex single crystal diffractometer. The data were processed with HKL2000. The structure was solved by direct methods of SHELX86 and subsequent Fourier-difference synthesis and refined by full-matrix least-squares on F^2^ with SHELXS-97 (Sheldrick, 1997). No absorption correction was done. All non-hydrogen atoms were refined with anisotropic displacement parameters.

### Cell Culture

A549 cells (human lung carcinoma cells), MCF-7 cells (human breast cancer cells), HeLa cells (human cervical cancer cells), HepG2 cells (human hepatocellular carcinoma cells), and HUVEC cells (human umbilical vein endothelial cells) were kindly provided by WeiFang Caleb Pharmaceuticals, Inc. A549 cells were cultured in Ham's F12K medium containing 10% fetal bovine serum, 2 mM L-glutamine, and 1.5 g/L sodium bicarbonate; MCF-7 cells were cultured on cell culture flask using RPMI 1640 medium with 2 mM L-glutamine adjusted to contain 1.5 g/L sodium bicarbonate, 4.5 g/L glucose, 10 mM HEPES, 1.0 mM sodium pyruvate, and 10% fetal bovine serum. Hela cells were cultured on Cell culture flask using 2 mM L-glutamine adjusted to contain 1.5 g/L sodium bicarbonate, 4.5 g/L glucose, 10 mM HEPES, and 1.0 mM sodium pyruvate in RPMI 1640 medium supplemented with 0.5 mg/ml G418 and 10% fetal bovine serum. HepG2 cells were cultured in minimum essential medium (Eagle) with 2 mM L-glutamine, Earle's BSS, 1.5 g/L sodium bicarbonate, 0.1 mM non-essential amino acids, 1.0 mM sodium pyruvate, and 10% fetal bovine serum. HUVEC cells were cultured in M199 medium containing 2 mM L-glutamine adjusted to contain 1.5 g/L sodium bicarbonate, 4.5 g/L glucose, 10 mM HEPES, 1.0 mM sodium pyruvate, ECGS, and 10% fetal bovine serum. All cells were cultured at 37°C in 5% CO_2_.

### Cytotoxicity Assay

The cell proliferation of adherent cells was determined by sulforhodamine B assay (SRB) (Quan et al., 2009; Li et al., 2017). All cells were cultured in culture medium containing 10% fetal bovine serum and have been in the logarithmic growth phase. All cell types were seeded in a 96-well culture plate at a concentration of 1–5 × 10^4^ cells per well at 37°C in a 5% CO_2_ incubator for 24 h. The cells were then exposed to seven drug concentrations of dihomooxacalix[4]arene derivatives for 72 h with each concentration located in three wells. Then, the cells were fixed with trichloroacetic acid (TCA). After washing, SRB working solution was added to the cells to clean them. SRB combined with protein was dissolved in Tris base. OD values were measured for each well with a SPECTRA max 190 Cell microplate reader under 540 nm wavelength. According to the OD value, the cell growth inhibition rate was calculated.

### Flow Cytometry

#### Flow Cytometry for Cell Cycle Analysis

For flow cytometric analysis of DNA content, MCF-7 cells in exponential growth were treated with compound for 48 h. The cells treated with compound were collected, washed twice with PBS, and then fixed with 75% alcohol overnight. Then, the cells were washed with PBS and resuspended in 100 μl of PBS, 200 mg/ml RNase was added for 30 min to eliminate the interference of RNA, and 20 ml/L propidium iodide (PI; Sigma) was added for 30 min. Then, the cells were washed, and the DNA content was detected by a flow cytometer (BD Accuri C6).

#### Flow Cytometry for Cell Apoptosis Analysis

MCF-7 cells (5 × 10^5^ cells/ml) were seeded in six-well plates and treated with compounds at different concentrations for 48 h. The cells were then harvested by trypsinization and washed twice with cold PBS. After centrifugation and removal of the supernatants, cells were resuspended in 400 μl of 1 × binding buffer, which was then added to 5 μl of annexin V-FITC and incubated at room temperature for 15 min. After adding 10 μl of PI, the cells were incubated at room temperature for another 15 min in the dark. The stained cells were analyzed by a flow cytometer (BD Accuri C6).

### Statistical Analysis

Data were calculated using the non-parametric variance. Comparisons among groups were statistically analyzed. *P* < 0.05 was considered statistically significant. Statistical analyses were conducted using SPSS 16.0 (SPSS, Chicago, IL, USA).

## Results and Discussion

### Synthesis Investigation

**Scheme 1:**
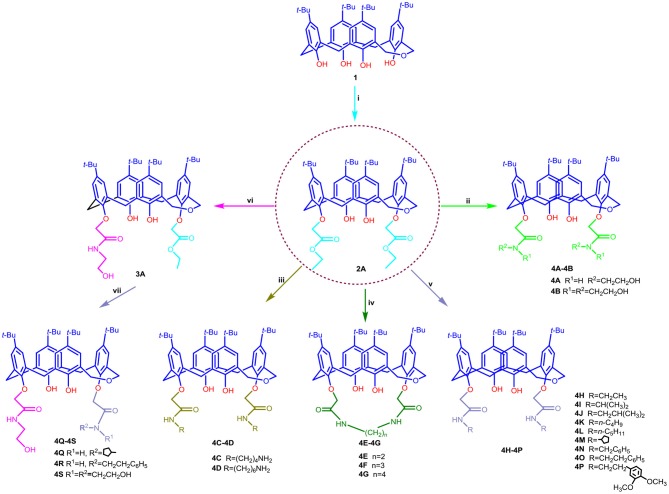
Synthetic routes for dihomooxacalix[4]arene amide derivatives **4A–4S**. Reagents and conditions: (i) BrCH_2_COOC_2_H_5_, K_2_CO_3_, acetone, reflux; (ii) NH_2_CH_2_CH_2_OH or NH(CH_2_CH_2_OH)_2_, ethanol/toluene, reflux; (iii) NH_2_(CH_2_)_n_NH_2_ (*n* = 4, 6), methanol, rt; (iv) NH_2_(CH_2_)_n_NH_2_(*n* = 2, 3, 4), ethanol, reflux; (v) NH_2_R, ethanol; (vi) NH_2_CH_2_CH_2_OH, dichloromethane; (vii) NH_2_R, rt.

The routes used for the synthesis of the target compounds **4A−4S** are outlined in Scheme 1.

As outlined in Scheme 1, all the targets **4A−4S** consist of 1,3-alternate dihomooxacalix[4]arene conformers. As a first step in our development of dihomooxacalix[4] arene amide derivatives, 1,3-di-ethoxycarbonyl substituted dihomooxacalix [4]arene intermediate **2A** initially synthesized as the key intermediate from known *tert*-butyl dihomooxacalix[4]arene **1** by esterification with bromoethyl acetate in the presence of potassium carbonate in refluxing acetone. Subsequently, refluxing ester **2A** with excess ethanolamine or diethanolamine in the mixed ethanol/toluene readily afforded the desired amide derivative **4A−4B** in good yield of 87.2 and 86.7%, respectively. The derivatives **4C−4D** were obtained by reacting ester **2A** with an excess amount of diamines in methanol at low temperature (initially stirring in ice bath, and then up to room temperature) and the satisfying yields was 63.3 to 96.2%. Interestingly, on replacement of methanol with refluxing ethanol, but under otherwise similar conditions, the two amino groups on the diamine were found to react with ester **2A** simultaneously to give amino-bridging products **4E−4G**. To investigate the effect on affinity of different linker groups attached to the amino and dihomooxacalix[4]arene components, compounds **4H−4P** were successfully accomplished by ester **2A** with aliphatic primary amine by method similar to that for compounds **4C−4D**. However, secondary amine was not ideal for the same condition in that of steric hindrance. In addition, treatment of **2A** with ethanolamine in dichloromethane resulted in the partial aminolysis, leading to the generation of mono-N-(2-hydroxyethyl)-aminocarbonyl dihomooxacalix[4]arene compound **3A**, which was further reacted with aliphatic primary amine in methanol to yield the amide derivatives **4Q−4S**. Separation of the reaction products into the pure compounds **4A−4S** was achieved by column chromatography. All the synthesized compounds were further characterized by IR, ^1^H NMR, ^13^C NMR, and HR-MS spectrometry.

### Single Crystal Structure Analysis

**Figure 3:**
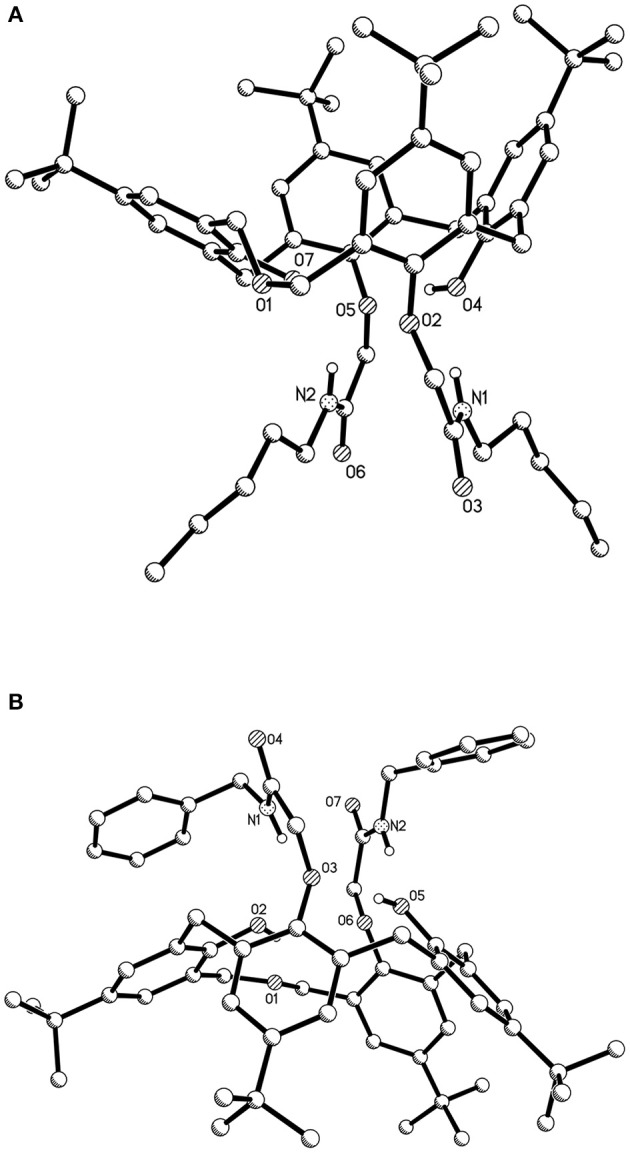
Single crystal structures of dihomooxacalix[4]arene-based compounds (hydrogen atoms on the skeletons were omitted for clarity) **(A)** amide derivative **4L**; **(B)** amide derivative **4N**.

The single crystal structures of dihomooxacalix[4]arene amide derivatives **4L** and **4N** are shown in Figure 3. The crystal data, data collection, and structure refinements are summarized in Table S1.

As shown in Figure 3A, the conformation of dihomooxacalix[4]arene derivative **4L** crystallizes in the monoclinic space group of P 21/c. In molecules, the two N-pentyl-aminocarbonylmethoxyl groups (27, 29-positions) at the lower rim of the rings exist in 1,3-distal position. The four phenolic rings remained standing on same side of the mean plane defined by the four phenolic oxygen atoms O2, O4, O5, and O7. One of the aromatic rings, bearing O7, is partly parallel to the mean plane [dihedral angle: 27.013° (148)]. The remaining three aromatic rings, bearing O2, O4, O5, respectively, are 70.627° (40), 66.852° (38), and 62.138° (42). The 27, 29-di-(N-benzyl-aminocarbonylmethoxyl) dihomooxacalix[4]arene amide derivatives **4N** (Figure 3B) possessing the cone conformation shows some interesting similarities with **4L**. It crystallizes the monoclinic space group of C 2/c. The two benzyl groups are located at slightly longer distances but on the same side of the mean plane defined by O2, O3, O5, and O6 atoms. The dihedral angles of the four aromatic rings are 20.904° (71), 78.442° (69), 52.4762° (57), and 54.069° (59).

### Compounds 4A−4S Reduce the Viability of Cancer Cells

The cell-based assay can provide a useful tool to screen the preliminary specificity (Zhou et al., 2006). From this point, the synthesized compounds **4A−4S** were evaluated for antitumor activity against A549 cells, MCF-7 cells, HeLa cells, and HepG2 cells, as well as HUVEC cells using the SRB method. For initial screening, the single concentration inhibition of compounds **4A−4S** on cell viability at a concentration of 10 μM was assessed 72 h after treatment in comparison with those of reference compound **CLX-4** and the positive control (paclitaxel). The results are summarized in Table 1.

**Table 1:** Single concentration inhibition of dihomooxacalix[4]arene derivatives **4A–4S** on cell viability.

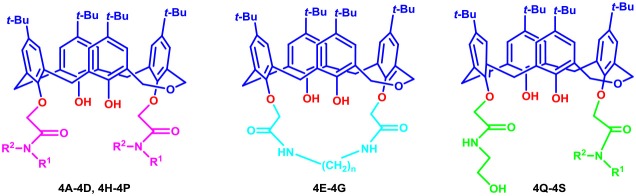							
**Compd**.	**R**^**1**^**/n**	**R**^**2**^	**% inhibition at 10 μM**				
			**A549**	**MCF-7**	**HeLa**	**HepG2**	**HUVEC**
**4A**	**H**	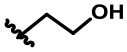	100	100	100	86	41
**4B**	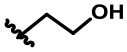	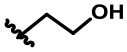	66	73	100	39	23
**4C**	**H**	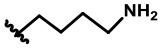	42	78	96	64	55
**4D**	**H**	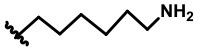	99	99	100	72	90
**4E**	**2**	–	60	50	46	21	26
**4F**	**3**	–	63	46	59	9	42
**4G**	**4**	–	21	34	40	16	34
**4H**	**H**	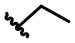	31	37	41	31	6
**4I**	**H**	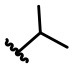	45	17	44	9	16
**4J**	**H**	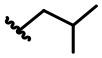	54	40	35	10	0
**4K**	**H**	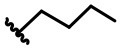	65	41	42	6	1
**4L**	**H**	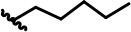	66	41	49	18	12
**4M**	**H**	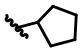	66	29	52	12	14
**4N**	**H**	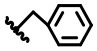	64	43	49	10	21
**4O**	**H**	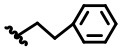	63	45	60	9	35
**4P**	**H**	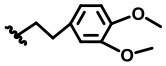	22	0	37	11	0
**4Q**	**H**	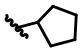	34	0	49	23	18
**4R**	**H**	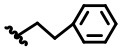	2	0	4	−1	85
**4S**	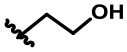	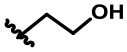	89	96	97	94	21
**CLX-4**	–	–	93	99	92	94	16
**Paclitaxel**	–	–	IC_50_ = 0.59 nM	IC_50_ = 0.8 nM	IC_50_ = 2.7 nM	IC_50_ = 5.0 nM	IC_50_ = 1.0 nM

As shown in Table 1, dihomooxacalix[4] arene derivative **4A**, which still maintains the original N-(2-hydroxyethyl) aminocarbonyl group of precursor **CLX-4**, is the most effective at inducing cytotoxicity in all the four tumor cell types tested with the single concentration inhibition on the four tumor cells ranging from 86 to 100%. The potency is equivalent to that of **CLX-4**. It allows us to conclude that the replacement of calix[4]arene bone with the dihomooxacalix[4]arene scaffold is successful. Encouraged by it, the modification of 2-hydroxyethyl group adjacent to the amido unit of the benzene ring is considered. Thus, a range of substituents, such as alkyl groups with straight or branched chains, alkyl benzyl, and alkyl amino substitution are introduced to the R2 position linked on the amido unit to afford 12 amide derivatives, including **4B-4D**, **4H−4P**. It is revealed that the majority of the above 12 amide derivatives demonstrate a good selectivity profile on A549 cell lines, but display the weak anti-tumor effects on hepG2 cell lines. In this series, derivative **4C** has the selective effect on HeLa cells. In a contrast, compound **4D** with much longer alkyl chain exerts the markable inhibition on all the tested tumor cells. In addition, derivatives with hydroxyethyl or alkyl amino group, e.g., **4A−4D** are particularly more effective than those of bearing alkyl unit with low polarity and hydrophilicity (**4H−4K**) or containing alkyl benzyl group with big steric hindrance (**4P**). Therefore, the inhibition efficiency of those derivatives is significantly involved in the hydrophilicity, steric hindrance, hydrogen bonding, and polarity of the R2 functional groups.

Moreover, the toxicity of those derivatives on HUVEC cells was also investigated. The result indicates that within a reasonable range, the single concentration inhibition of **4A** is 41%, though slightly higher than 18% of **CLX-4**. However, either **4C** or **4D** has much toxicity on HUVEC cell lines with inhibition of 55 and 90%, respectively. This may be due to the influence of the bare primary amino group on the side of alkyl chain. In this way, the further structural optimization was carried and the bridged derivatives **4E−4G** were afforded. As we expected, the toxicity of **4E−4G** on HUVEC cell lines is decreased with the single concentration inhibition degree varying from 16 to 42%. Meanwhile their inhibitory activity is less potent than those of **4C** and **4D**. Thus, N-2-hydroxyethyl attached to amido unit is probably vital to the activity of those compounds. To verify our assumption, one of the N-2-hydroxyethyl groups is replaced by cyclopentyl, phenethyl, or N,N-di-2-hydroxyethyl substituent to give **4Q−4S**.

In this series, compound **4Q** indicated no effect on MCF-7 cell line and indicated about two - to threefold decrease in potency of three other tumor cell lines, compared with those of **CLX-4**. Compound **4R** exerted no viability on the tested tumor cells, but had serious toxicity on HUVEC cell lines. As a contrast, compound **4S** remains a similar antitumor level activity to **CLX-4**, which is essentially in agreement with that of **4A**. The absence of significant activity in **4Q** and **4R** proved that N-2-hydroxyethyl substituent linked on the amido unit is suggested to have a significant impact on the inhibition activity.

**Figure 4:**
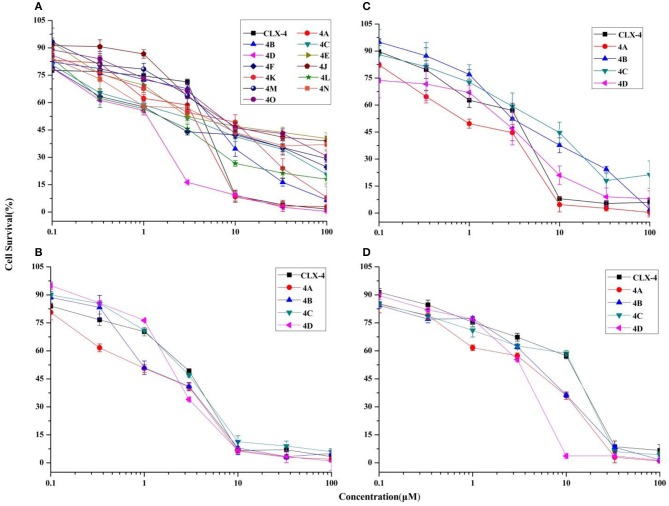
Cytotoxicity effects of dihomooxacalix[4]arene-based compounds. Cell viability of cells **(A)** A549 cells, **(B)** HeLa cells, **(C)** MCF-7 cells, and **(D)** HepG2 cells were measured in the presence of various concentration of dihomooxacalix[4]arene amide derivatives and CLX-4.

Based on the results of initial screening, the determined IC_50_ values of potent dihomooxacalix[4]arene amides for tumor cell viability are measured and described in Table 2. Dose–response curves are shown in Figure 4.

**Table 2:** IC_50_ values of dihomooxacalix[4]arene-based compounds on tumor cells viability.

**Compd**.	**IC**_****50****_ **(μM)**			
	**A549**	**MCF-7**	**HeLa**	**HepG2**
**4A**	2.0 ± 0.5	1.0 ± 0.1	0.8 ± 0.2	2.7 ± 0.4
**4B**	3.9 ± 0.5	4.4 ± 0.7	1.1 ±0.5	3.8 ± 0.3
**4C**	3.3 ± 0.7	5.4 ± 2.6	2.3 ± 0.1	5.0 ± 0.8
**4D**	0.7 ± 0.1	1.8 ± 0.7	1.9 ± 0.1	2.6 ± 0.2
**4E**	12.4 ± 2.2	–	–	–
**4F**	2.9 ± 0.7	–	–	–
**4J**	16.9 ± 3.2	–	–	–
**4K**	4.5 ± 1.4	–	–	–
**4L**	1.7 ± 0.4	–	–	–
**4M**	10.1 ± 1.8	–	–	–
**4N**	6.8 ± 0.5	–	–	–
**4O**	12.6 ± 2.0	–	–	–
**CLX-4**	2.8 ± 0.3	2.1 ± 0.4	1.9 ± 0.2	6.6 ± 0.8

“*N.T.” means no tested*.

Our data in Table 2 shows that almost all dihomooxacalix[4]arene amides exert satisfying potency on A549 cell lines. Compounds **4A−4D** exerted potent growth inhibition against various human cancer cells with IC_50_ values ranging narrowly from 0.6 to 8.0 μM. In particular, **4A** demonstrates the greatest cytotoxic effect against cervical cancer (HeLa) cell line with the IC_50_ of 0.8 ± 0.2 μM, which is at the level of submicromolar concentration. Overall, **4A** and **4D** show enhanced potency over the cytotoxicity profiles (2- to 4-fold) of **CLX-4**, suggesting that our structural optimization is successful.

### Flow Cytometry for MCF-7 Cell Cycle and Apoptosis Analysis

**Figure 5:**
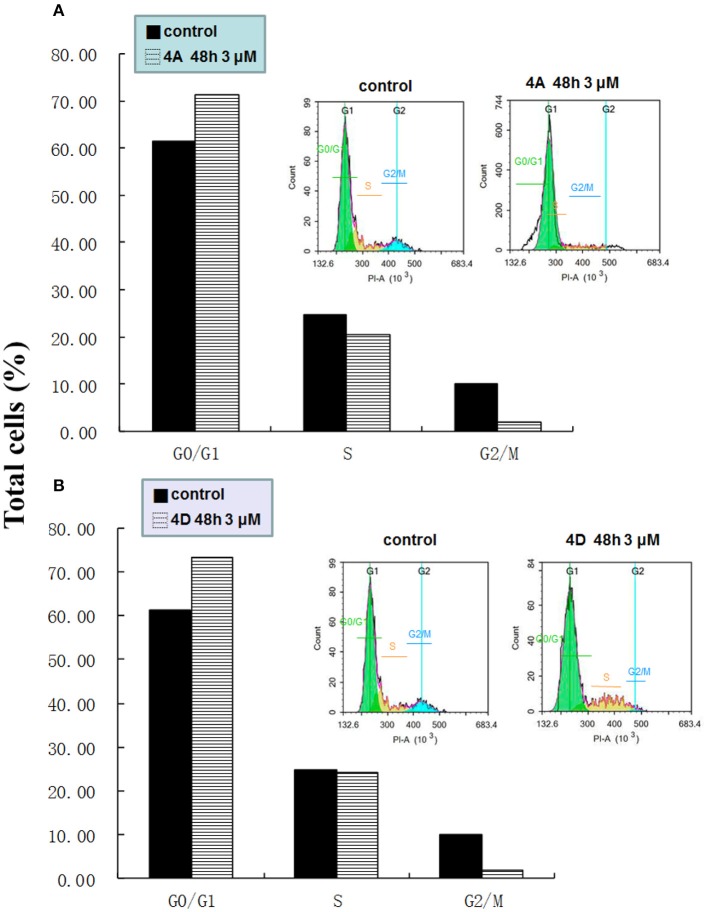
Effect of **4A** and **4D** on the cell cycle of MCF-7 cells. Flow cytometric analyses of MCF-7 cells show that **4A (A)**, **4D (B)** treatment induces G0/G1 arrest in MCF-7.

Cancer progression has been suggested to include the loss of cell cycle checkpoint controls that regulate passage through the cell cycle (Pelizzarorocha et al., 2013; Ji et al., 2017). According to the literatures, Dings and co-workers (Dings et al., 2013) have revealed that calixarene amine derivative **PTX013** had good cytotoxicity to cancer cells and blockades the cell cycle of SQ20B at G0/G1 phase. In addition, Pelizzarorocha et al. (2013) found that *tert*-butyl calix[6]arenes can cause cell cycle arrest in G0/G1 phase by down-regulating key proteins, such as PIM1, CDK2, and CDK4. To have an insight into the underlying mechanism of cytotoxic effects *in vitro*, the key indicators including the cell cycle and apoptosis were observed by the flow cytometry analysis. We first tested if treatment with **4A** or **4D** affects the cell cycle of MCF-7 cells. The results are shown in Figure 5.

As exemplified with MCF-7 cells in Figure 5A, the percentage of non-treated cells was 61.36 ± 3.65% in G0/G1 phase, 24.69 ± 6.30% in S, and 10.06 ± 0.91 in G2/M phase, whereas after the treatment with **4A** at a low concentration (3 μM) for 48 h, MCF-7 cells led to an accumulation number of cells in G0/G1 phase of the cell cycle and the percentage was increased to 71.39 ± 11.21%, whereas it correspondingly reduced to 20.68 ± 13.12% and 1.87 ± 2.45%, respectively, in S and G2/M phase. A similar profile in the cells treated with 3 μM **4D** was supported by the 73.56 ± 3.95% of cells in the G0/G1 phase, as observed in Figure 5B.

**Figure 6:**
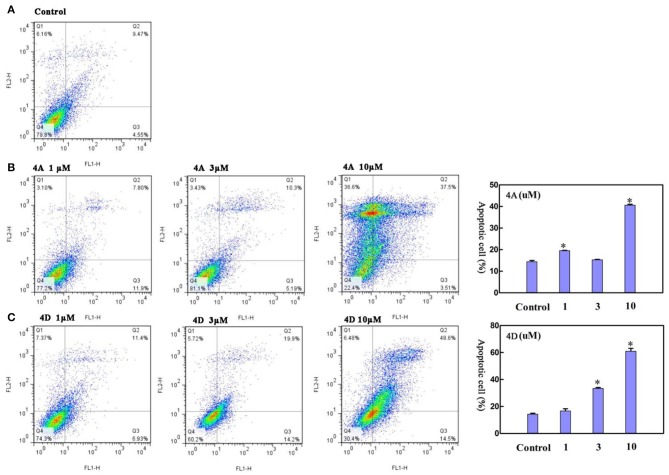
Effect of **4A** and **4D** on apoptosis in MCF-7 cells. Flow cytometric analyses of MCF-7 cells after Annexin IV staining show that compared to controls **(A)**, **4A** exposure **(B)** and 4D exposure **(C)** result in apoptosis in MCF-7 cells in a concentration. Data are reported as mean ± SD. *P < 0.001, compared to the control group.

Next, we measured the apoptotic rate of MCF-7 cells at different concentrations of treatment to detect whether 4A and 4D can induce apoptosis. As shown in Figure 6, during treatment of MCF-7 cells for 48 h with 1 and 3 μM of 4A, 19.63 ± 0.11% and 15.40 ± 0.13% apoptotic rates are given by the contrast of 14.50 ± 0.68% in the non-treated group, which suggests that 1 or 3 μM of 4A is not enough to induce apoptosis in MCF-7 cells. Subsequently, treatment of MCF-7 cells with 10 μM of 4A resulted in the progressive apoptotic rate of 40.74 ± 0.38%. This proliferation is also observed after the treatment of 4D at concentrations of 3 and 10 μM, supported by apoptotic rates of 33.55 ± 0.78% and 61.15 ± 2.76% (Figure 6C). Therefore, we conclude that both 4A and 4D can induce cell apoptosis in MCF-7 cells as well as cell cycle arrest in G0/G1 phase.

## Conclusion

In summary, this study describes the optimization of the anti-tumor agent CLX-4 by employing the drug design strategies, X-ray crystallography, and cell-based screening. With the comparison to CLX-4, dihomooxacalix[4]arene derivative 4A demonstrates the much more efficient cytotoxic effect against the MCF-7 and HeLa cell lines with IC_50_ of 1.0 ± 0.1 μM and 0.8 ± 0.2 μM, respectively. Derivative 4D has an IC_50_ of 0.7 ± 0.1 μM against A549 cell lines, but failed due to the high toxicity on HUVEC cell lines. The underlying mechanism of cytotoxic effects indicates that they can induce the MCF-7 cell cycle arrest in G0/G1 phase and cell apoptosis. In this context, it is also important to note that we provide a new look into drug discovery by using the old supramolecuar scaffold to design potent anti-tumor agents.

## Data Availability Statement

The detailed spectroscopic data including crystallographic data (CIF) of compounds are available. Single crystal data for compounds **4L** and **4N** have been deposited in the Cambridge Crystallographic Data Center and assigned to deposition numbers CCDC 1915578 and 1569156. All datasets generated for this study are included in the article/Supplementary Material.

## Author Contributions

LA and CY designed the work. LH, CW, and JL made contributions to the experiments and collective data. The paper was written by LA. YZ, TH, and JS provide the technical support. All authors extensively discussed the results, reviewed the manuscript, extensively discussed the results, reviewed the manuscript, and approved the final version of the manuscript to be submitted.

### Conflict of Interest

The authors declare that the research was conducted in the absence of any commercial or financial relationships that could be construed as a potential conflict of interest.
