# Supplementary material for: Structural Design, Synthesis, and Preliminary Biological Evaluation of Novel Dihomooxacalix[4]arene-Based Anti-tumor Agents
Source: Front Chem. 2019 Dec 13;7:856. doi: 10.3389/fchem.2019.00856 (PMC6923765; doi:10.3389/fchem.2019.00856)

Structural Design, Synthesis and Preliminary Biological Evaluation of Novel Dihomooxacalix[4]arene based Anti-tumor Agents

Lin An[1,2], Chan Wang[1,2], Lili Han[3], Jiadong Liu[1,2], Tonghui Huang[1,2], Youguang Zheng[1,2], Chaoguo Yan[4]* and Jing Sun[4]*

[1] College of Pharmacy, Xuzhou Medical University, Xuzhou, China, [2] Jiangsu Key Laboratory of New Drug Research and Clinical Pharmacy, Xuzhou Medical University, Xuzhou, China, [3] Children's Hospital Affiliated to Zhengzhou University, Zhengzhou, China, [4] College of Chemistry and Chemical Engineering, Yangzhou University, Yangzhou, China

Table.S1 X-ray crystallographic data of compound 4L and 4N

Phase	**4L**	**4N**
Empirical formula	$C_{59}H_{84}N_2O_7$	$C_{63}H_{76}N_2O_7$
Formula weight	933.28	973.26
Temperature (K)	293(2)	196(2)
Wavelength/nm	0.71073	0.71073
Crystal system	Monoclinic	Monoclinic
space group	P 21/c	C 2/c
a (Å)	11.565(2)	24.451(3)
b (Å)	22.834(4)	26.022(3)
c (Å)	22.585(4)	18.891(2)
α(°)	90	90
β(°)	94.237(5)	114.333(3)
γ(°)	90	90
Volume($Å^3$)	5947.8(18)	10952(2)
Z	4	8
F(000)	2032	4192
Calculated density ($g.cm^{-3}$)	1.042	1.181
Absorption coefficient (mm^{-1})	0.067	0.076
θ range (°)	1.27 to 27.47	1.93 to 27.64
Limiting indices	-14<=h<=14, -29<=k<=29, -28<=l<=29	-26<=h<=31, -33<=k<=33, -24<=l<=24
Reflections collected / unique	76650 / 13487 [R(int) = 0.1141]	61187 / 12661 [R(int) = 0.1090]
Completeness to theta	99.1 %	99.1 %
Max. and min. transmission	0.987 and 0.983	0.985 and 0.982
Refinement method	Full-matrix least-squares on F^2	Full-matrix least-squares on F^2
Data / restraints / parameters	13487 / 0 / 629	12661 / 0 / 651
Goodness-of-fit on F^2	1.042	1.012
Final R indices [I > 2sigma(I)]	R_1 = 0.0784, wR_2 = 0.2093	R_1 = 0.0766, wR_2 = 0.1586
R indices (all data)	R_1 = 0.1253, wR_2 = 0.2266	R_1 = 0.2085, wR_2 = 0.1997
Largest diff. peak and hole(e. $Å^{-3}$)	0.773 and -0.500	0.573 and -0.485

Figure S1. ^{1}H NMR, ^{13}C NMR spectra of 2A

^{1}H NMR (400 MHz , $CDCl_3$) of 2A

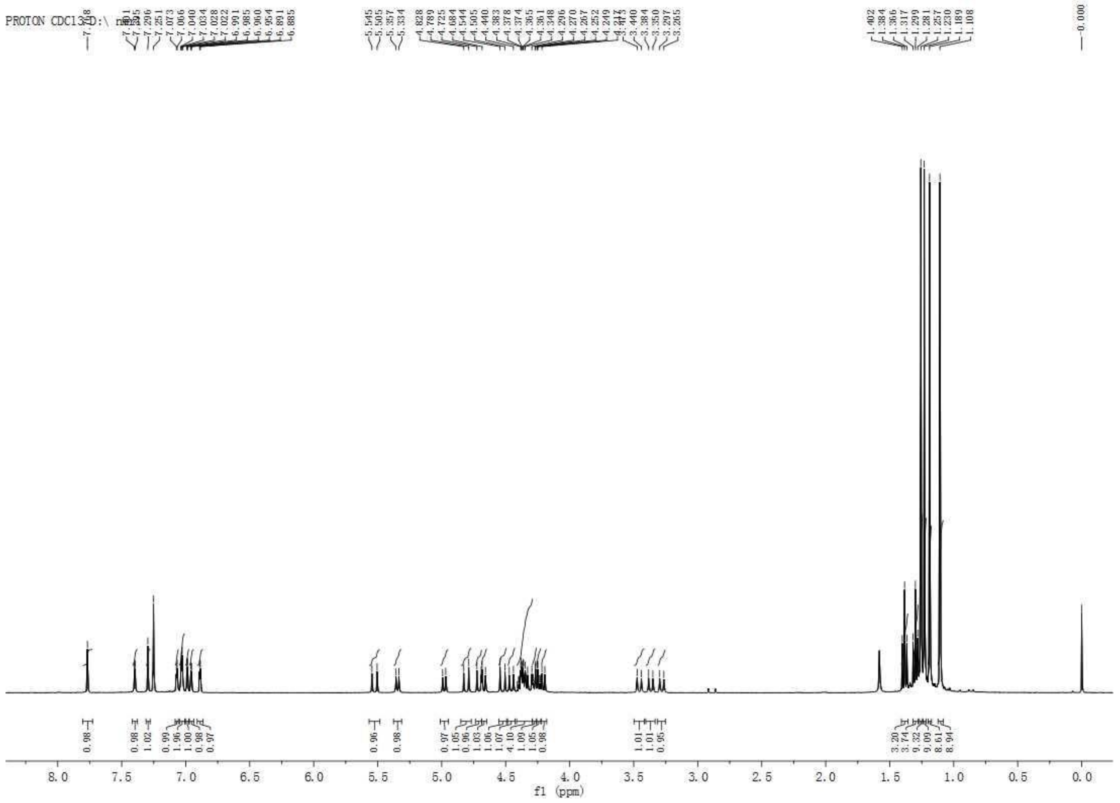

^{13}C NMR (100 MHz, $CDCl_3$) of 2A

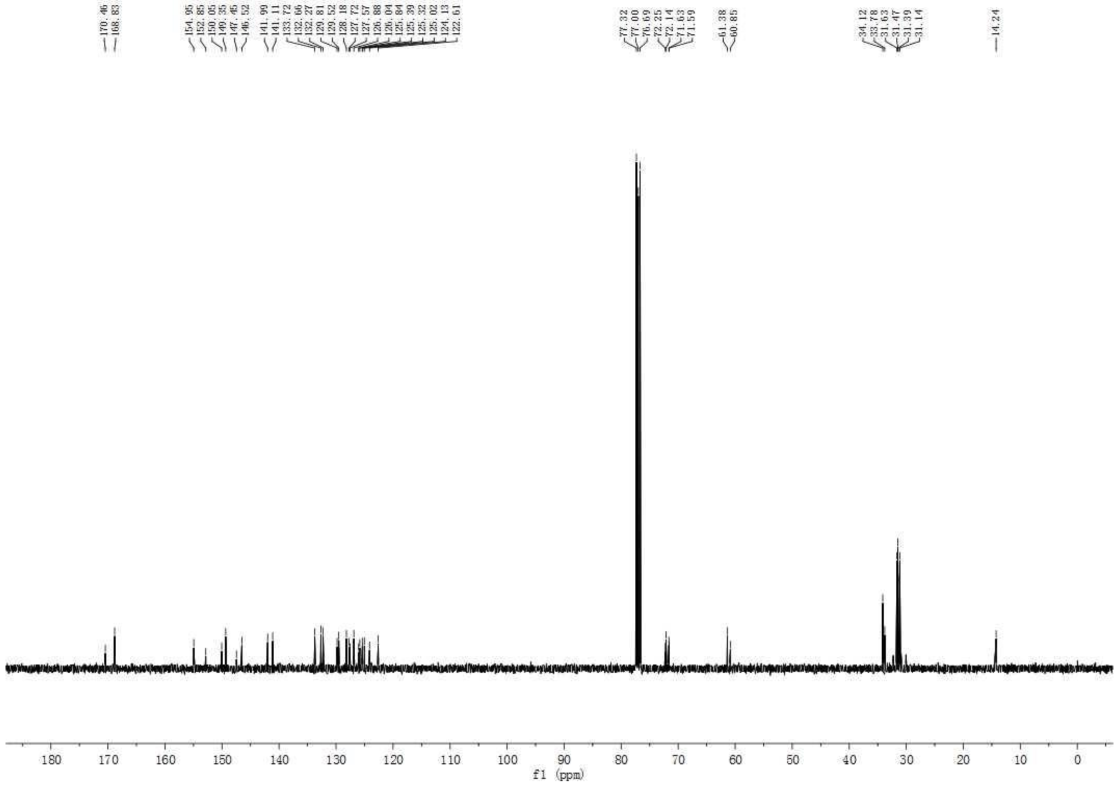

Figure S2. ^{1}H NMR,^{13}C NMR and HRMS spectra of 3A

^{1}H NMR (400 MHz, $CDCl_3$) of 3A

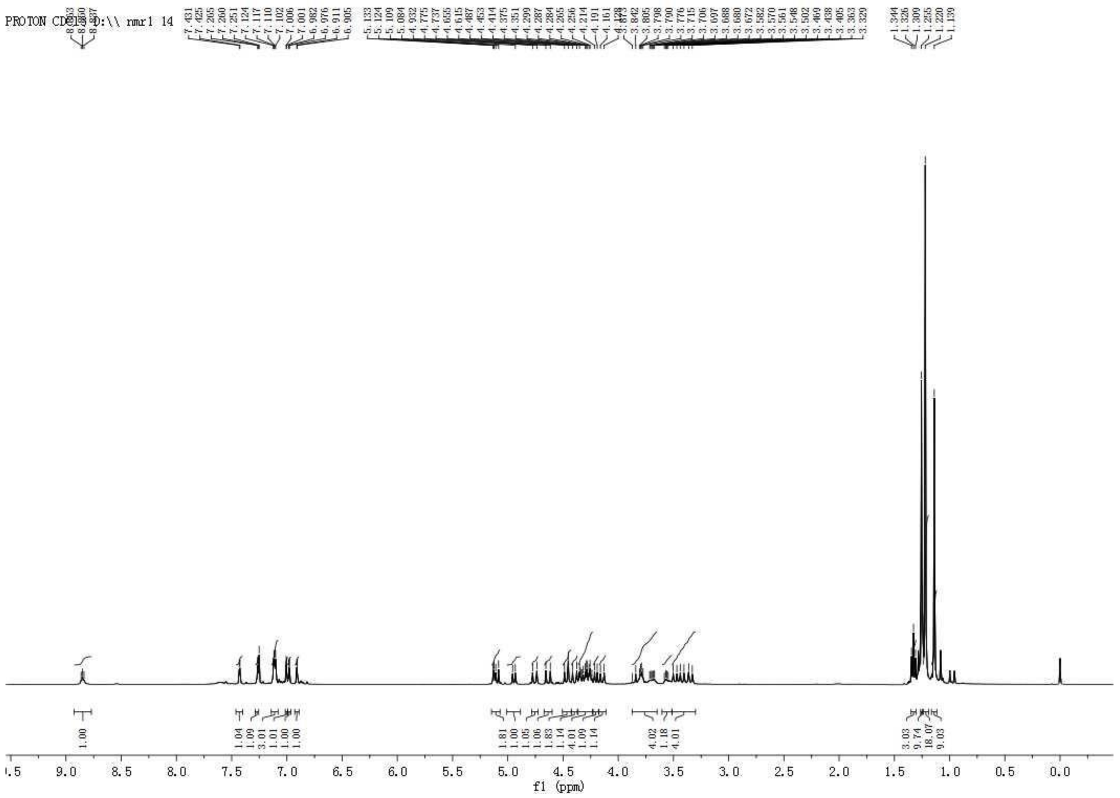

^{13}C NMR (100 MHz, $CDCl_3$) of 3A

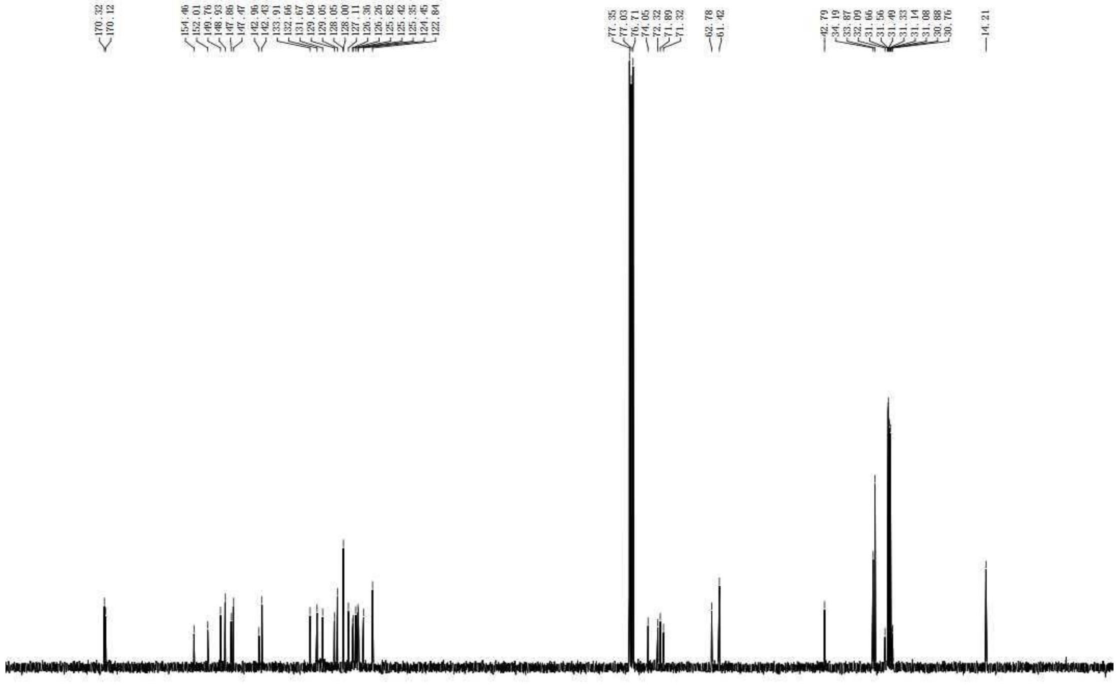

HRMS spectrum of 3A

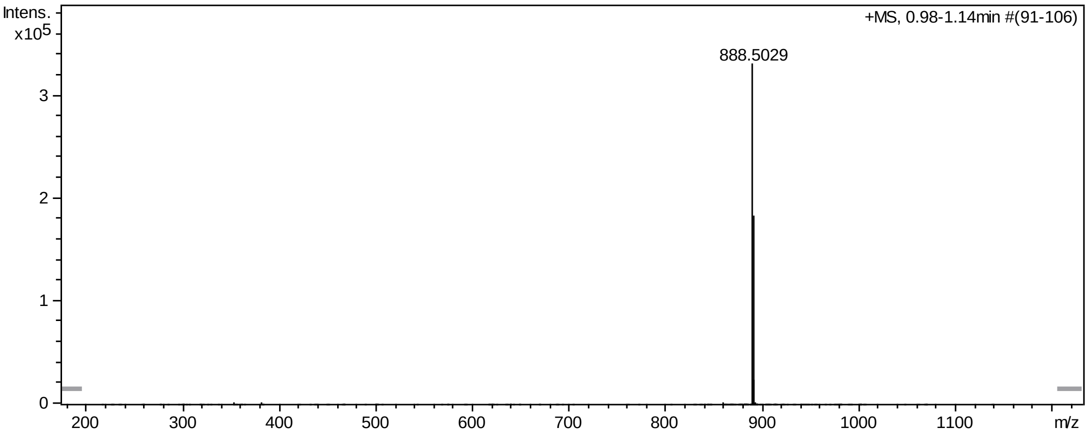

Figure S3. ^{1}H NMR,^{13}C NMR and HRMS spectra of 4A

^{1}H NMR (400 MHz, $CDCl_3$) of 4A

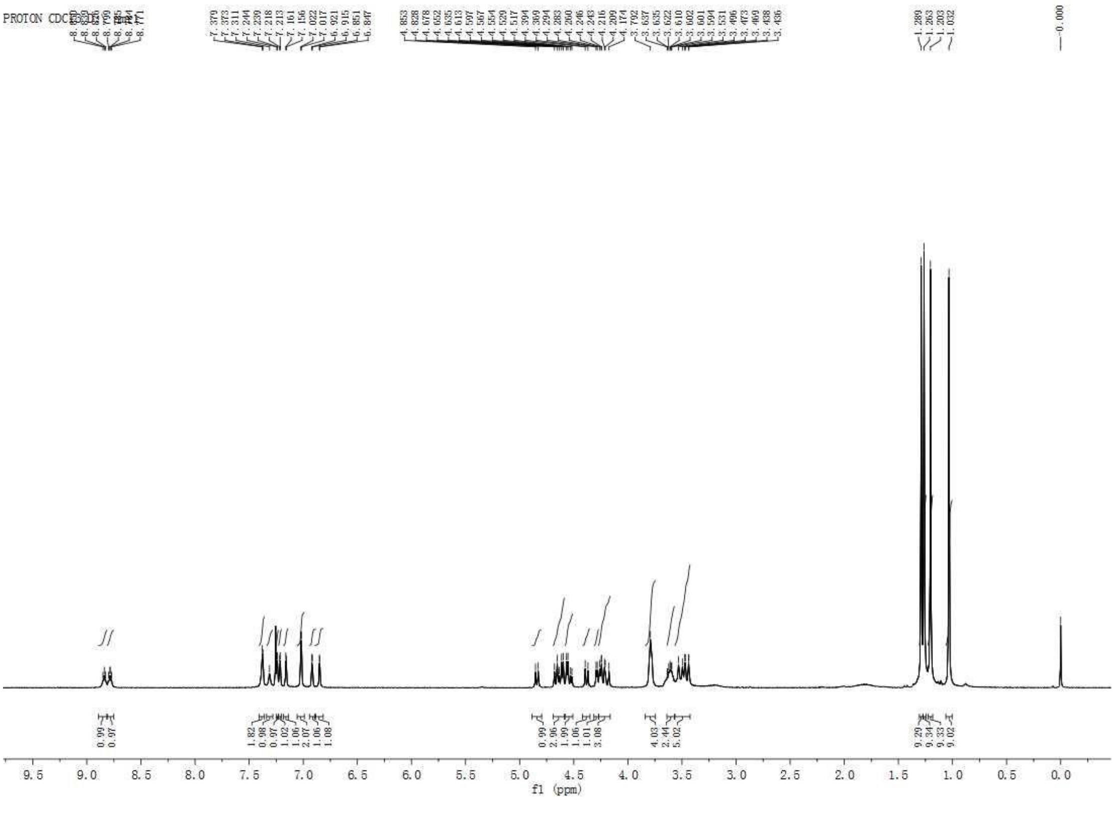

^{13}C NMR (100 MHz, $CDCl_3$) of 4A

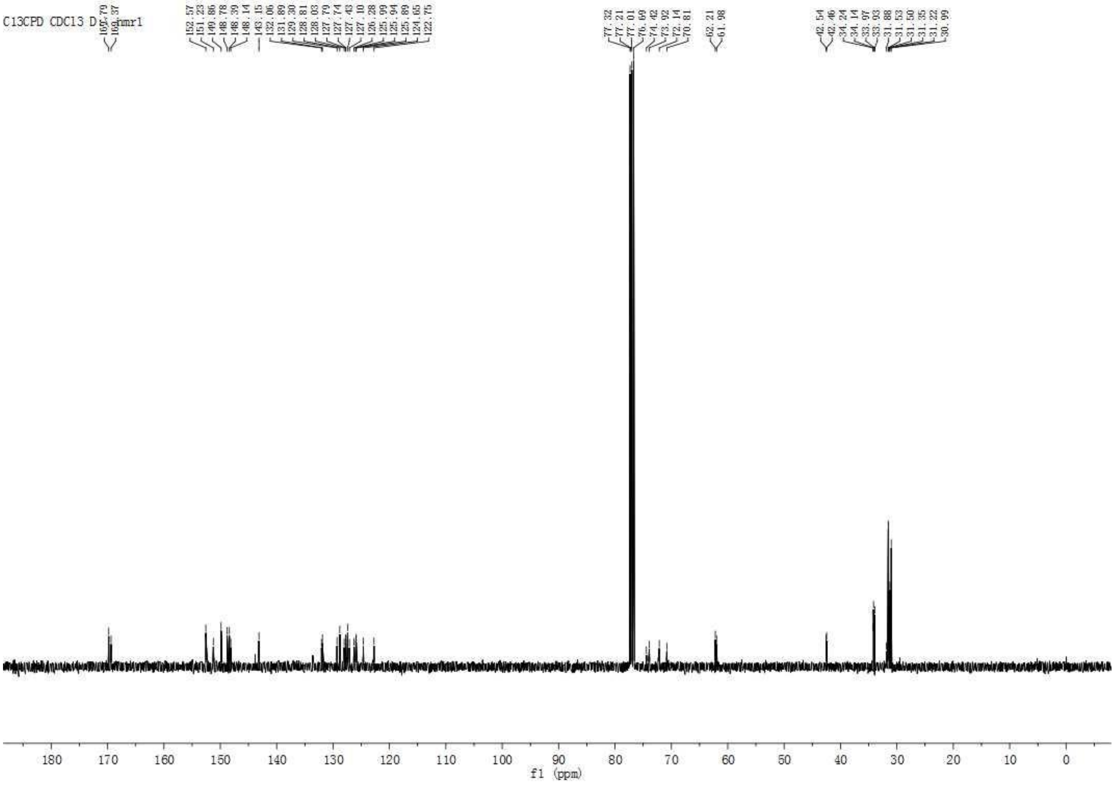

HRMS spectrum of 4A

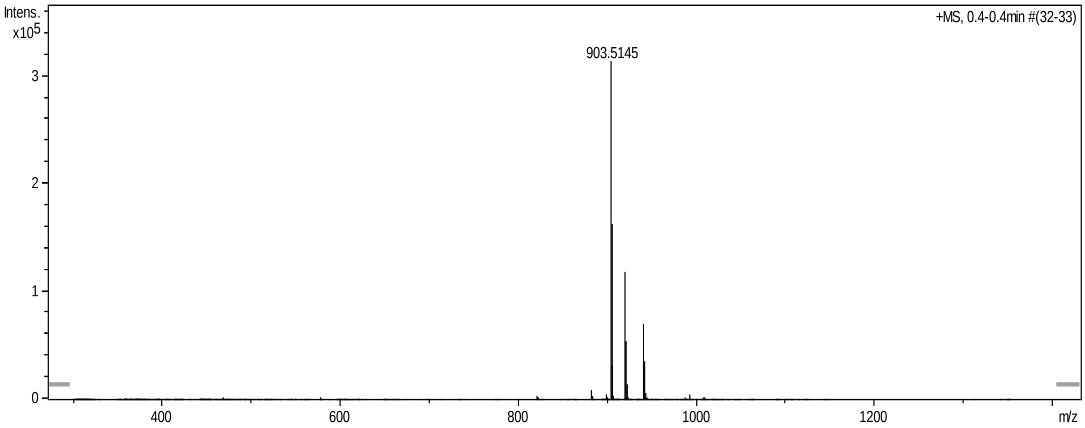

Figure S4. ^{1}H NMR,^{13}C NMR and HRMS spectra of 4B

^{1}H NMR (400 MHz, DMSO-d_6) of 4B

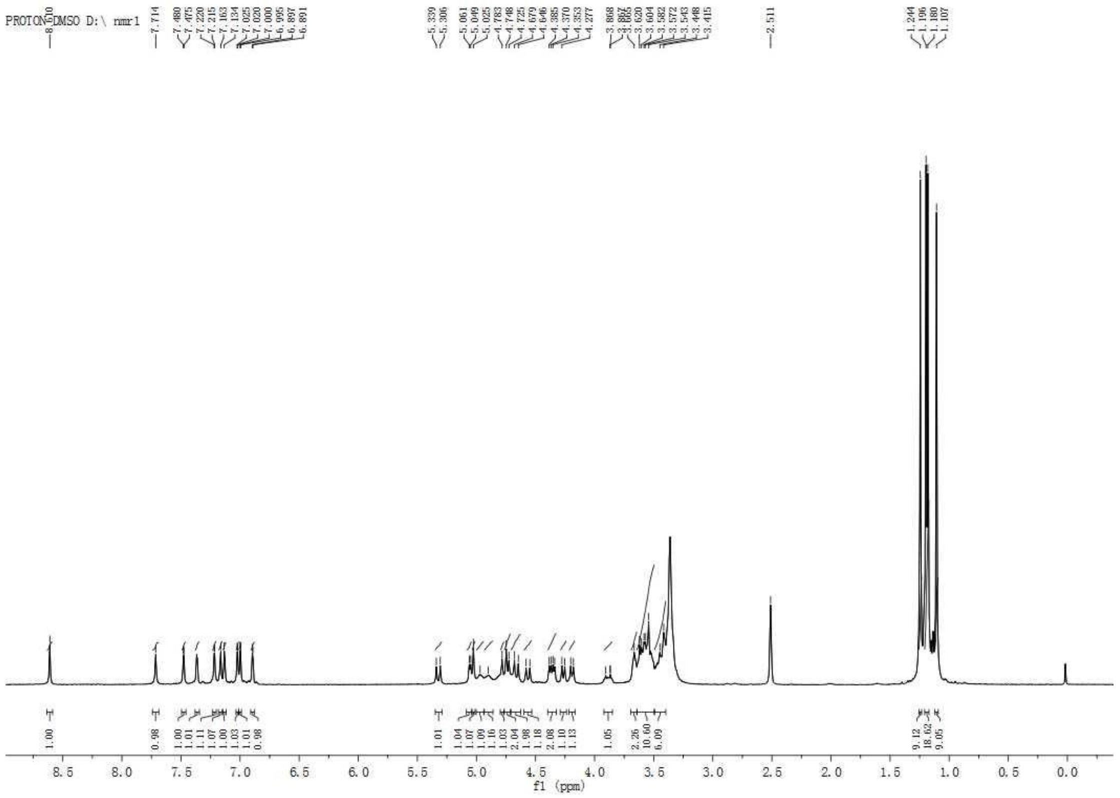

^{13}C NMR (100 MHz, DMSO-d_6) of 4B

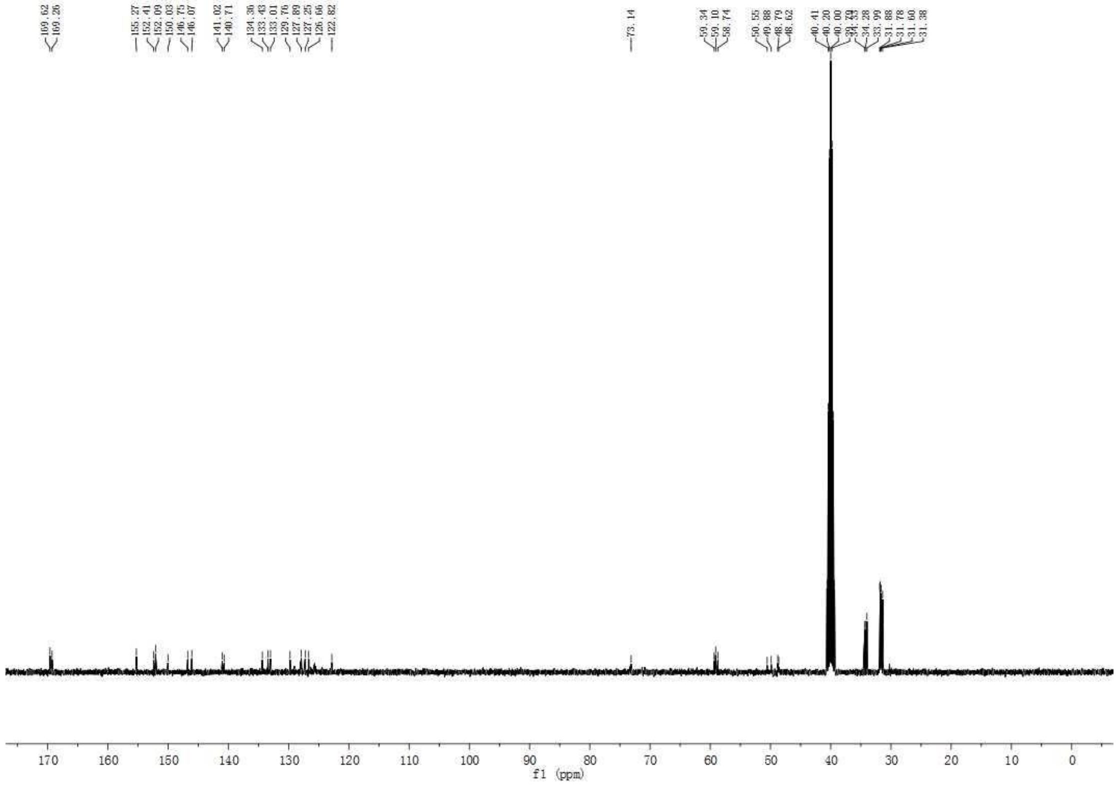

HRMS spectrum of 4B

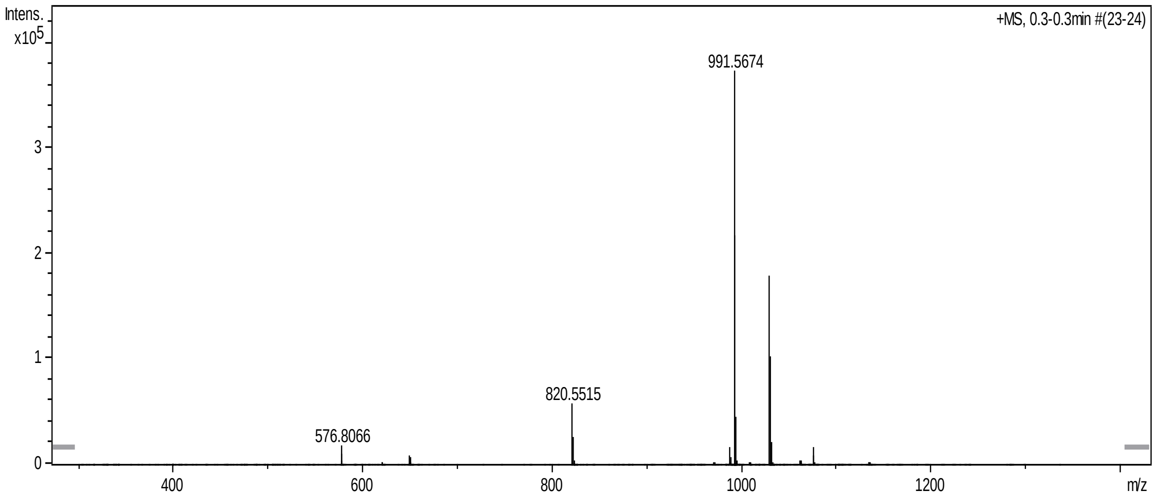

Figure S5. ^{1}H NMR, ^{13}C NMR and HRMS spectra of 4C

^{1}H NMR (400 MHz, $CDCl_3$) of 4C

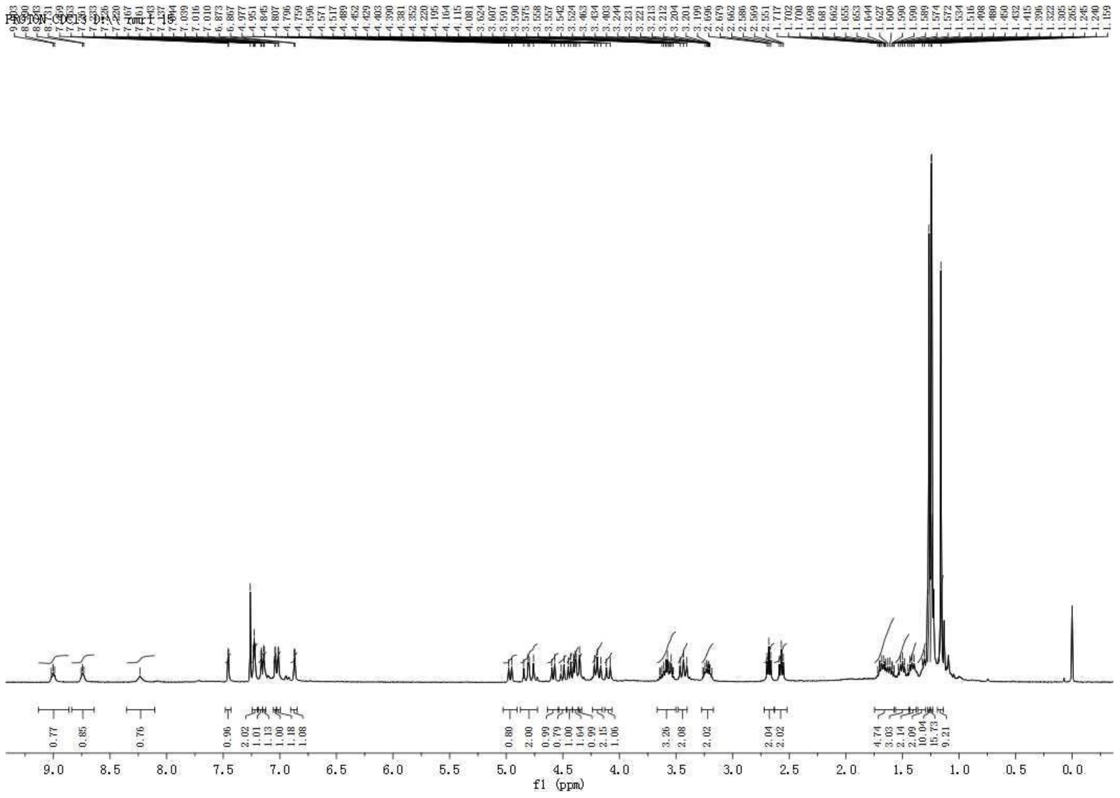

^{13}C NMR (100 MHz, $CDCl_3$) of 4C

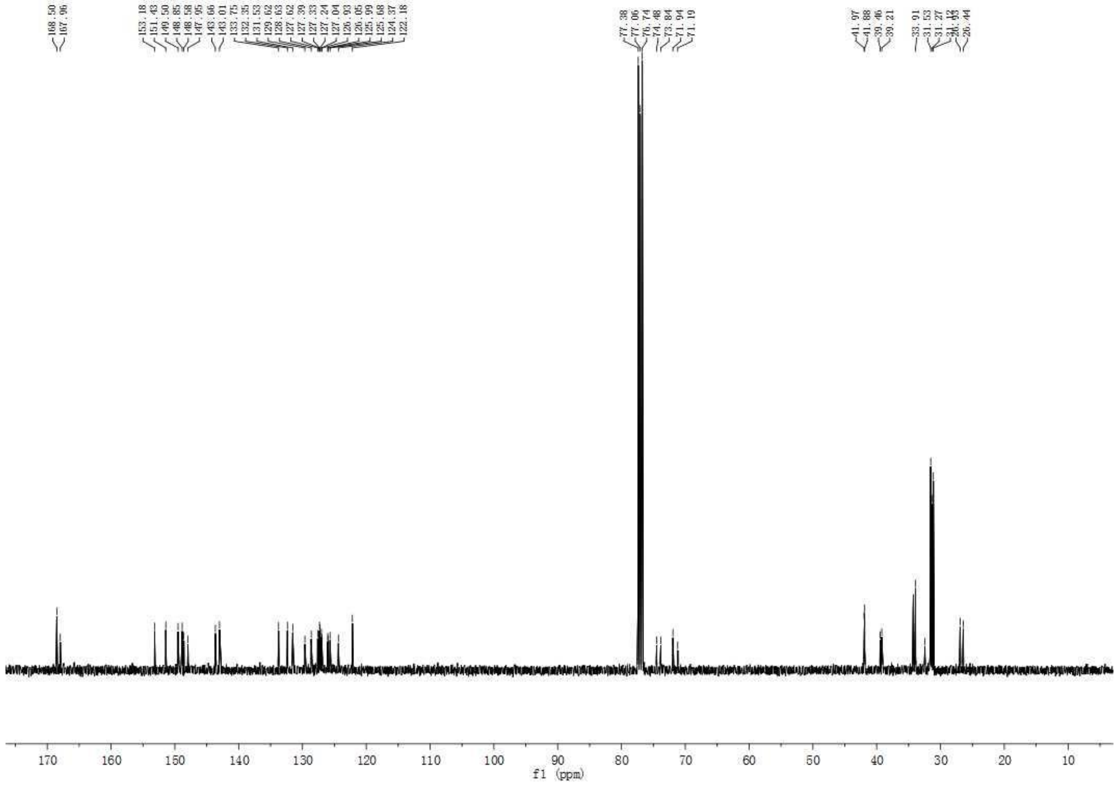

HRMS spectrum of 4C

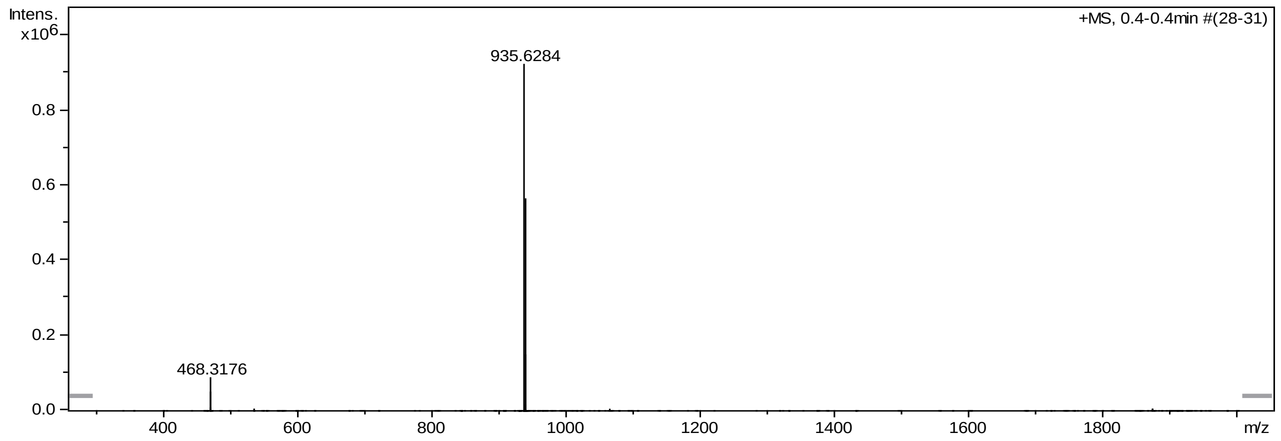

Figure S6. ^{1}H NMR,^{13}C NMR and HRMS spectra of 4D

^{1}H NMR (400 MHz, $CDCl_3$) of 4D

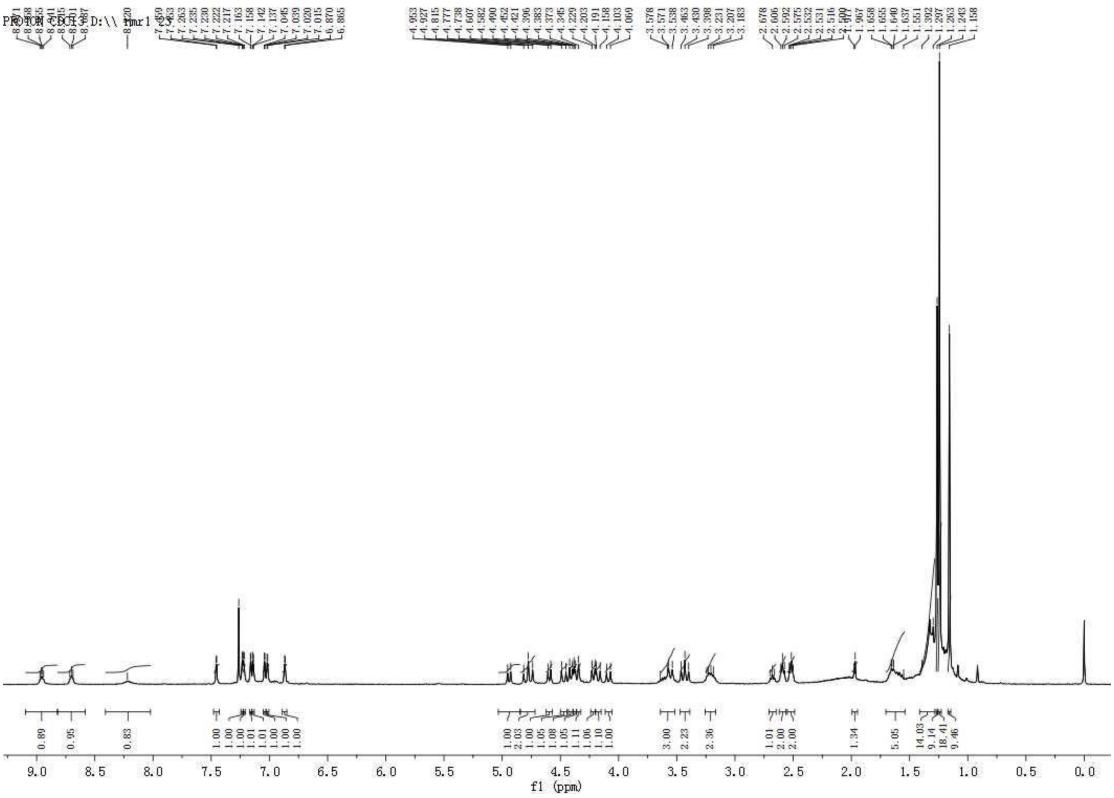

^{13}C NMR (100 MHz, $CDCl_3$) of 4D

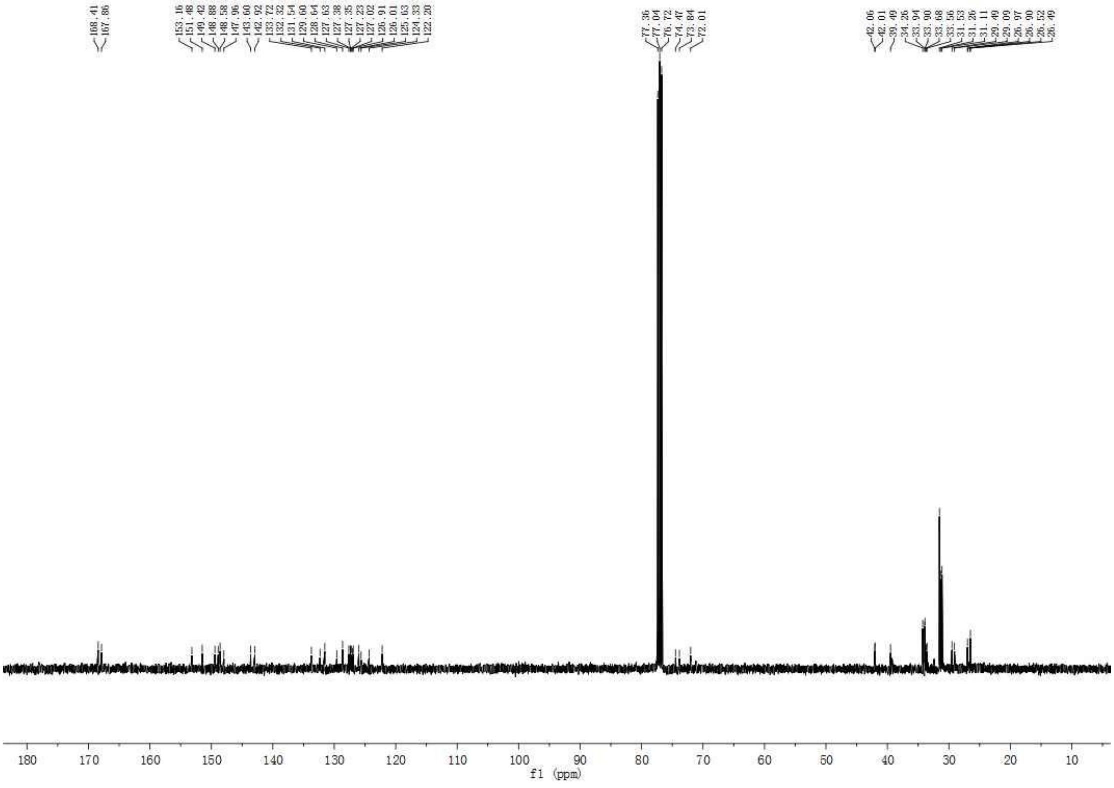

HRMS spectrum of 4D

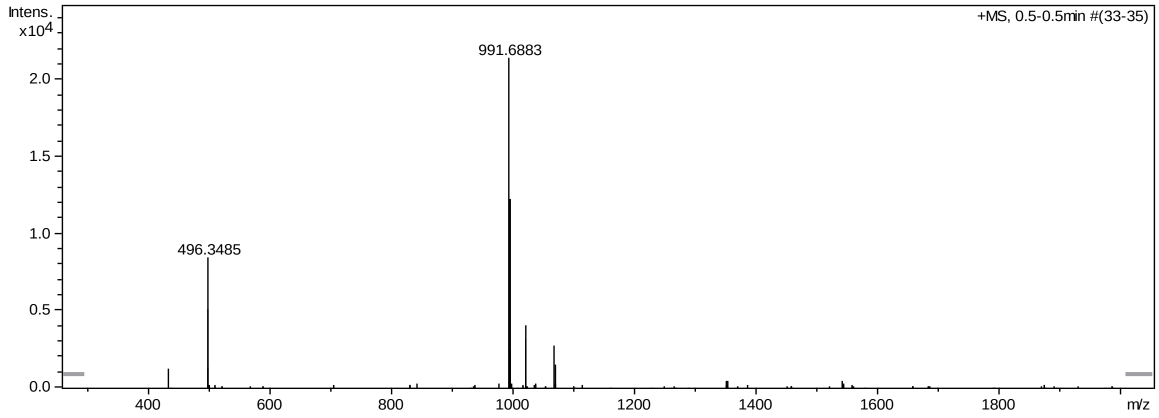

Figure S7. ^{1}H NMR, ^{13}C NMR and HRMS spectra of 4E

^{1}H NMR (400 MHz, $CDCl_3$) of 4E

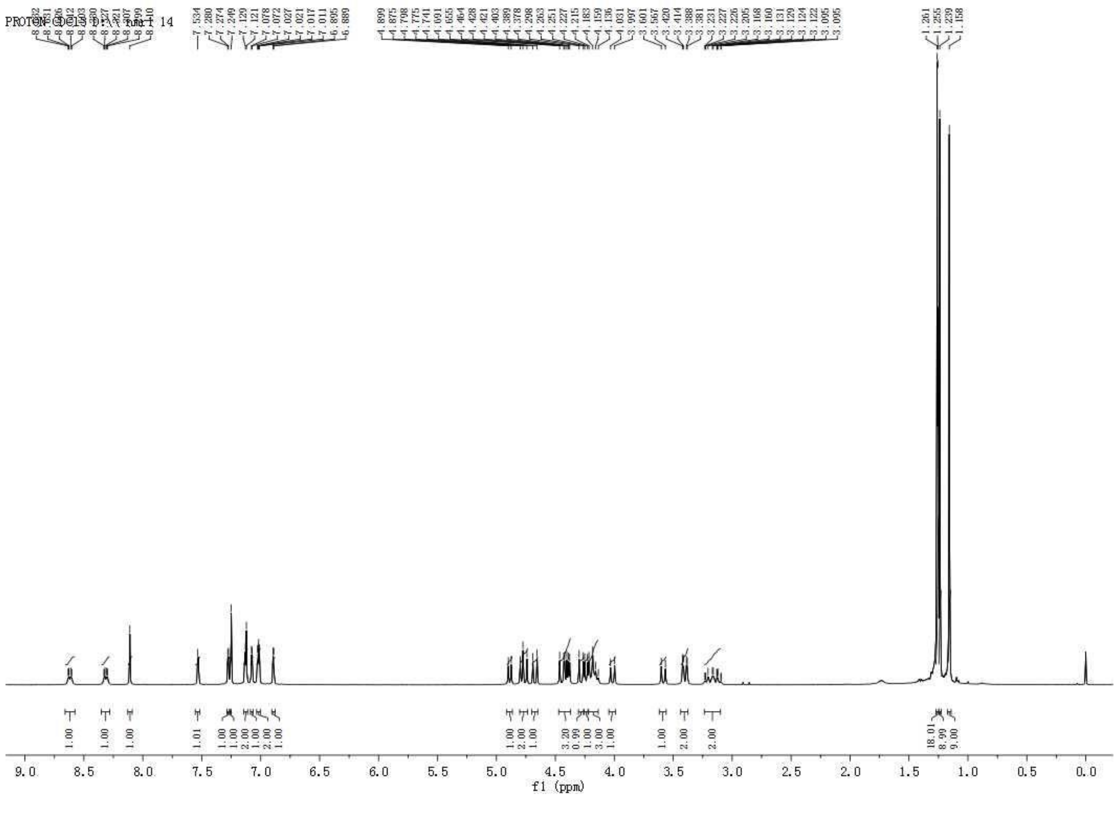

^{13}C NMR (100 MHz, $CDCl_3$) of 4E

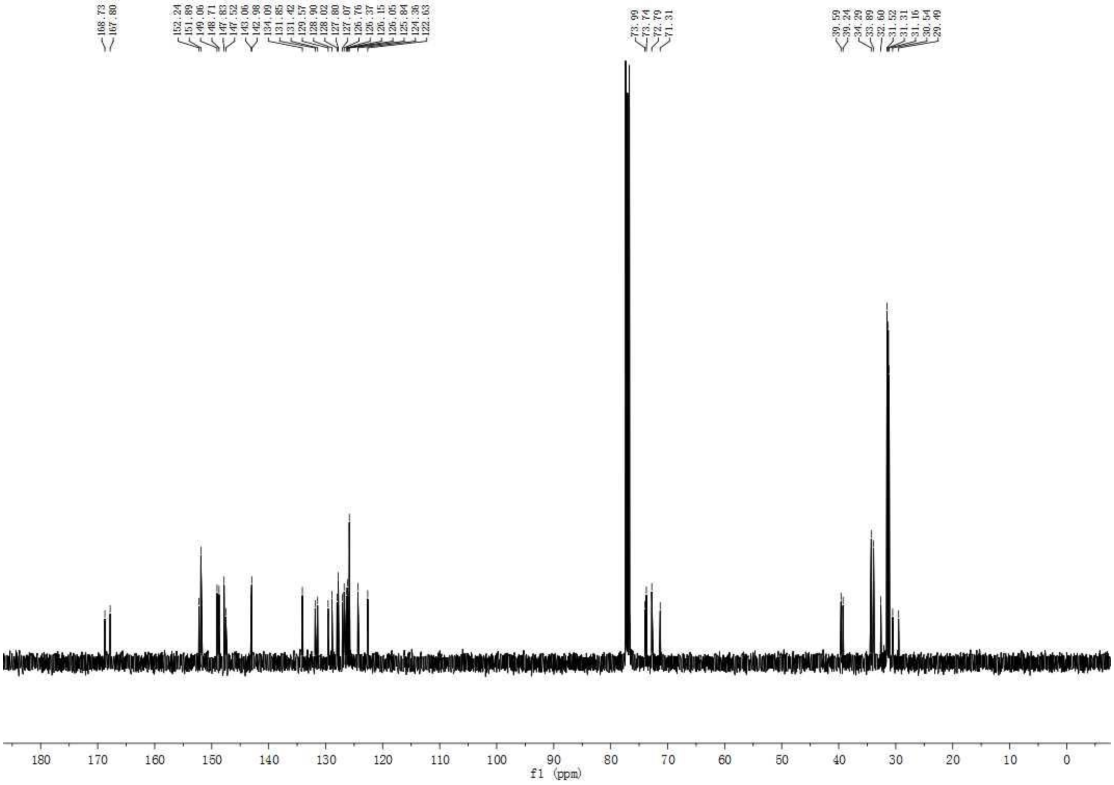

HRMS spectrum of 4E

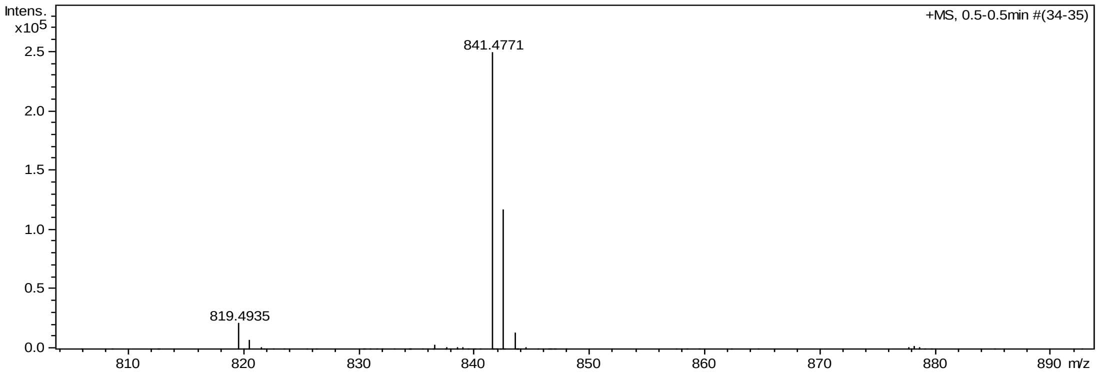

Figure S8. ^{1}H NMR,^{13}C NMR and HRMS spectra of 4F

^{1}H NMR (400 MHz, $CDCl_3$) of 4F

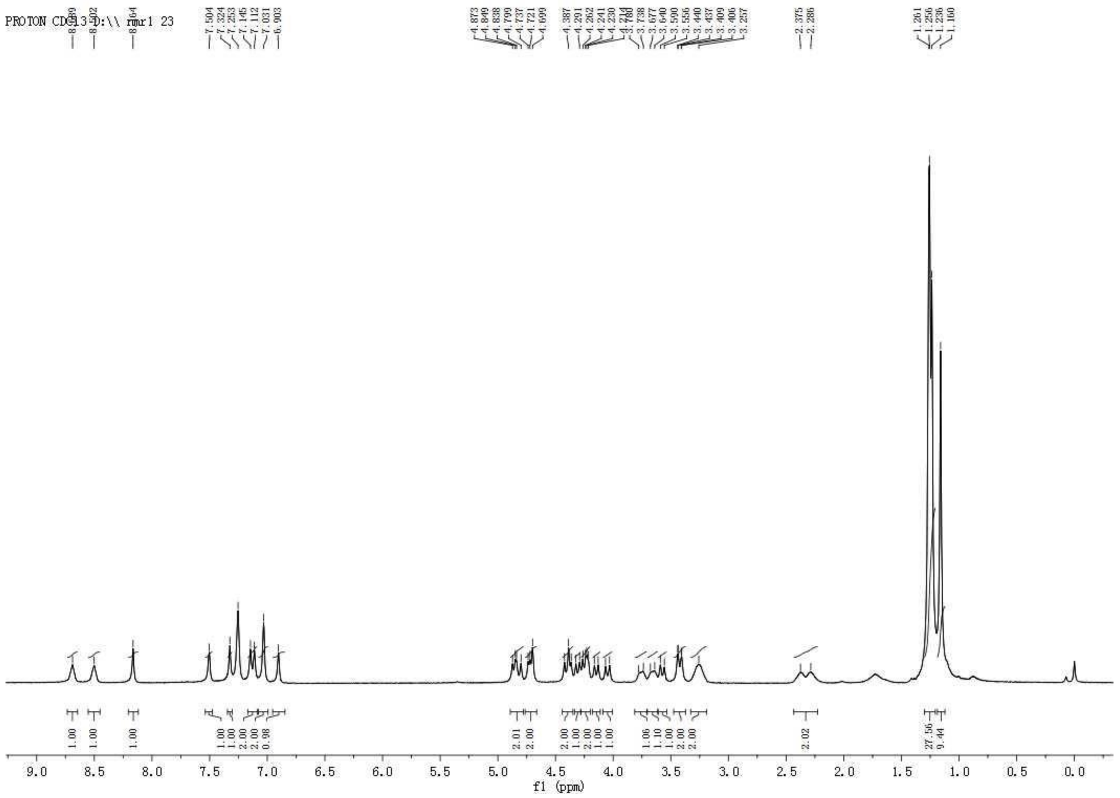

^{13}C NMR (100 MHz, $CDCl_3$) of 4F

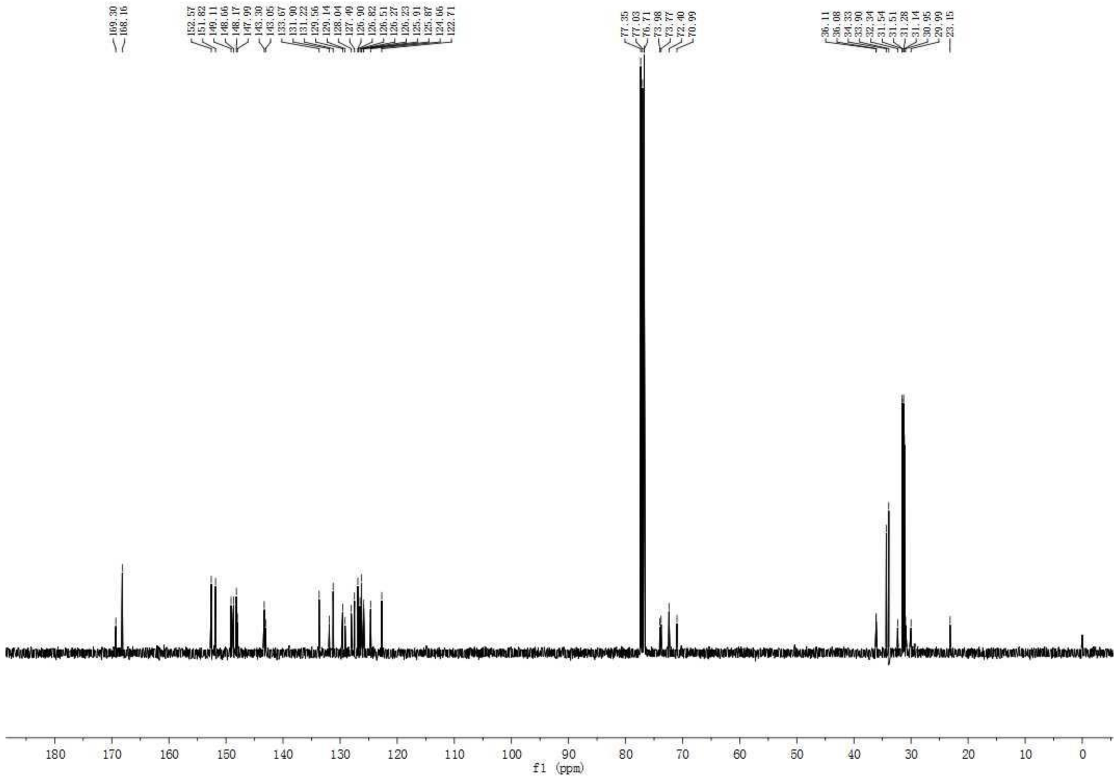

HRMS spectrum of 4F

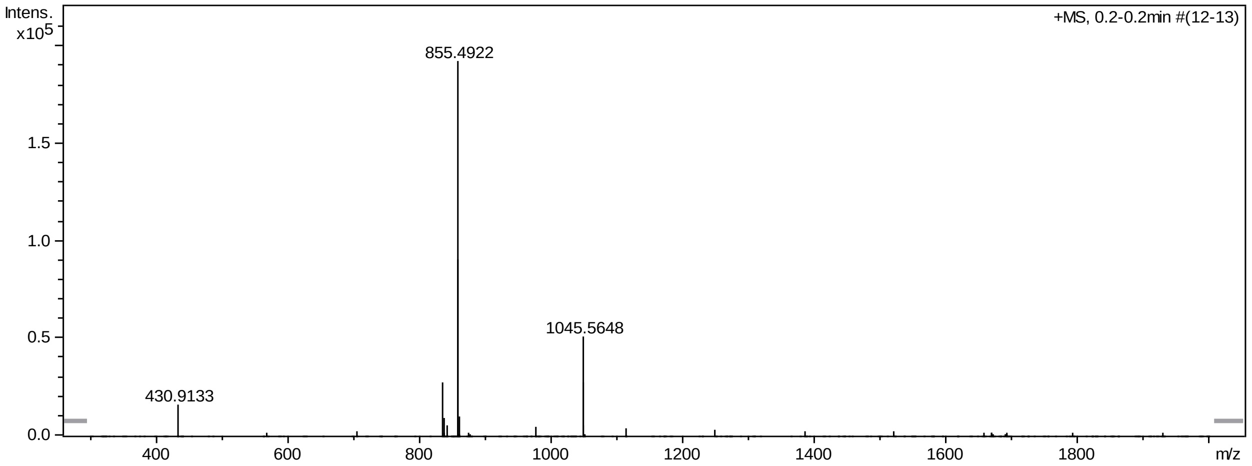

Figure S9 ^{1}H NMR,^{13}C NMR and HRMS spectra of 4G

^{1}H NMR (400MHz, DMSO-d_6) of 4G

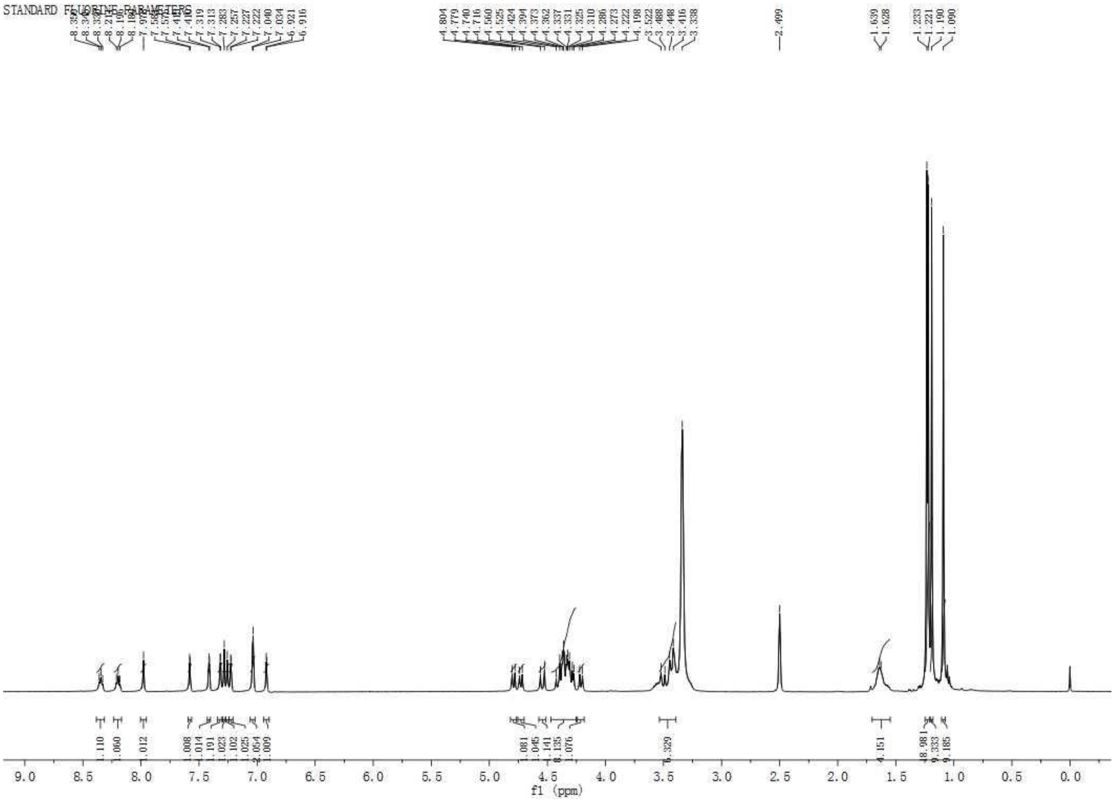

^{13}C NMR (100 MHz, $CDCl_3$) of 4G

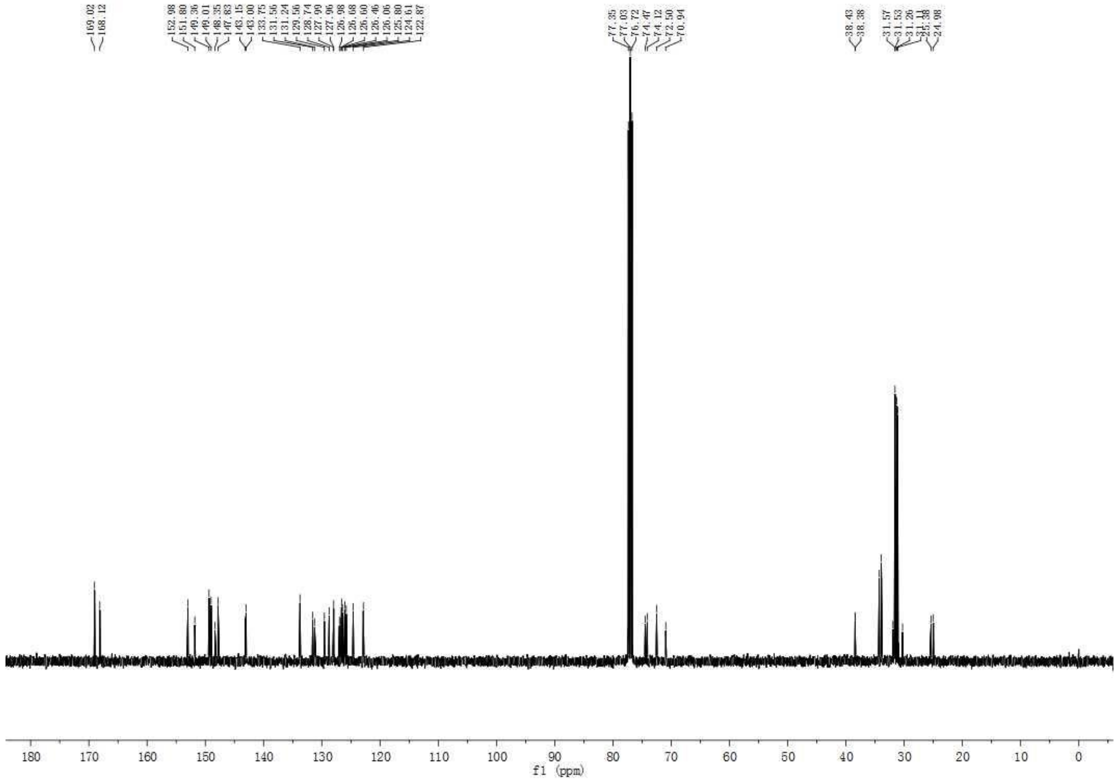

HRMS spectrum of 4G

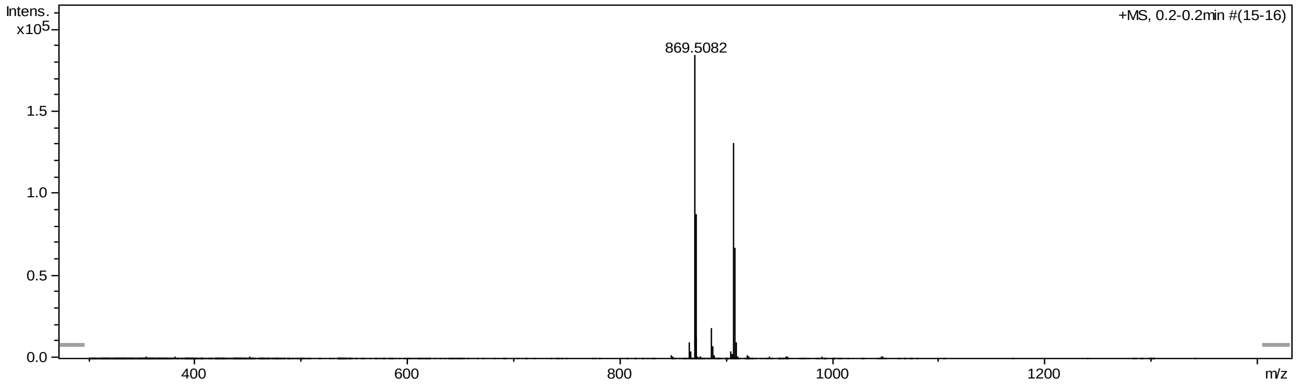

Figure S10 ^{1}H NMR,^{13}C NMR and HRMS spectra of 4H

^{1}H NMR (400 MHz, $CDCl_3$) of 4H

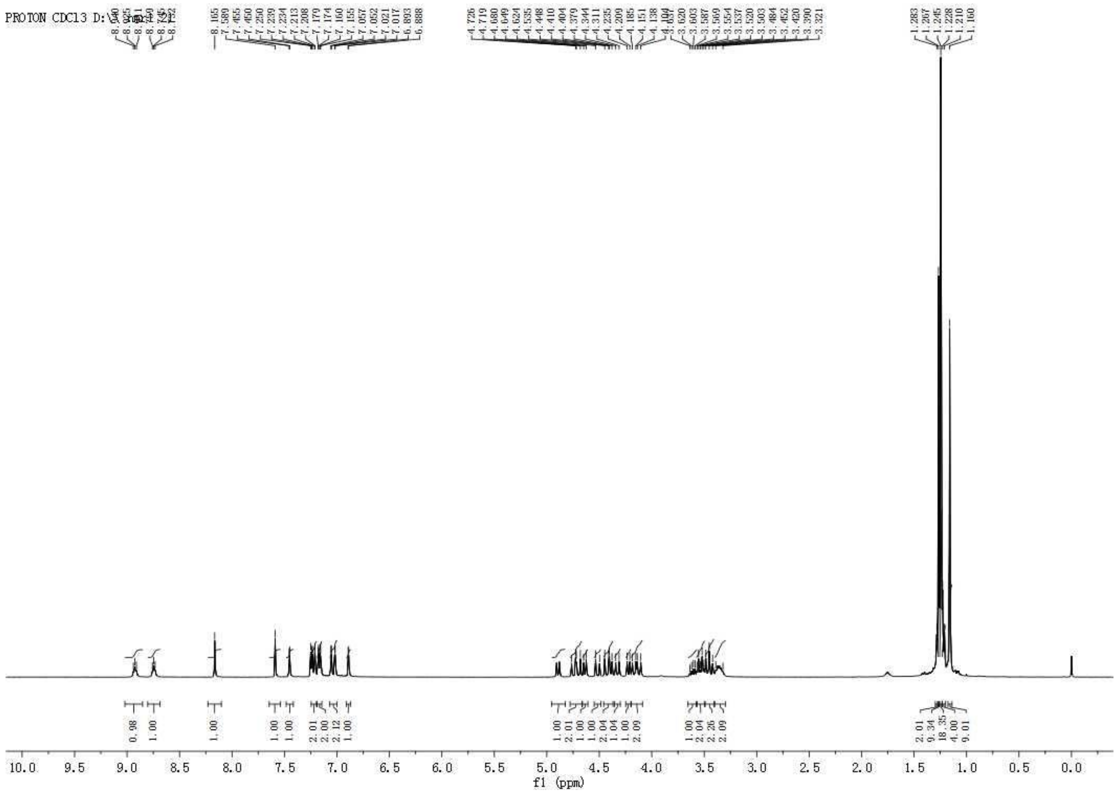

^{13}C NMR (100 MHz, $CDCl_3$) of 4H

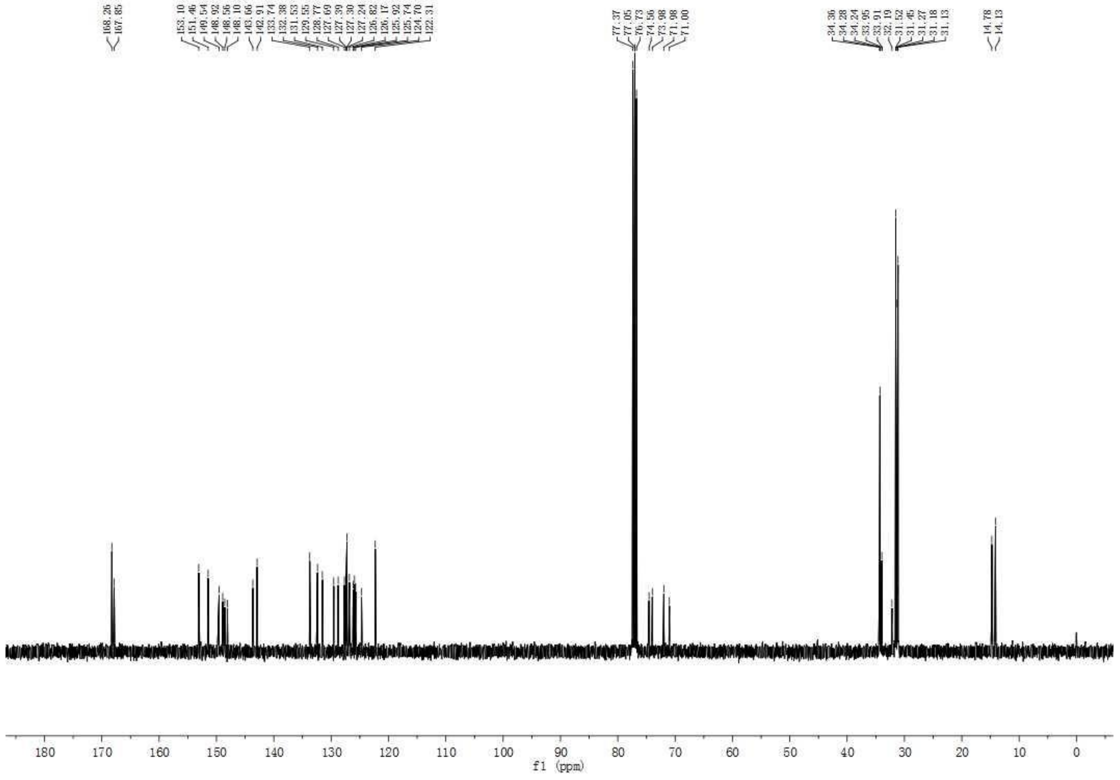

HRMS spectrum of 4H

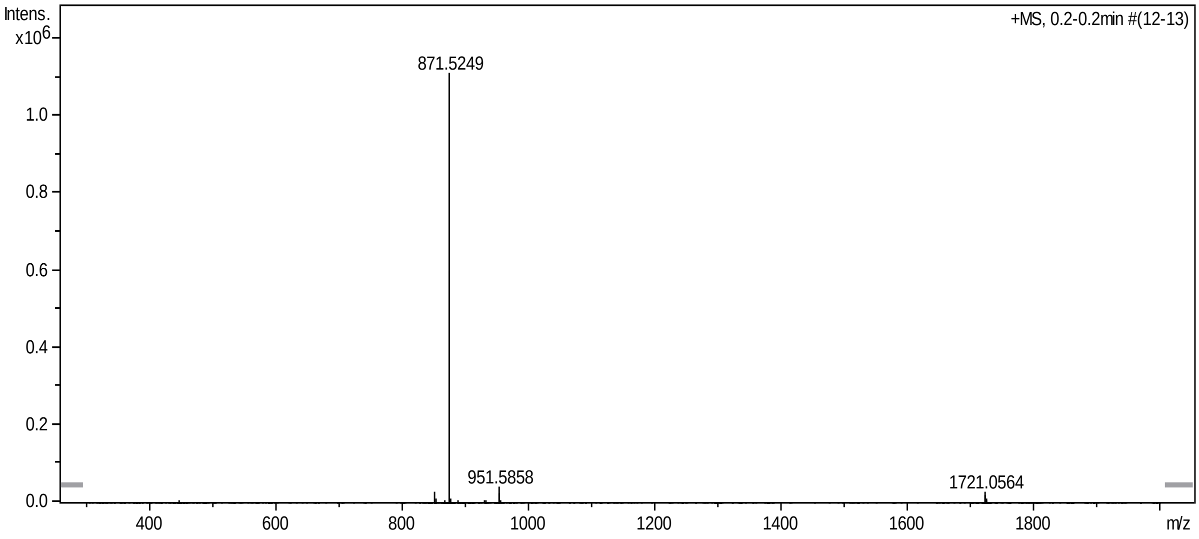

Figure S11 ^{1}H NMR,^{13}C NMR and HRMS spectra of 4I

^{1}H NMR (400 MHz, $CDCl_3$) of 4I

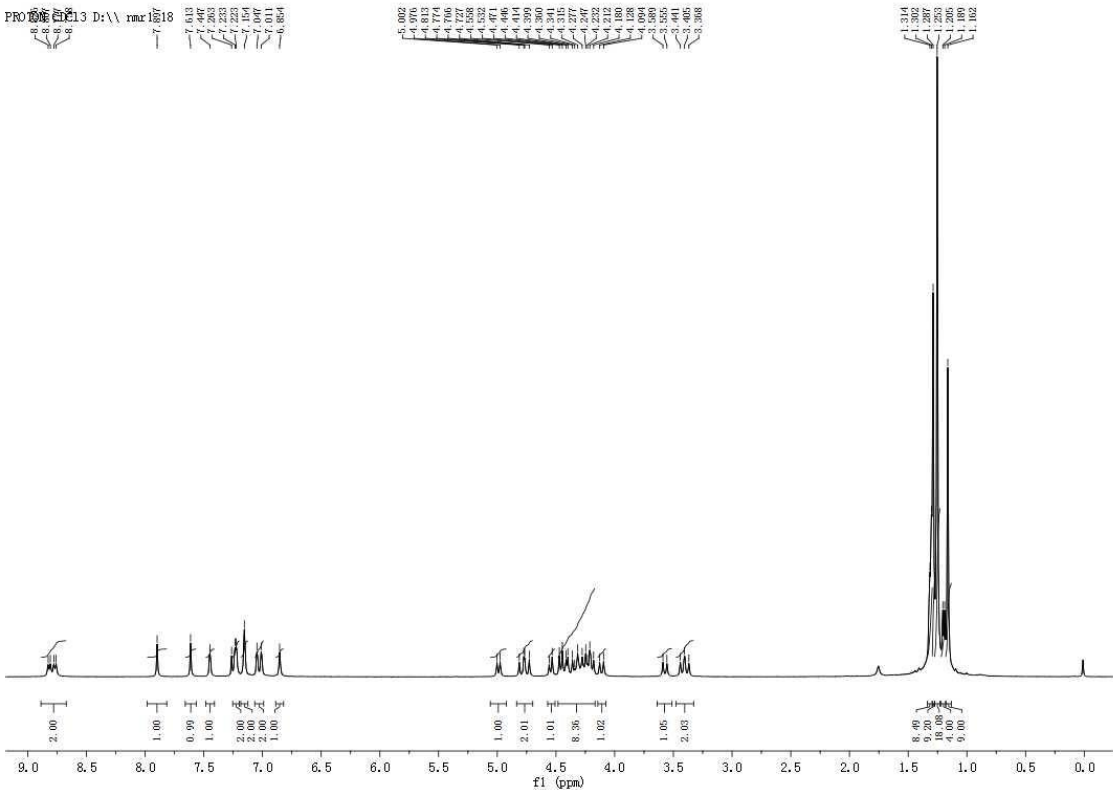

^{13}C NMR (100 MHz, $CDCl_3$) of 4I

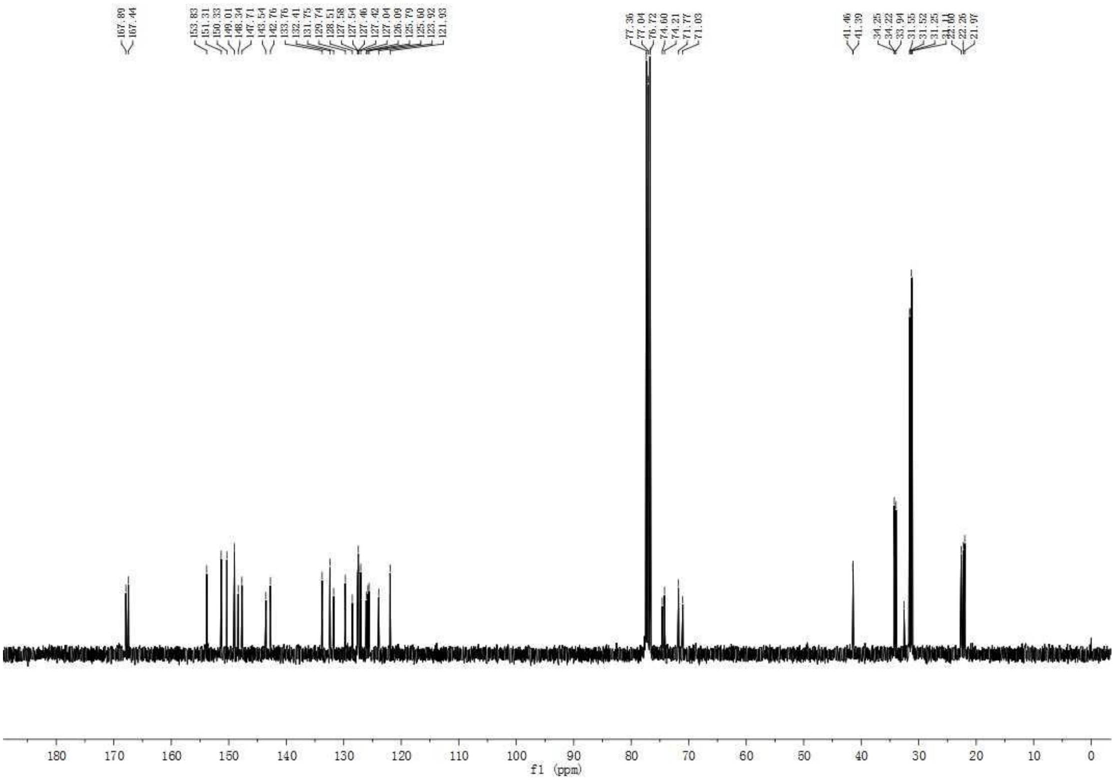

HRMS spectrum of 4I

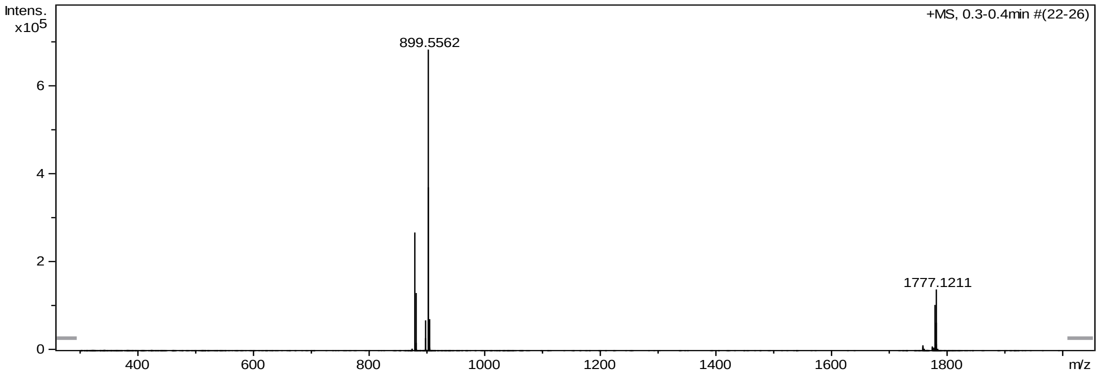

Figure S12 ^{1}H NMR, ^{13}C NMR and HRMS spectra of 4J

^{1}H NMR (400 MHz, $CDCl_3$) of 4J

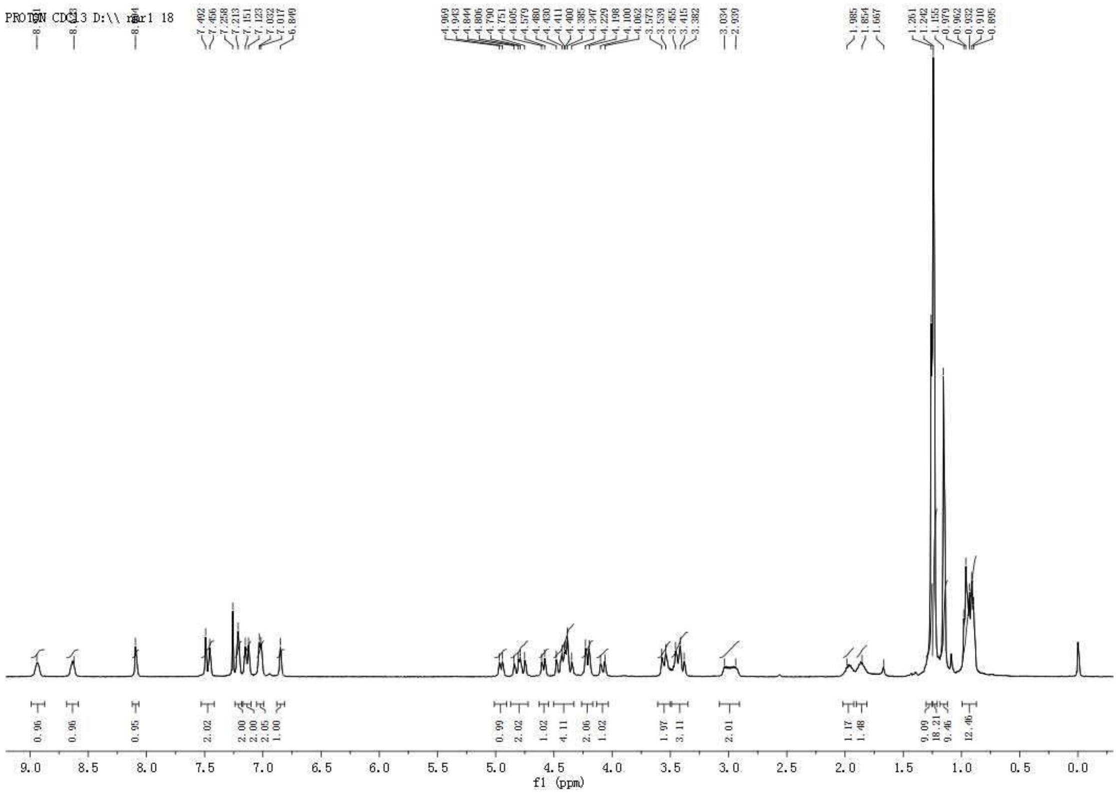

^{13}C NMR (100 MHz, $CDCl_3$) of 4J

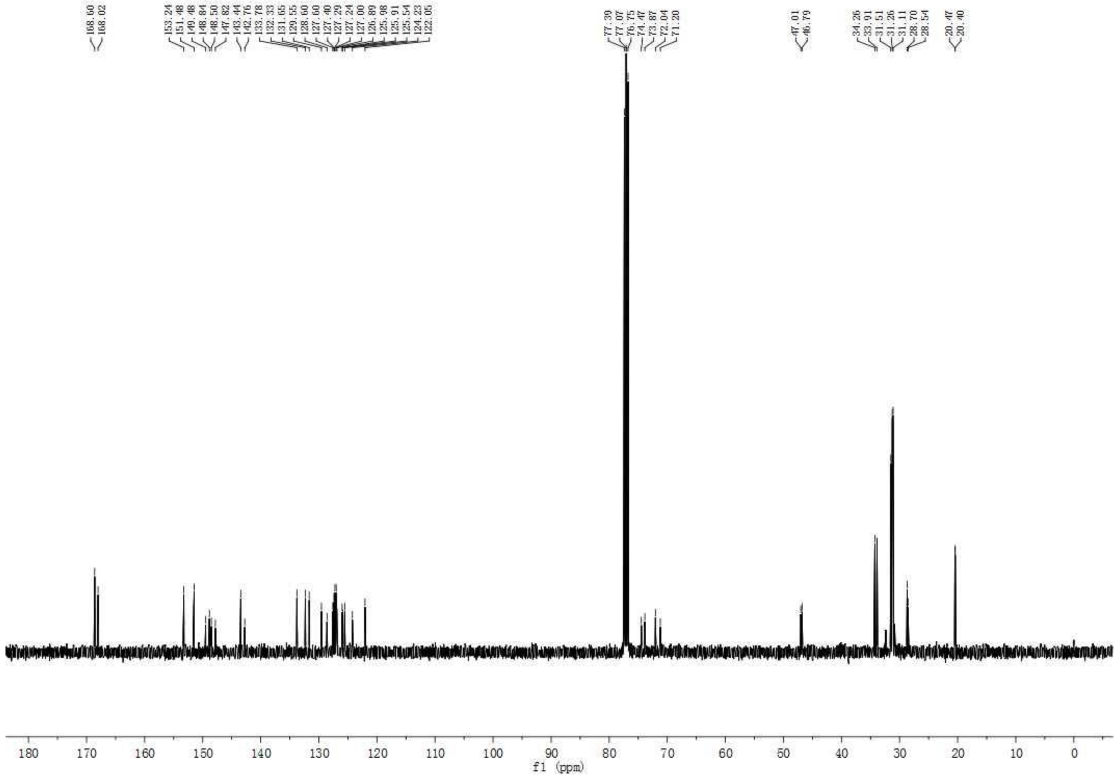

HRMS spectrum of 4J

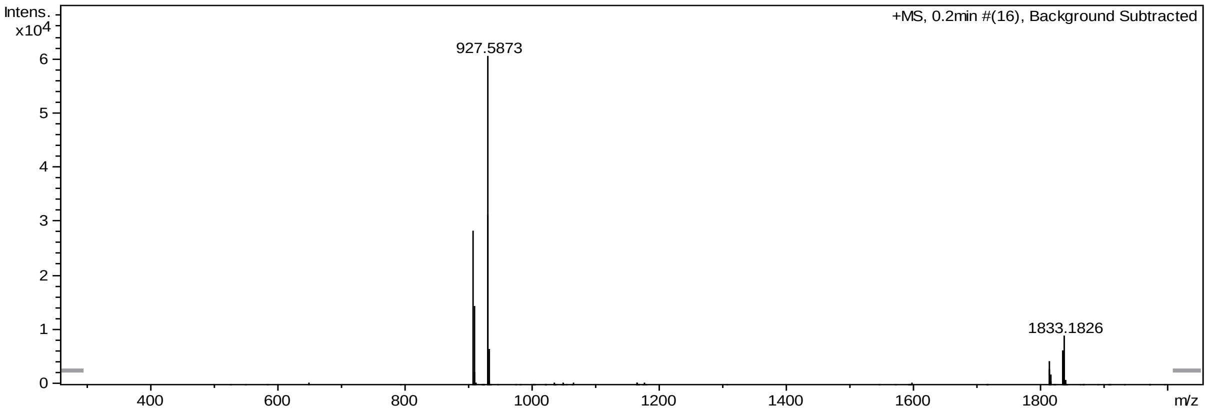

Figure S13 ^{1}H NMR, ^{13}C NMR and HRMS spectra of 4K

^{1}H NMR (400 MHz, $CDCl_3$) of 4K

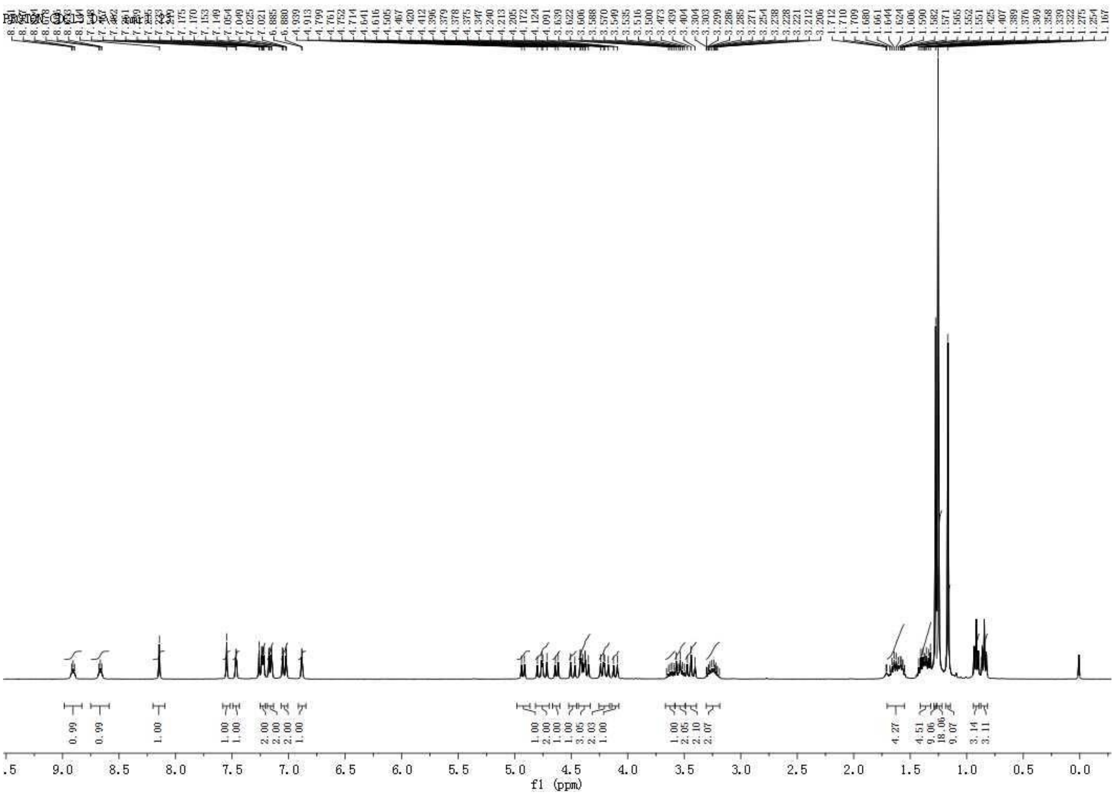

^{13}C NMR (100 MHz, $CDCl_3$) of 4K

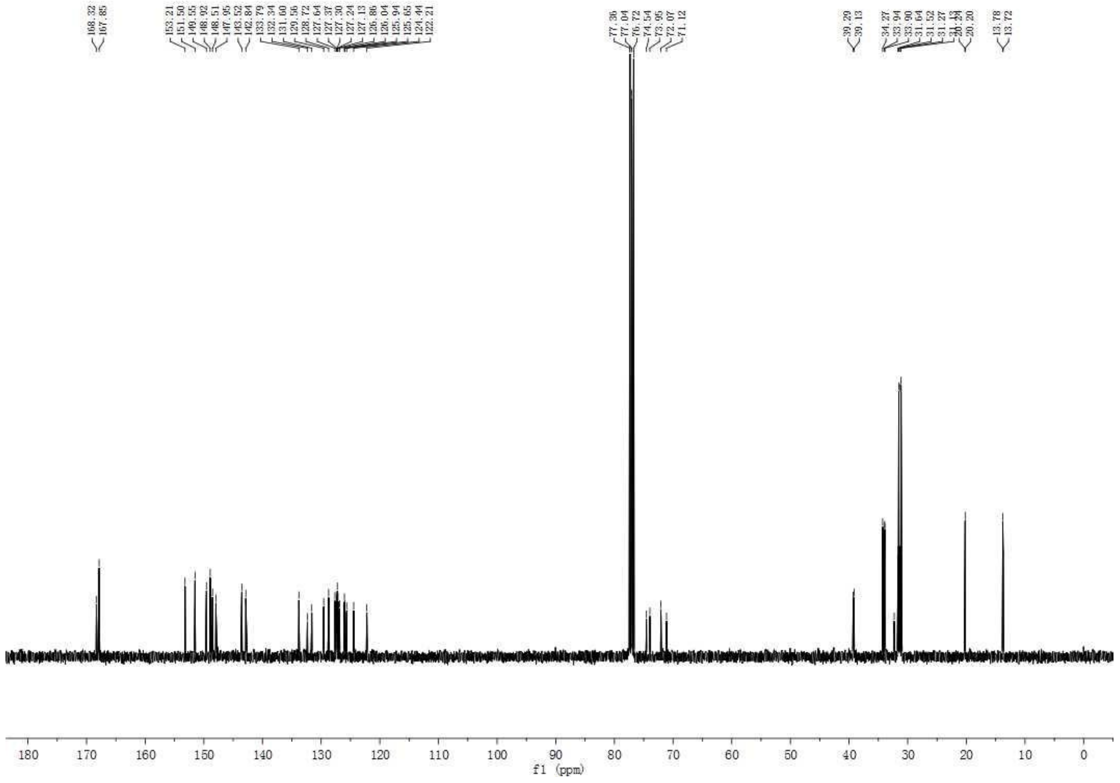

HRMS spectrum of 4K

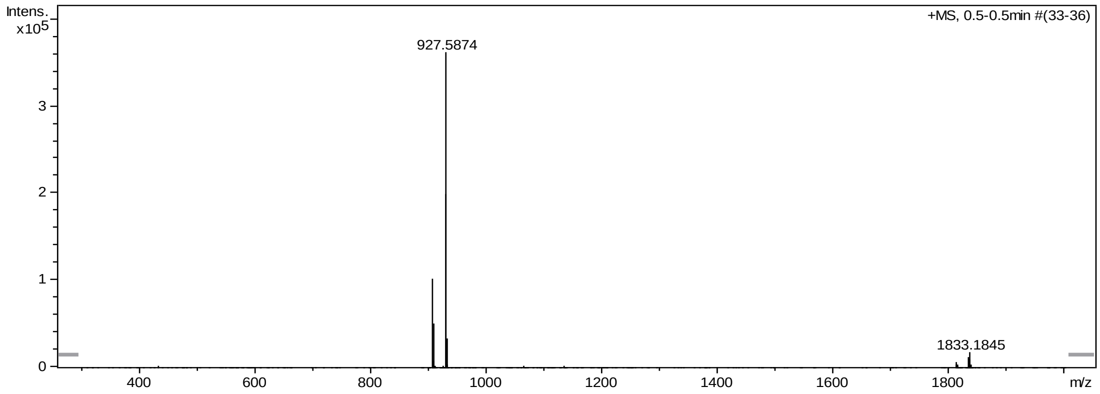

Figure S14 ^{1}H NMR,^{13}C NMR and HRMS spectra of 4L

^{1}H NMR (400 MHz, $CDCl_3$) of 4L

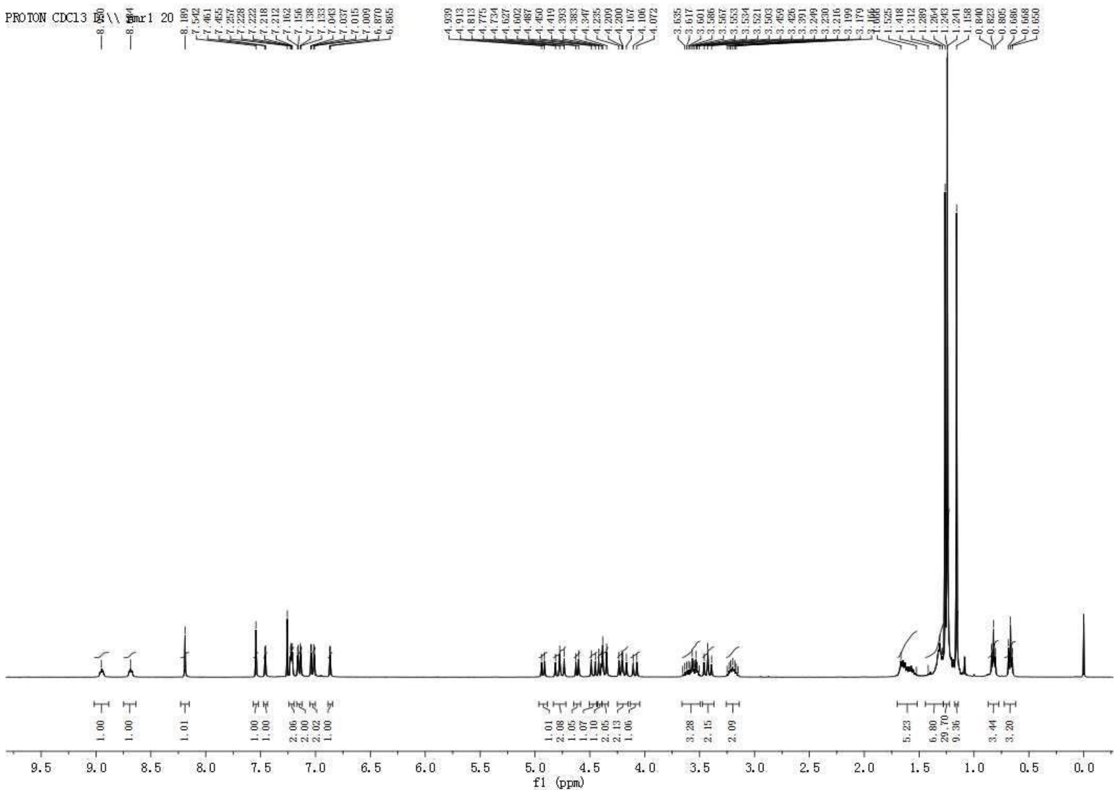

^{13}C NMR (100 MHz, $CDCl_3$) of 4L

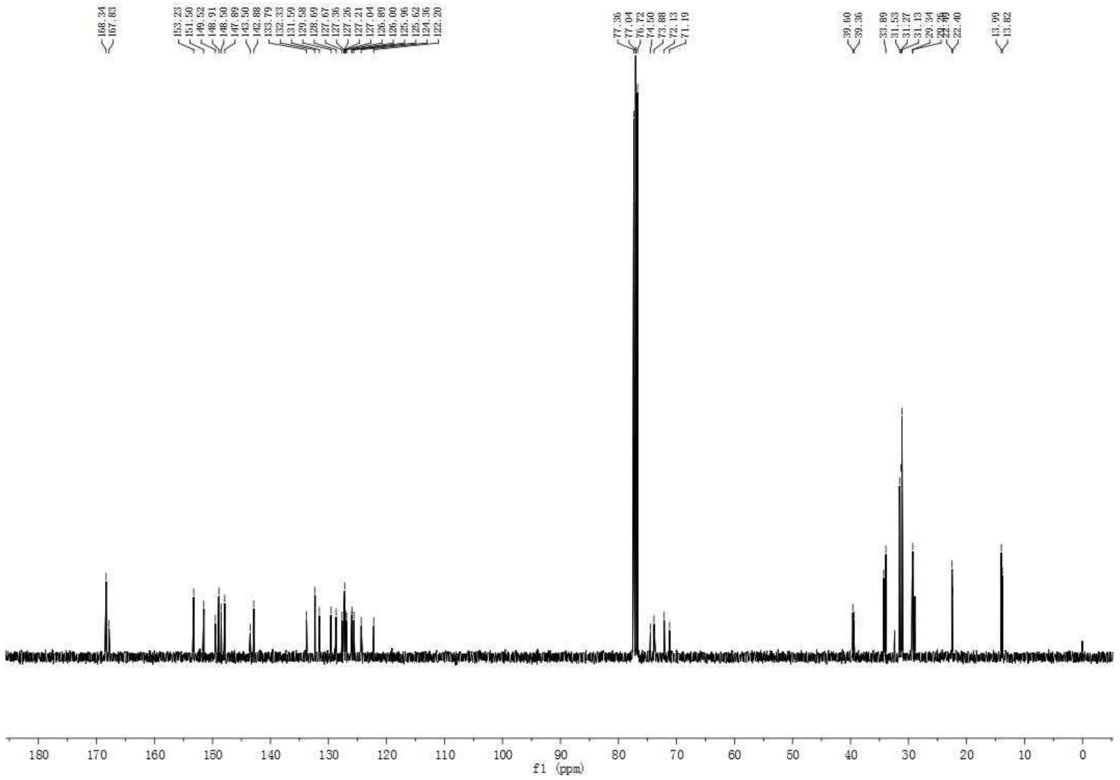

HRMS spectrum of 4L

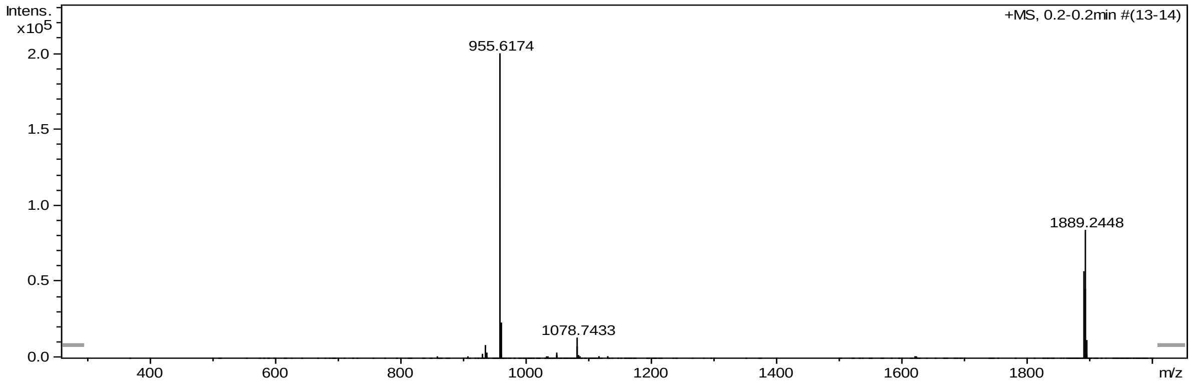

Figure S15 ^{1}H NMR,^{13}C NMR and HRMS spectra of 4M

^{1}HNMR (400 MHz, DMSO-d_6) of 4M

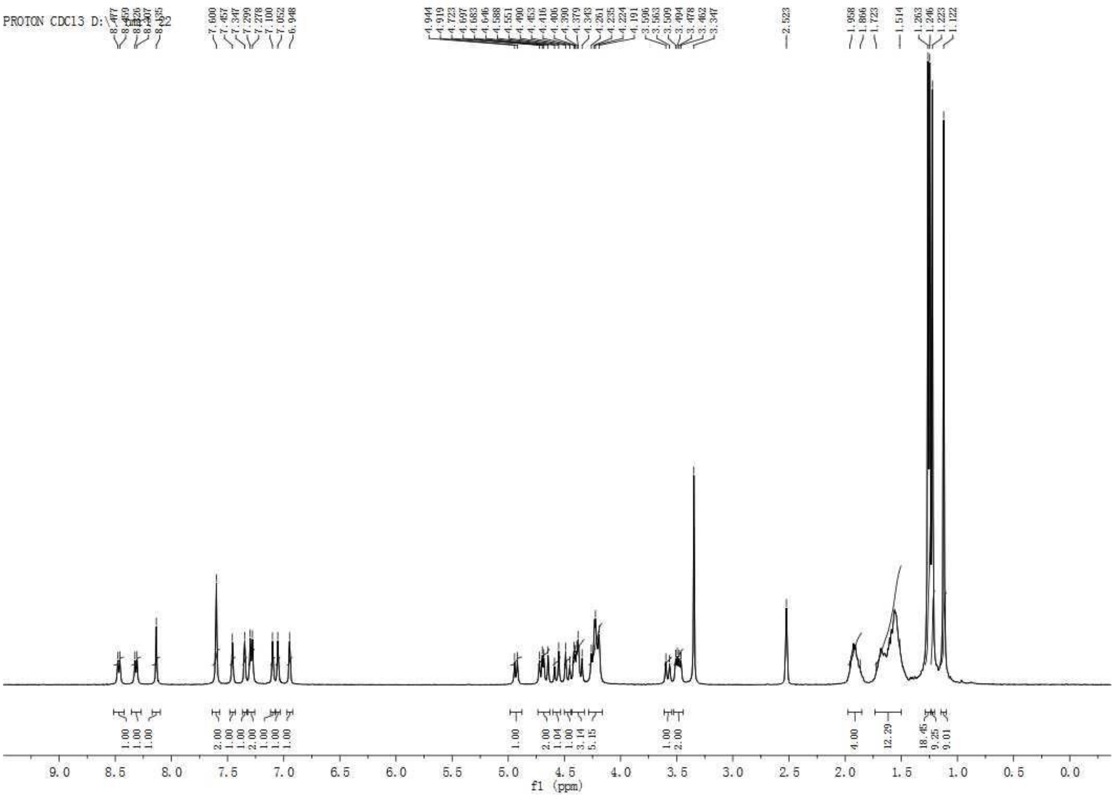

^{13}C NMR (100 MHz, DMSO-d_6) of 4M

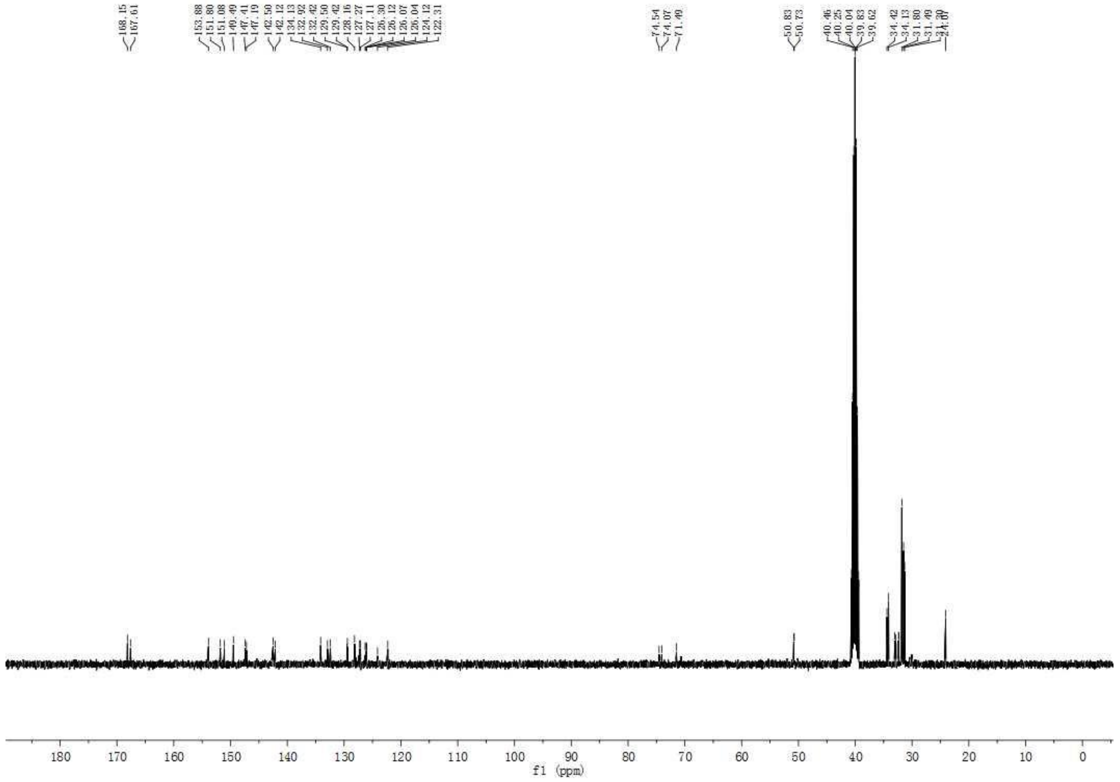

HRMS spectrum of 4M

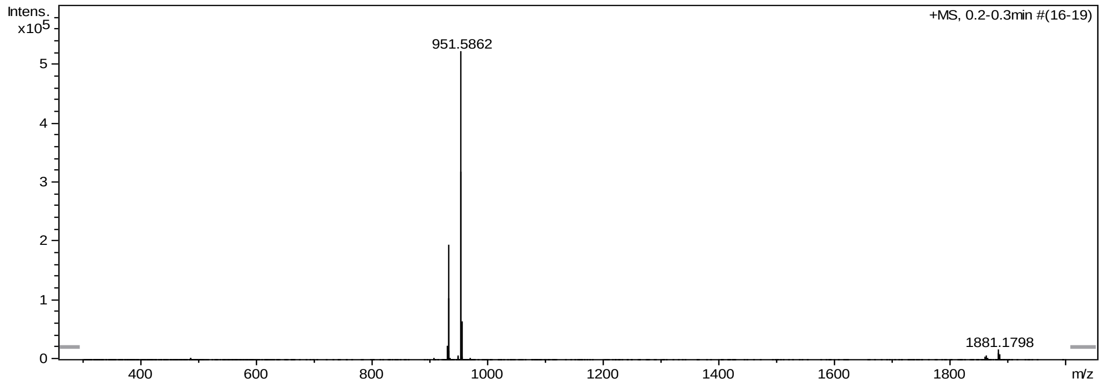

Figure S16 ^{1}H NMR,^{13}C NMR and HRMS spectra of 4N

^{1}H NMR (400 MHz, $CDCl_3$) of 4N

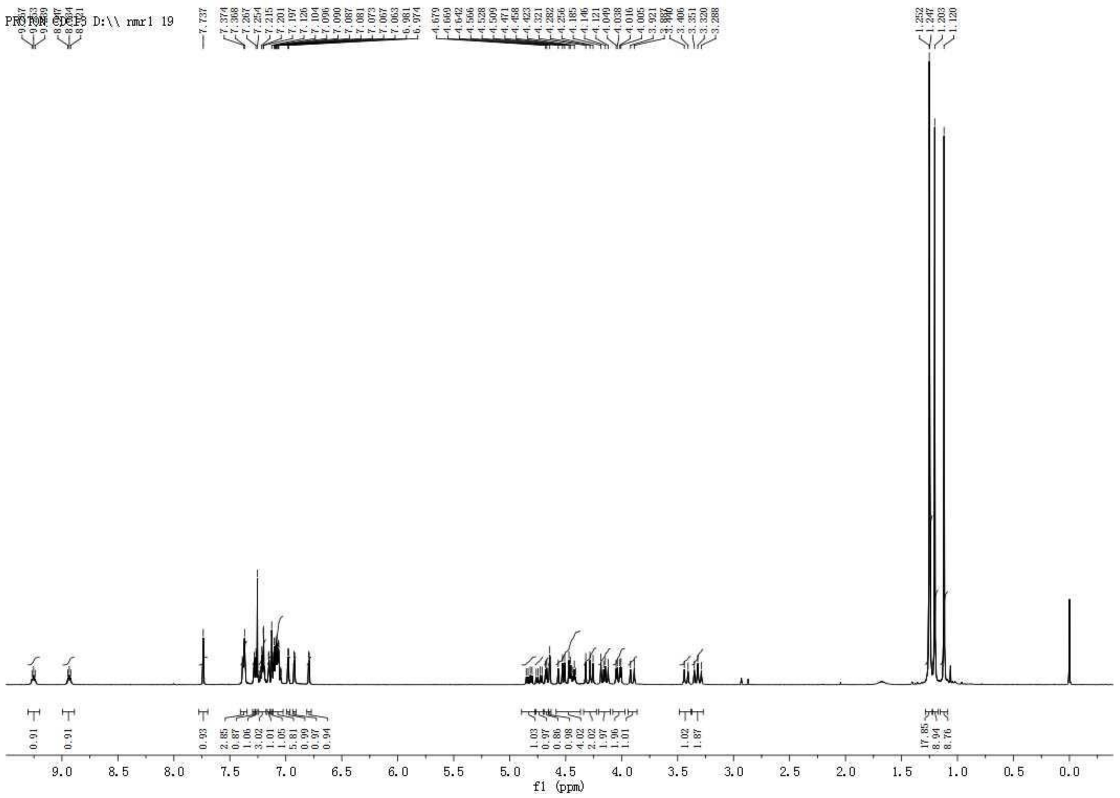

^{13}C NMR (100 MHz, $CDCl_3$) of 4N

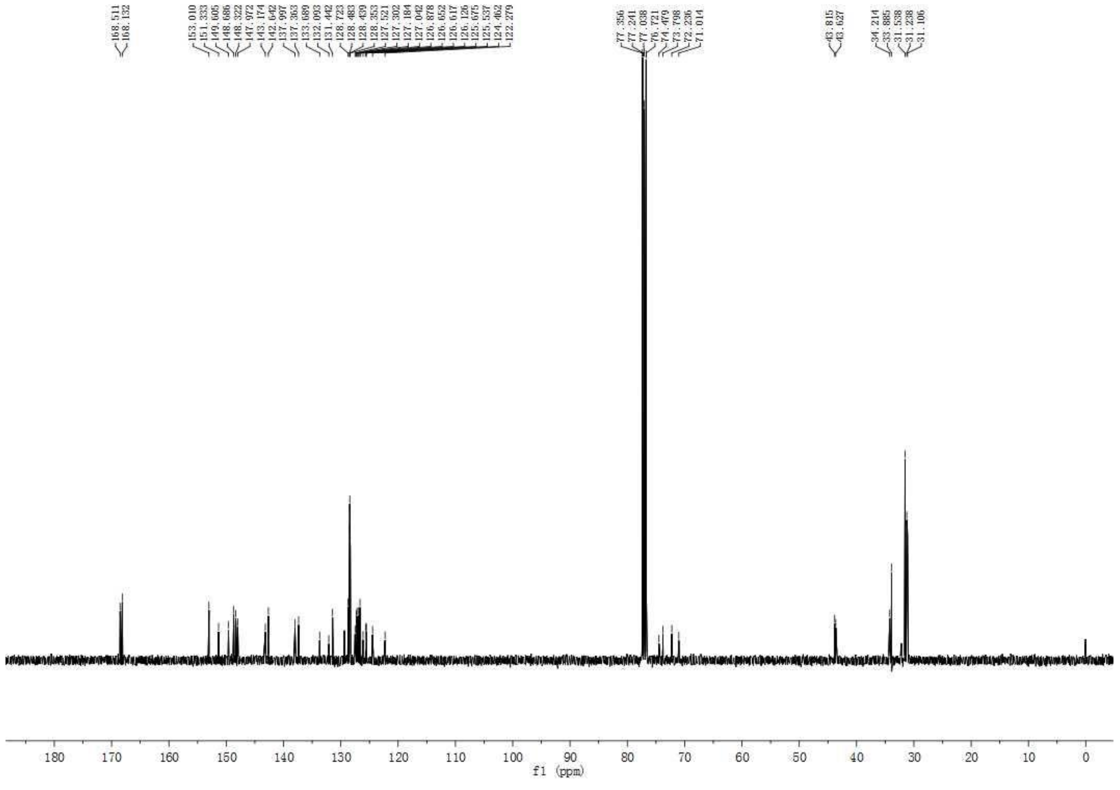

HRMS spectrum of 4N

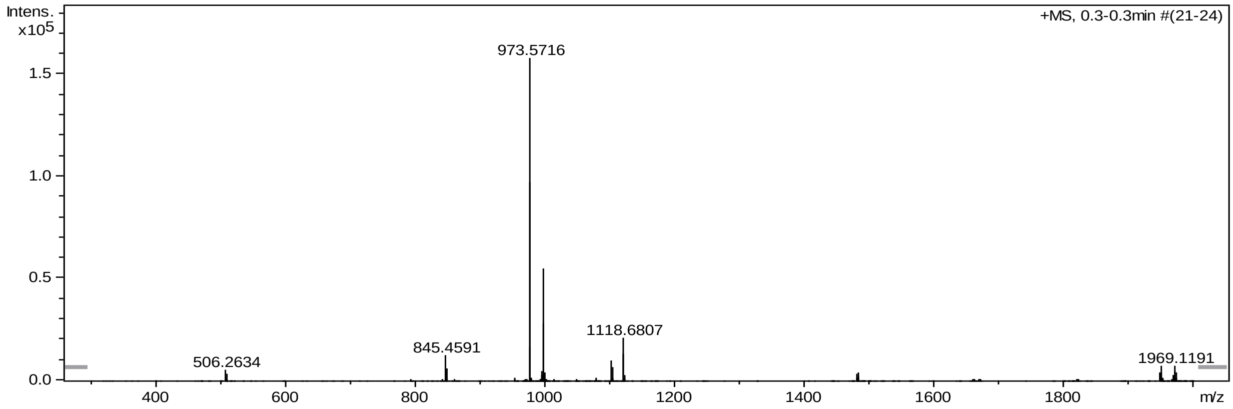

Figure S17 ^{1}H NMR, ^{13}C NMR and HRMS spectra of 4O

^{1}H NMR (400 MHz, $CDCl_3$)of 4O

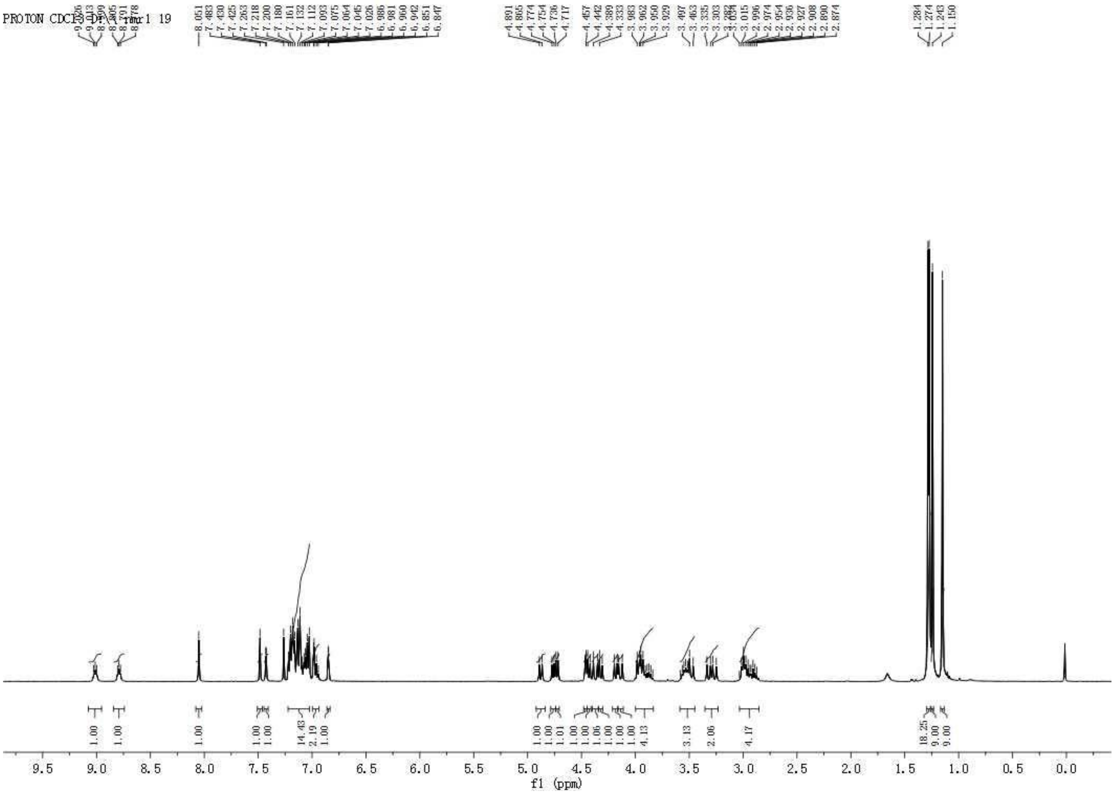

^{13}C NMR (100 MHz, $CDCl_3$) of 4O

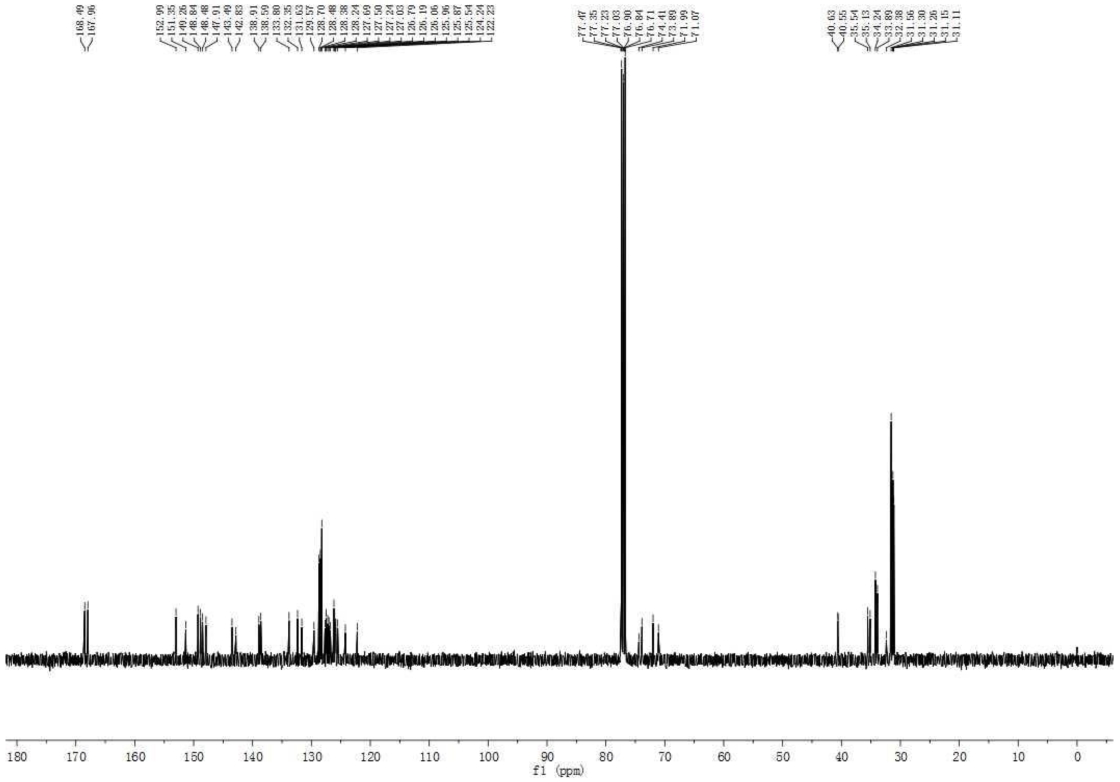

HRMS spectrum of 4O

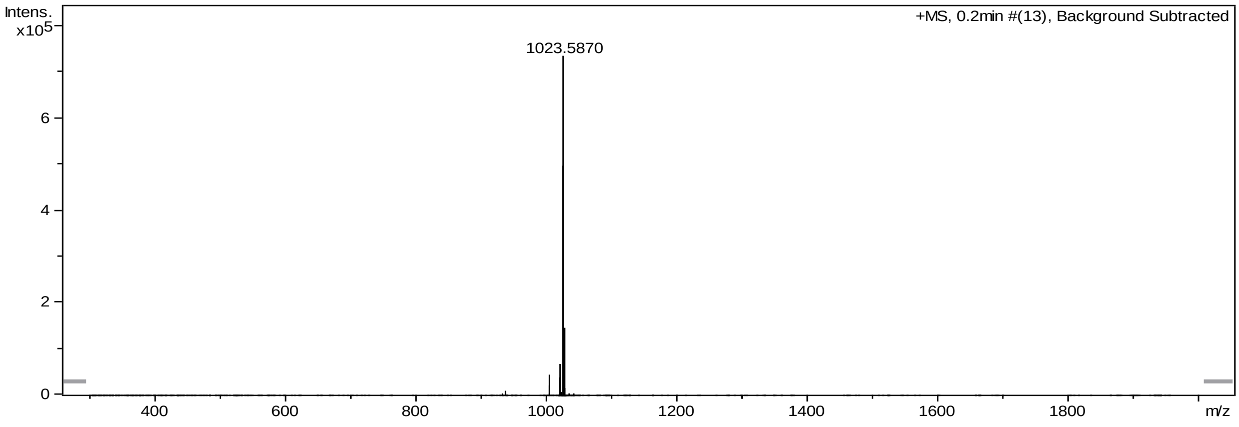

Figure S18 ^{1}H NMR, ^{13}C NMR and HRMS spectra of 4P

^{1}H NMR (400 MHz, $CDCl_3$) of 4P

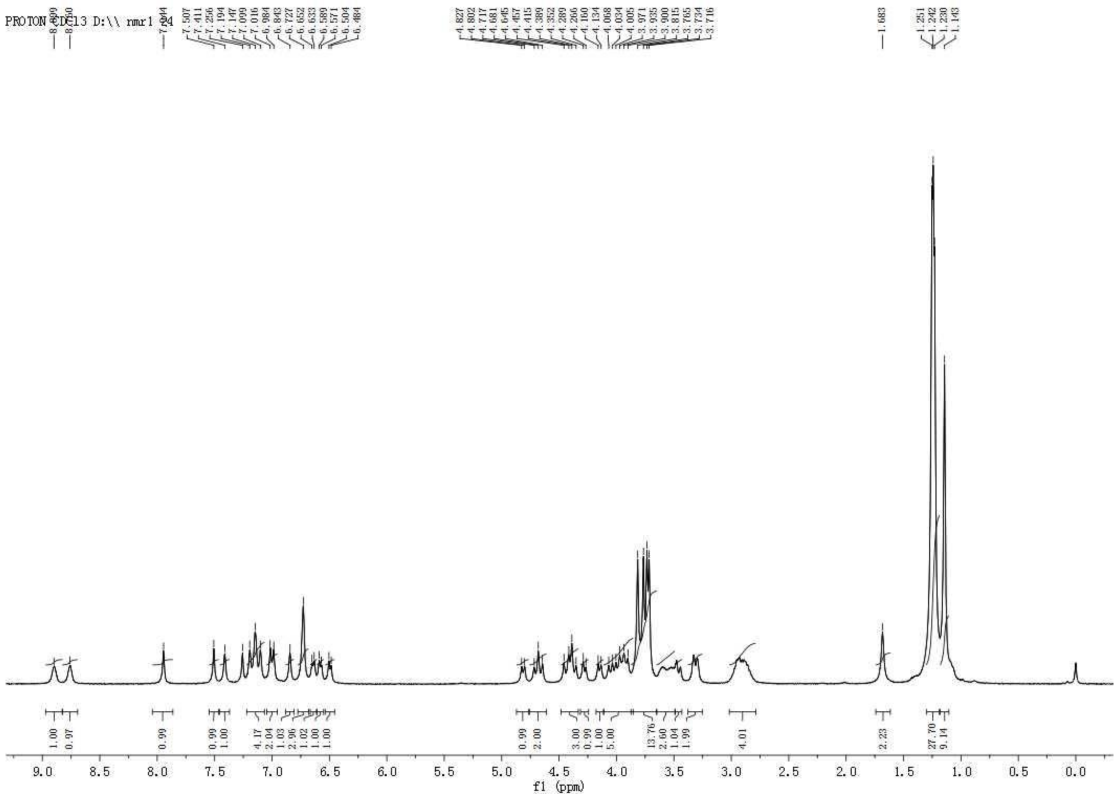

^{13}C NMR (100 MHz, $CDCl_3$) of 4P

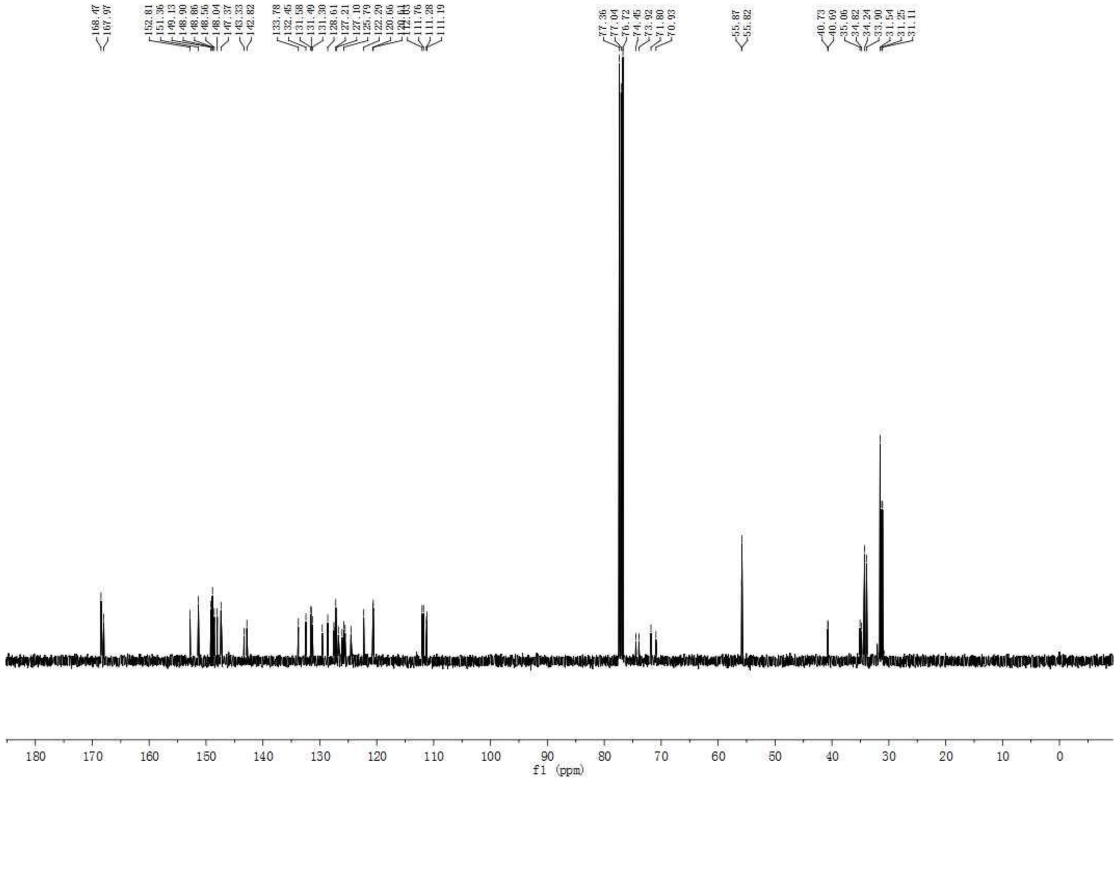

Figure S19 ^{1}H NMR,^{13}C NMR and HRMS spectra of 4Q

^{1}H NMR (400 MHz, $CDCl_3$) of 4Q

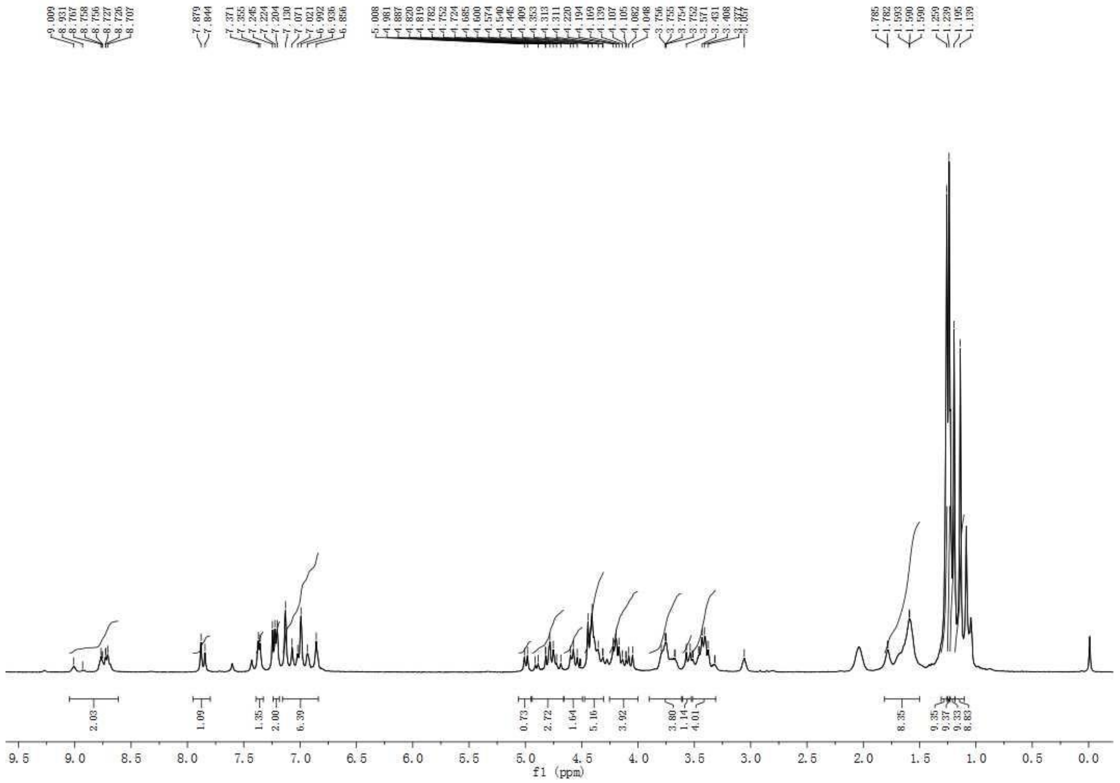

^{13}C NMR (100 MHz, $CDCl_3$) of 4Q

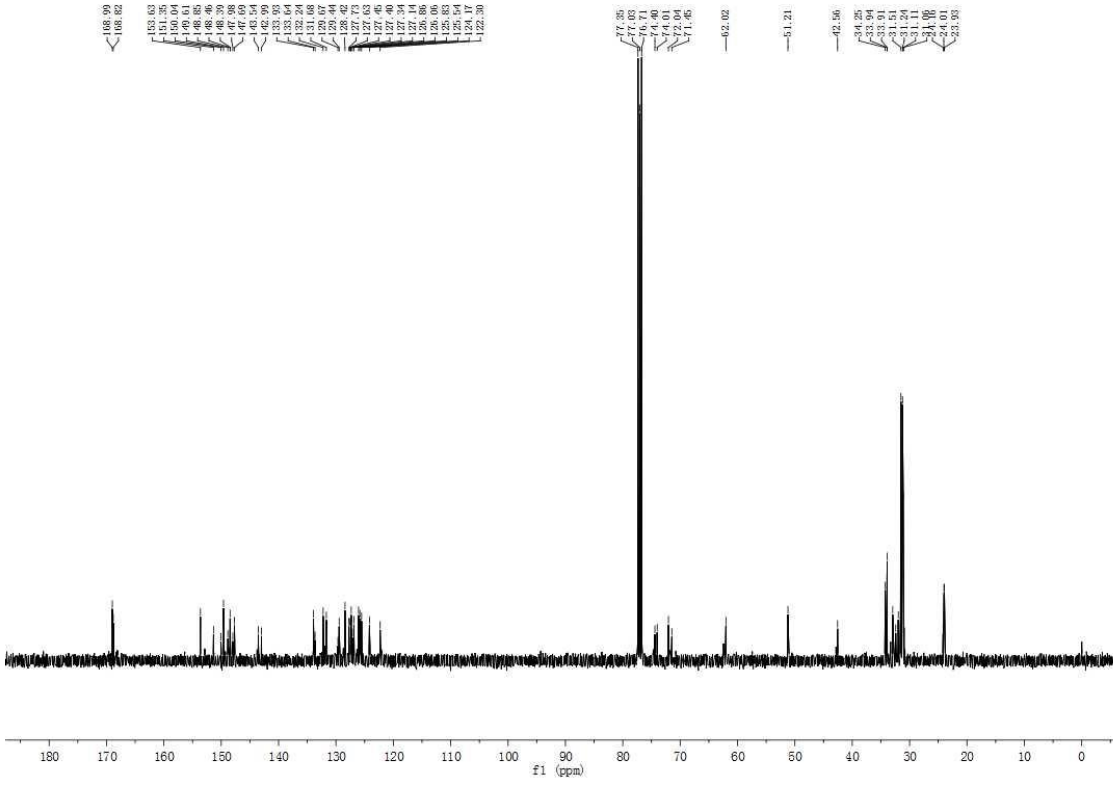

HRMS spectrum of 4Q

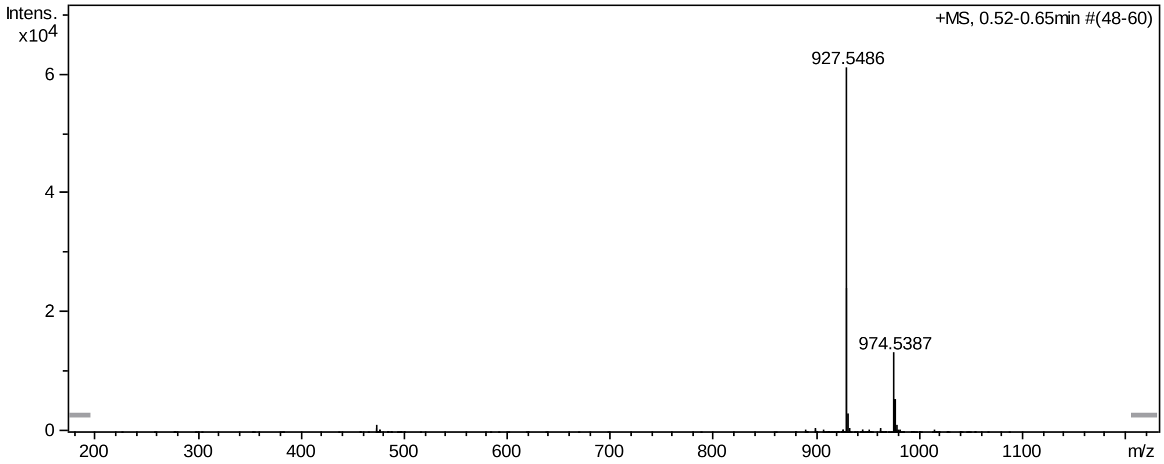

Figure S20 ^{1}H NMR, ^{13}C NMR and HRMS spectra of 4R

^{1}H NMR (400 MHz, $CDCl_3$) of 4R

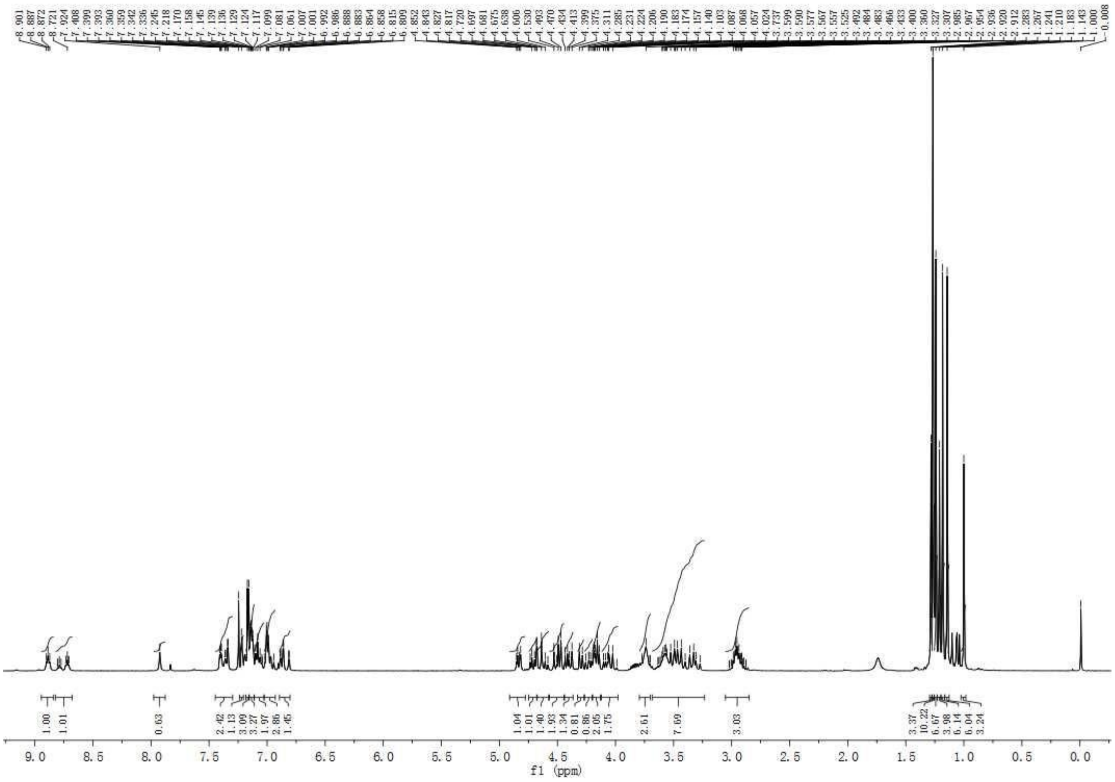

^{13}C NMR (100 MHz, $CDCl_3$) of 4R

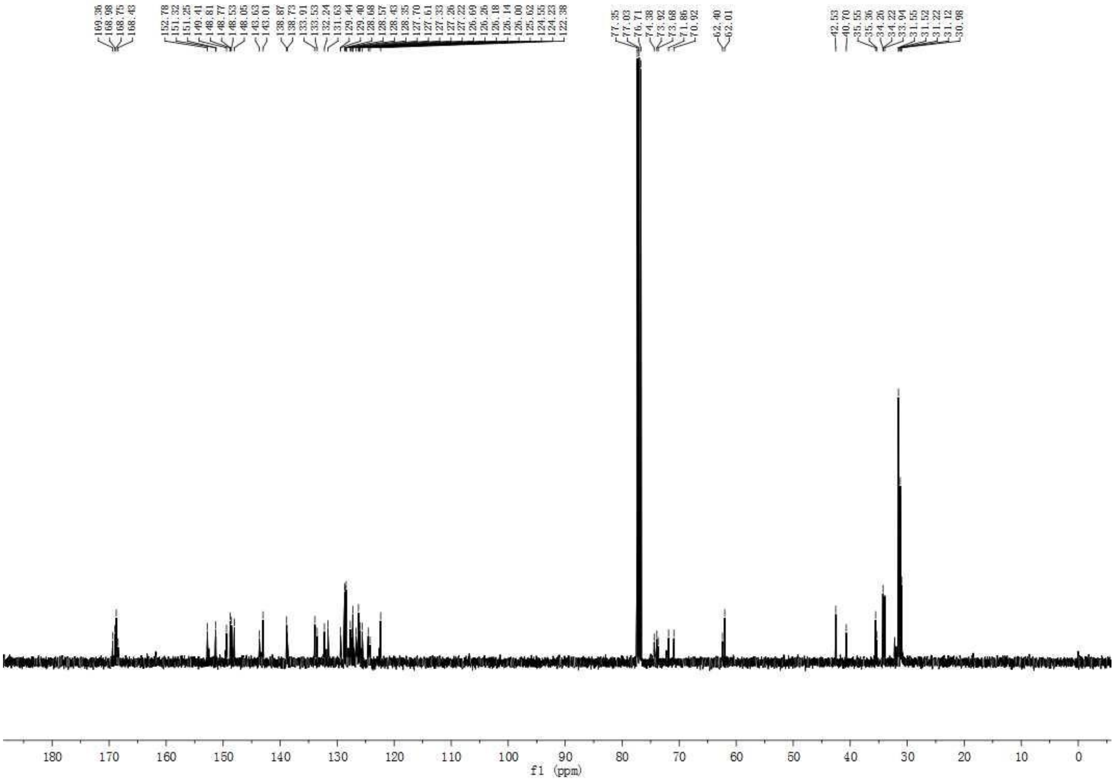

HRMS spectrum of 4R

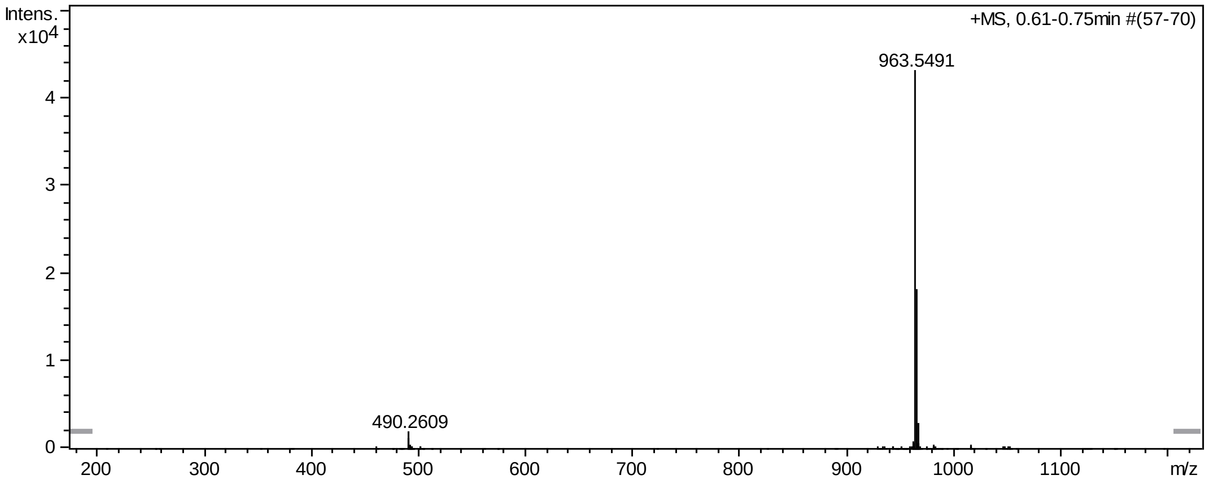

Figure S20 ^{1}H NMR,^{13}C NMR and HRMS spectra of 4S

^{1}H NMR (400 MHz, $CDCl_3$) of 4S

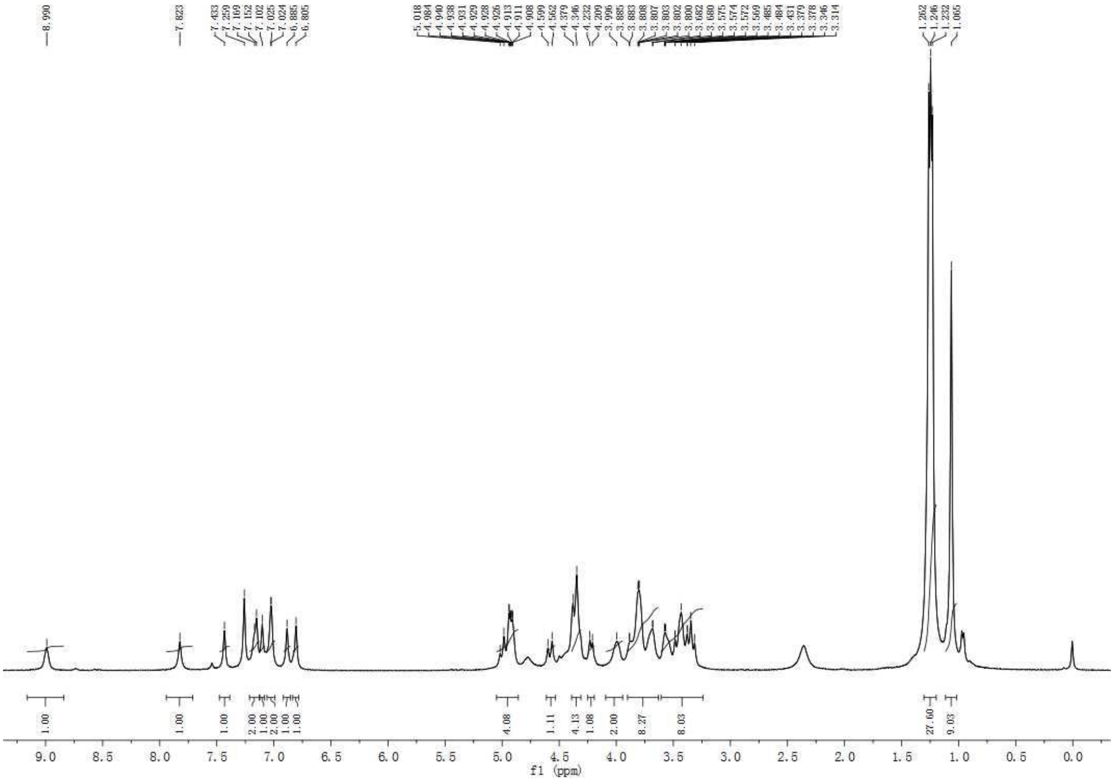

^{13}C NMR (100 MHz, $CDCl_3$) of 4S

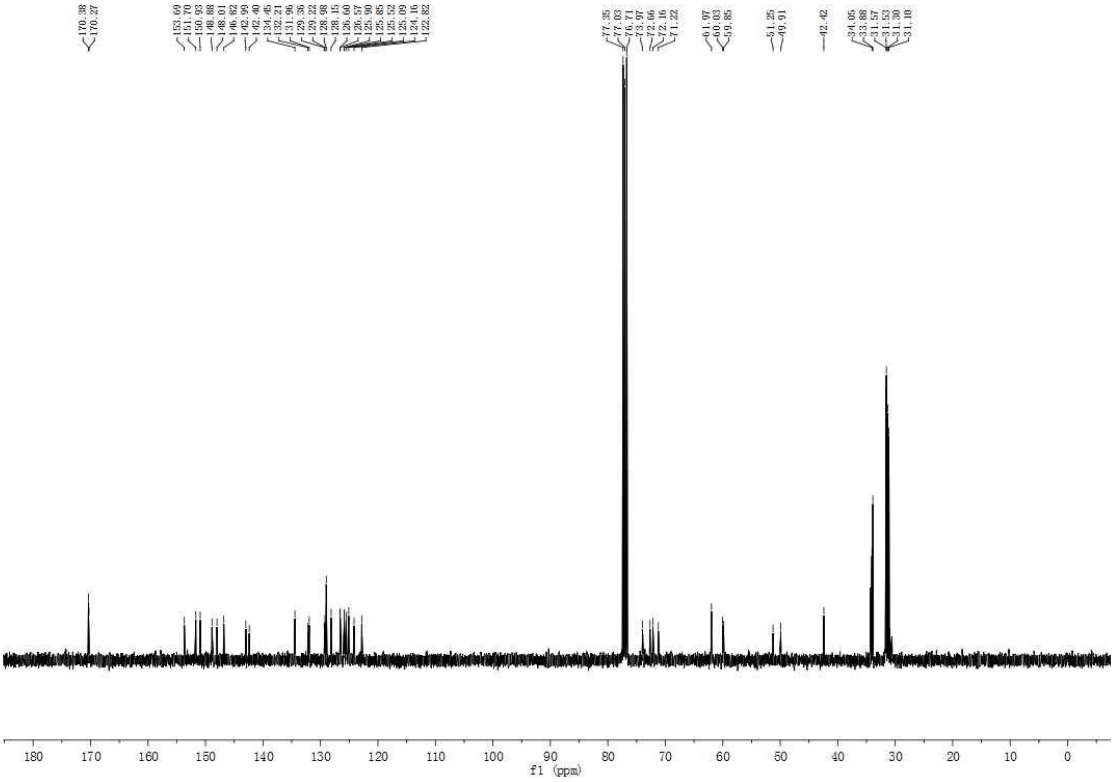

HRMS spectrum of 4S

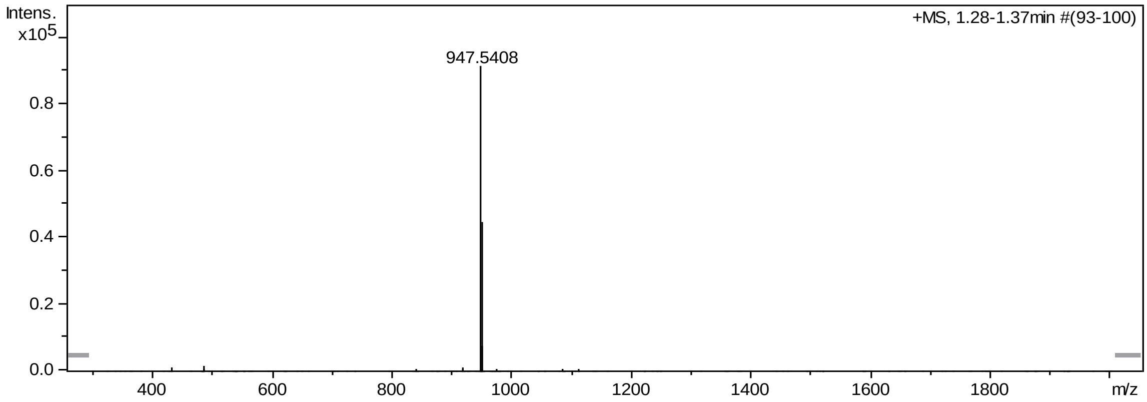

Supplement: Supplementary file 1 [file Data_Sheet_1.pdf]
